# Optimized reduced representation bisulfite sequencing reveals tissue-specific mCHH islands in maize

**DOI:** 10.1186/s13072-017-0148-y

**Published:** 2017-08-30

**Authors:** Fei-Man Hsu, Ming-Ren Yen, Chi-Ting Wang, Chien-Yu Lin, Chung-Ju Rachel Wang, Pao-Yang Chen

**Affiliations:** 1 Graduate School of Frontier Sciences, The University of Tokyo, Chiba, 277-8561 Japan; 2 Institute of Plant and Microbial Biology, Academia Sinica, Taipei, 11529 Taiwan

**Keywords:** DNA methylation, Bisulfite sequencing, Transcriptional regulation, WGBS, RRBS, Maize

## Abstract

**Background:**

DNA methylation plays important roles in many regulatory processes in plants. It is economically infeasible to profile genome-wide DNA methylation at a single-base resolution in maize, given its genome size of ~2.5 Gb. As an alternative, we adapted region of interest (ROI)-directed reduced representation bisulfite sequencing (RRBS) to survey genome-wide methylation in maize.

**Results:**

We developed a pipeline for selecting restriction enzymes in silico and experimentally showed that, in the maize genome, *MseI*- and *CviQI*-digested fragments are precisely enriched in promoters and gene bodies, respectively. We proceeded with comparisons of epigenomes and transcriptomes between shoots and tassels and found that the occurrences of highly methylated, tissue-specific, mCHH islands upstream of transcription start sites (TSSs) were positively correlated with differential gene expression. Furthermore, 5′ regulatory regions between TSS and mCHH islands often contain putative binding sites of known transcription factors (TFs) that regulate the flowering process and the timing of the transition from the vegetative to the reproductive phase. By integrating MNase-seq and siRNA-seq data, we found that regions of mCHH islands accumulate 21nt-siRNAs in a tissue-specific manner, marking the transition to open chromatin, thereby ensuring the accessibility of TFs for tissue-specific gene regulation.

**Conclusions:**

Our ROI-directed RRBS pipeline is eminently applicable to DNA methylation profiling of large genomes. Our results provide novel insights into the tissue-specific epigenomic landscapes in maize, demonstrating that DNA methylation and siRNA and chromatin accessibility constitute a critical, interdependent component that orchestrates the transition from the vegetative to the reproductive phase.

**Electronic supplementary material:**

The online version of this article (doi:10.1186/s13072-017-0148-y) contains supplementary material, which is available to authorized users.

## Background

DNA methylation is a heritable epigenetic modification that is closely associated with gene expression and chromatin structure, and it is critical in the developmental processes of animals, plants, and fungi [1–3]. In mammals, cytosine is primarily highly methylated in symmetrical CpG contexts. Yet, hypo-methylated CpG islands with an elevated CG composition are mostly found near the promoters of most housekeeping and developmentally regulated genes [4]. In plants, DNA methylation is commonly observed in both CG and non-CG sites [1], suggesting that cytosine methylation is more diverse and complex in plants.

To detect DNA methylation, sodium bisulfite is used to convert cytosines to uracils, whereas 5′-methylcytosines remain unaltered [5]. Bisulfite-treated DNA is then amplified and sequenced to determine the state of methylation. Bisulfite conversion in combination with next-generation sequencing (NGS) has become the state-of-the-art method for profiling genome-wide DNA methylation patterns at a single-base resolution [6]. However, whole-genome bisulfite sequencing (WGBS) can be very expensive when the genome size is large. Development of the reduced representation bisulfite sequencing (RRBS) has overcome this limitation by sequencing a small proportion of a large genome and has been beneficial to research in mammals, including humans, mice, and sheep [7]. Originally, RRBS was employed to sequence size-selected genomic fragments after *Msp*I enzyme digestion, by which these fragments are enriched with CpG-rich regions in the promoters of mammalian genomes. Studies have shown that methylation of such CpG islands is an important regulatory mechanism of gene silencing [8]. RRBS is very cost-effective in human studies, given that only ~1–5% of the genome is sequenced, covering ~12% of genome-wide CpG sites and ~84% of CpG islands in promoters [9, 10]. A common RRBS protocol was recently attempted in zebrafish and wasps without modification for their non-mammalian genome structures [11, 12]. Plants exhibit a different CpG distribution, lacking characteristic CpG islands in promoters. Chen et al. therefore selected *Sac*I/*Mse*I double-digested fragments from *Brassica rapa* to perform plant RRBS, which included ~2% of cytosines randomly distributed throughout the genome [13].

In plants, DNA methylation commonly occurs at cytosine bases within all sequence contexts, including symmetric CG and CHG contexts (where H = A, T, or C) and asymmetric CHH contexts [1]. Research in *Arabidopsis* has demonstrated that different genetic pathways involving different DNA methyltransferases specifically regulate CG, CHG, and CHH sites [14]. Methyltransferase1 (MET1) and chromomethylase3 (CMT3) primarily maintain CG and CHG methylation, respectively. CHH methylation is maintained by the RNA-directed DNA methylation (RdDM) pathway, involving domains rearranged methyltransferase 1/2 (DRM1/2), chromomethylase2 (CMT2) and small interfering RNA [15]. RdDM requires transcription machinery composed of RNA polymerases Pol IV and Pol V, which are plant specific, and acts as the major small RNA-mediated epigenetic pathway in plants [16]. Studies have shown that DNA methylation plays an important role in the direct regulation of genes during plant development and stress responses [17]. Both tissue- and cell-type-specific DNA methylation patterns have been reported in plants. For example, a recent study demonstrated that specific changes in methylation during maize leaf development promote tissue-specific regulation of particular genes [18].

The maize genome is 20-fold larger than that of *Arabidopsis,* and more than 80% of the sequence is composed of transposable elements (TEs), distributed throughout the genome [19]. Thus, more than 85% of annotated genes are located near TEs in maize [20]. Compared with *Arabidopsis,* maize exhibits higher methylation levels in all contexts (in unfertilized ears, mCG = 86%, mCHG = 74%, mCHH = 5.4%), with CG and CHG methylation being abundant in intergenic regions [21]. Interestingly, genomic profiling of CHH methylation in maize has revealed particular enrichment within 1 kb upstream of transcription start sites (TSSs) and 1 kb downstream of transcription end sites (TESs), referred to as mCHH islands [21]. Li et al. [22] further characterized mCHH islands as 100-bp tiles with an average CHH methylation level of at least 25%. These islands were suggested to indicate the transition between heterochromatin-associated TEs and euchromatin-associated genes [22]. In contrast to the suppressive role of DNA methylation in promoter regions observed in most studies of various organisms, elevated mCHH islands are found in expressed genes that are located near TEs [21, 22]. However, loss of CHH methylation in mutants does not impact gene expression but leads to an additional loss of CG and CHG methylation in flanking transposons, suggesting that promoter methylation at CHH islands in maize may not simply control gene expression but may also regulate nearby *cis* elements [22].

Maize is a model system for studying epigenetic phenomena such as imprinting, paramutation, and TE inactivation [23, 24]. During reproduction, maize develops separate unisexual flowers, taking the form of tassels (male inflorescences) and ears (female inflorescences). Following the development of all leaf primordia, the shoot apical meristem is transformed into the tassel meristem to give rise to the tassel primordium. The development of tassels indicates the transition from vegetative growth to the reproductive stage, which depends on genetic control of a gene expression network in response to environmental cues [25]. Studies conducted in past decades have revealed that epigenetic pathways are important for plant reproduction [26]. However, the relationship between genome-wide DNA methylation and gene expression networks during floral development remains largely unexplored.

WGBS is not cost-efficient in maize because of its large genome (2.5 Gb). Here, we provide a systematic approach for adapting RRBS to maize, one of the world’s major crops, by selecting new RRBS restriction enzymes and optimizing the range of fragment sizes to target genomic regions of interest (ROIs). Our approach takes into account both the size of the enzyme-digested fragments and enrichment of these fragments in ROIs, allowing us to survey different parts of the genome. We identified two restriction enzymes, *Mse*I and *Cvi*QI, which were predicted to significantly enrich promoter and genebody regions, respectively. In our pipeline, the maize RRBS data can be mapped by general bisulfite aligner such as BS Seeker 2 [27], and the downstream methylome analysis by the analyzer such as MethGO [28].

As a first attempt, we compared DNA methylation profiles between shoot and tassel primordia to investigate methylation changes in vegetative and reproductive stages. We took advantage of our ROI-directed RRBS method to specifically reveal tissue-specific mCHH islands and found that genes with mCHH islands tended to be up-regulated. This up-regulation of gene expression was positively correlated with nearby TE methylation. Finally, by incorporating MNase-seq, we observed that mCHH islands localized to open chromatin transition regions, which might lead to exposure of transcription factor binding sites (TFBSs). The present study not only provides valuable insights into the possible functions of DNA methylation during floral development in maize, but further characterizes the critical features of mCHH islands.

The in silico enzyme selection pipeline in this study has been deposited to GitLab at https://gitlab.com/fmhsu0114/maize_RRBS/tree/master.

## Results

### Identification of restriction enzymes for maize RRBS

**Fig. 1:**
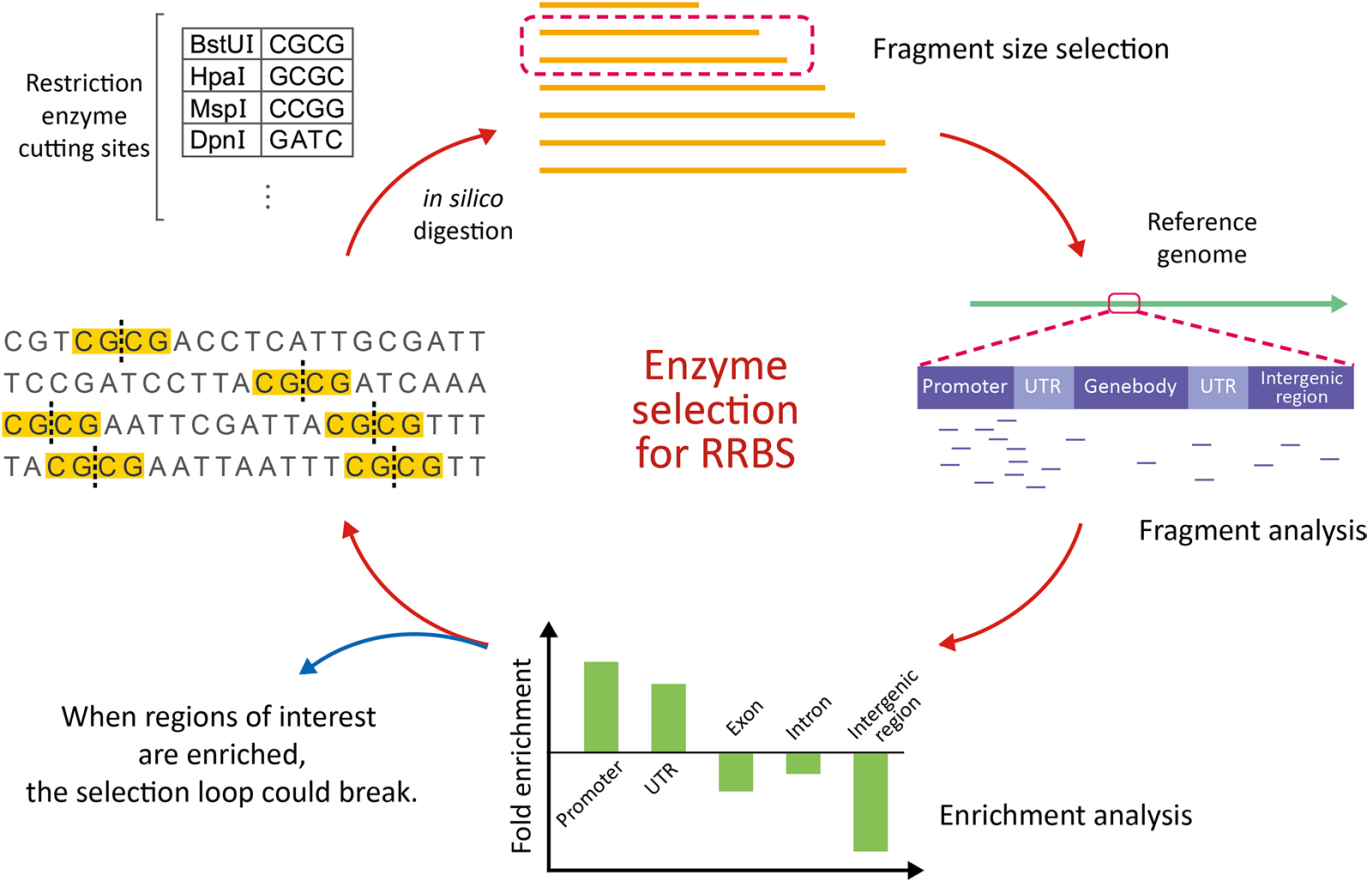
Schematic flowchart of maize RRBS. Candidate restriction enzymes are selected by jointly considering the selected range of fragment size, and their enrichment toward ROIs. Enrichment of a specific genomic ROI (e.g., promoter) is measured by comparing the percentage of ROI in the RR genome against that in the reference genome. The ideal enzymes and the range of fragment sizes were decided when the ROIs were clearly enriched

The first step in RRBS library preparation is restriction enzyme digestion [9]. In the mammalian RRBS protocol, *Msp*I is utilized to enrich for CpG-rich regions in promoters. This same method was used in the zebrafish genome to enrich for 5.3% of CpG sites in a 2.2% reduced representation genome [11]. In plants, the cytosine distribution is different from that in vertebrates, and no CpG islands have been reported. Therefore, *Msp*I may not be suitable for plant genomes. First, we selected candidate restriction enzymes that are methylation insensitive to avoid low cutting efficiency due to DNA methylation at cutting sites. By jointly considering the enzyme-digested fragments and the selected range of fragment size, we estimated the enrichment of fragments in the target regions and the sequencing cost. The ideal enzymes and the range of fragment sizes were decided when the ROIs were clearly enriched with minimum sequencing (see Fig. 1 for the schematic flowchart of enzyme selection in RRBS).

**Fig. 2:**
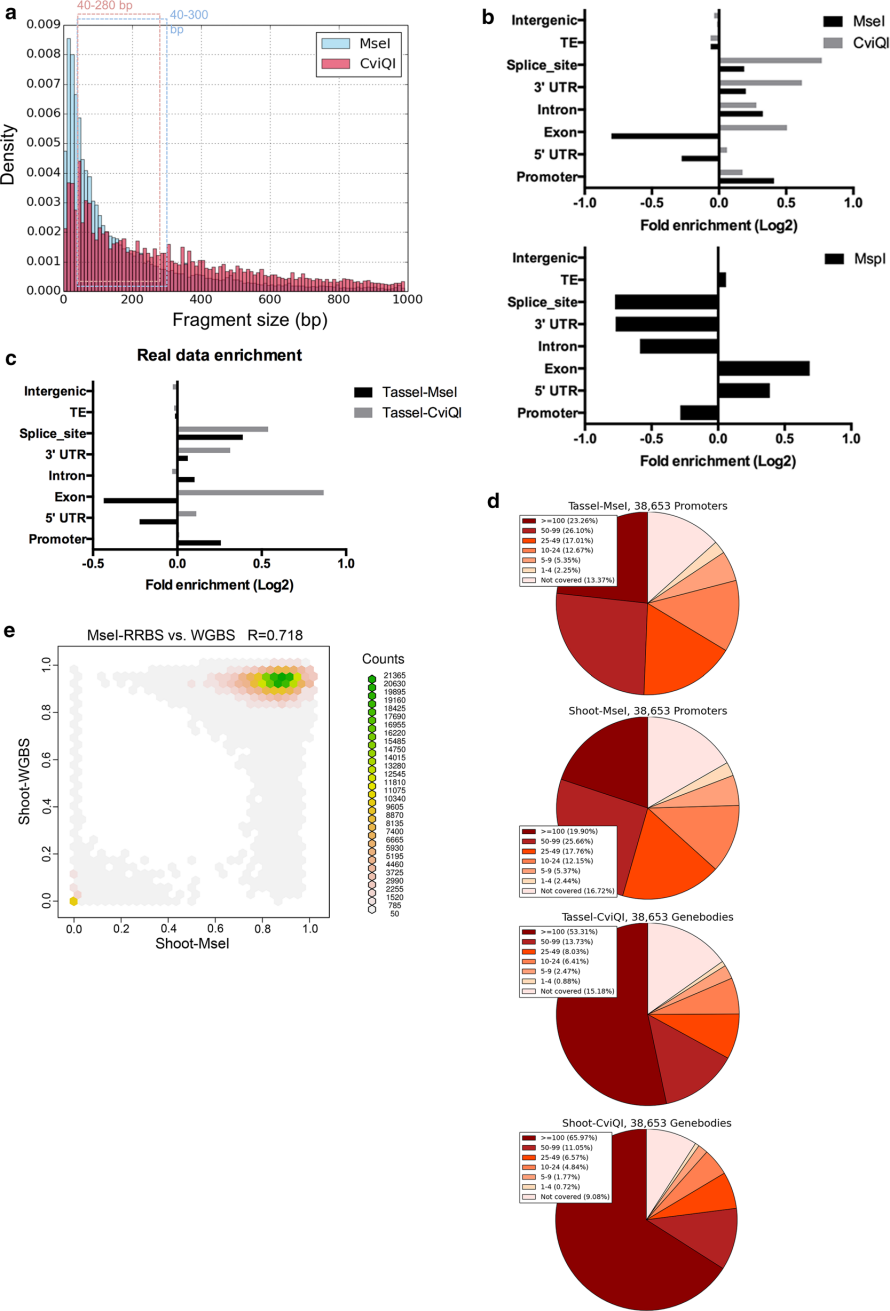
Prediction and validation of maize RRBS. **a** Fragment distribution and size selection of *Mse*I- and *Cvi*QI-RRBS. **b** Enrichment analysis of predicted *Mse*I-, *Cvi*QI-, and *Msp*I-digested fragments in genomic regions. **c** Enrichment analysis of sequenced tassel-*Mse*I and tassel-*Cvi*QI fragments in genomic regions. **d** Pie charts of the four RRBS libraries show coverage in promoters (*top 2*, *Mse*I-RRBS) and in genebody (*bottom 2*, *Cvi*QI-RRBS). **e** Correlation between shoot-*Mse*I with shoot-WGBS

We selected restriction enzymes for the best promoter enrichment. A total of 85 restriction enzymes (Additional file 1), including both 4 and 6 cutters, were analyzed through in silico digestion. The fragments digested by each enzyme were generated and subjected to enrichment analysis to evaluate their enrichment in genomic features, including TEs, promoters, exons, introns, splicing sites, and untranslated regions (UTRs). We determined the promoter region as 1000 bp upstream of TSS based on Ensembl AGPv3 annotation. Among them, *Mse*I, which recognizes TTAA sites, was found to digest the maize genome into evenly distributed fragments that are enriched in promoters (Fig. 2a). To assess the enrichment outcome in different size ranges, we set the size range of 100–250 bp as a baseline and extended the values to determine lower and upper bounds (Additional file 2: Table S1 shows the genome and promoter contents of each set of fragments after *Mse*I digestion). We selected 40–300 bp as the RRBS size selection boundary that exhibited high enrichment for promoter regions. The total size of these *Mse*I-digested fragments was predicted as 566 Mbp, accounting for 25.6% of the genome and covering 84% of promoters. That is, among the 38,653 gene promoters, 84% have one or more cytosines covered by our fragments. These fragments exhibited a significant 1.3-fold enrichment in promoters (Fig. 2b). We also searched for enzymes that could enrich different genomic features. *Cvi*QI recognizes GTAC sites, and *Cvi*QI-digested fragments of 40–280 bp covered 270 Mbp (12.7%) of the genome. This enzyme produced 1.4- and 1.2-fold enrichment in exons and introns (defined as the gene body), respectively (Fig. 2a, b). Since the genome size is reduced and the sequencing cost is proportional to the genome size, the expected cost of RRBS is greatly reduced from that of WGBS (Additional file 2: Table S2). For comparison, we estimated enrichment in the maize genome using *Msp*I, which is employed in mammalian RRBS studies, and found that *Msp*I fragments were actually deprived of maize promoters, indicating that *Msp*I is not suitable for maize promoter analysis (Fig. 2b). Our findings suggest that RRBS enzymes must be carefully selected for different genomes and different ROIs. Therefore, we selected *Mse*I for promoter enrichment and *Cvi*QI for gene body enrichment.

### Maize RRBS using *Mse*I and *Cvi*QI significantly enriches for promoter and gene body regions, respectively

To compare DNA methylation patterns in different developmental stages in maize, we chose to examine the shoot and tassel primordia. Shoots at the coleoptile stage represent early vegetative tissue, whereas the tassel primordium, the earliest stage of male flower development, is the first reproductive tissue transformed from the shoot apex in maize (Additional file 3: Figure S1). We constructed promoter-enriched (*Mse*I-digested) and gene body-enriched (*Cvi*QI-digested) RRBS libraries using shoot and tassel primordium samples. Hereafter, *Mse*I-RRBS and *Cvi*QI-RRBS refer to the two RRBS profiles generated with *Mse*I and *Cvi*QI, targeting promoters and gene bodies, respectively. For tissue comparisons, we use shoot-*Mse*I, tassel-*Mse*I, shoot-*Cvi*QI and tassel-*Cvi*QI to indicate the tissue and the RRBS enzyme employed in our libraries.

We sequenced more than 32 million reads of 101 bp in each RRBS sample, covering 13–20% of the maize genome (Table 1). We also processed two previously published WGBS datasets from shoots as benchmark data [29]. In our pipeline, we used BS Seeker 2 [27] for mapping both WGBS and RRBS with *Mse*I and *Cvi*QI sites. Similar to the WGBS results, approximately 52% of the reads from our RRBS data were uniquely mapped to the maize reference genome AGPv3 (Table 1). By analyzing the reads mapped to lambda phage spiked in our libraries, we estimated the bisulfite conversion rates to be greater than 99%.

**Table 1:** Mapping statistics

Tissue	Library type	Enzyme	Sequencing mode	Number of raw reads	Number of uniquely mapped reads	Mappability (%)	Genome coverage (%)	Bisulfite conversion rate (%)
Shoot	RRBS	*Mse*I	PE	31,927,914	16,647,292	52.14	18.68	99.33
Tassel	RRBS	*Mse*I	PE	67,346,177	35,605,841	52.87	19.52	99.43
Shoot	RRBS	*Cvi*QI	PE	61,099,496	34,007,664	55.66	16.95	99.57
Tassel	RRBS	*Cvi*QI	PE	35,907,828	19,498,226	54.30	13.19	99.49
Shoot1	WGBS	–	PE	96,977,894	64,310,359	66.31	91.91	–
Shoot2	WGBS	–	SE	282,553,129	143,740,327	50.87	79.01	–

Both *Mse*I and *Cvi*QI generate sticky-ended DNA fragments with gaps that are filled later during library construction. On average, 97% of the *Mse*I-RRBS reads from the forward strand and 95% of the reads from the complementary strand began with TAA, which corresponds to the *Mse*I cutting site (Additional file 3: Figure S2). For *Cvi*QI-RRBS, 94% of read1 sequences began with TAT or TAC, and 91% of read2 sequences started with TAC. These results demonstrate the high accuracy of our enzyme digestion procedure and the high efficiency of end repair during library construction.

To evaluate experimental enrichment of our RRBS libraries in the target regions, we analyzed our tassel-*Mse*I and tassel-*Cvi*QI-RRBS data (Fig. 2c). The tassel-*Mse*I data revealed fragments enriched in promoters and splice sites, as expected from our in silico analysis (Fig. 2b, c). *Cvi*QI-RRBS showed significant enrichment of exons and splice sites, also as predicted (Fig. 2b, c). In total, our RRBS libraries covered 82–115 M cytosines (Additional file 2: Table S3), which is a much higher number than for other targeted methylation arrays, such as Roche NimbleGen arrays that cover 270 k, 1.4 M or 2.1 M custom probes [30].

We then analyzed the coverage of *Mse*I-RRBS and *Cvi*QI-RRBS at promoters and gene bodies, respectively. Of the 38,653 annotated genes, tassel-*Mse*I and shoot-*MseI*-RRBS covered 86.63 and 83.28% of all promoters, respectively. Similarly, *Cvi*QI-RRBS covered more than 84% of gene bodies (Fig. 2d). These results strongly suggest that our RRBS method is effective in covering the ROIs as in our in silico predictions. In more than 44% of promoters and 46% of gene bodies, our RRBS results covered more than 50 CHH sites (Fig. 2d), suggesting good coverage within each promoter and gene body. Given that *Mse*I-RRBS and *Cvi*QI-RRBS are designed to enrich different regions of the genome, only ~1.9% of cytosine sites overlapped (Additional file 2: Table S3), suggesting that our method precisely targets specific ROIs.

As a validation of our shoot RRBS, we compared our shoot-*Mse*I-RRBS results with two sets of shoot-WGBS data (Additional file 3: Figure S3 ). Figure 2e presents a correlation plot of the CG methylation level per cytosine between RRBS and WGBS. CG methylation exhibits a bimodal distribution. CpG sites are either hyper- or hypo-methylated in most of the maize genome, whereas intermediately methylated regions are less frequently observed. This result indicates that *Mse*I-RRBS is well correlated with WGBS (*r* = 0.718). The same trend was observed for *Cvi*QI-RRBS when compared with WGBS (*r* = 0.824).

### Comparison of DNA methylation patterns between shoot and tassel primordia

**Fig. 3:**
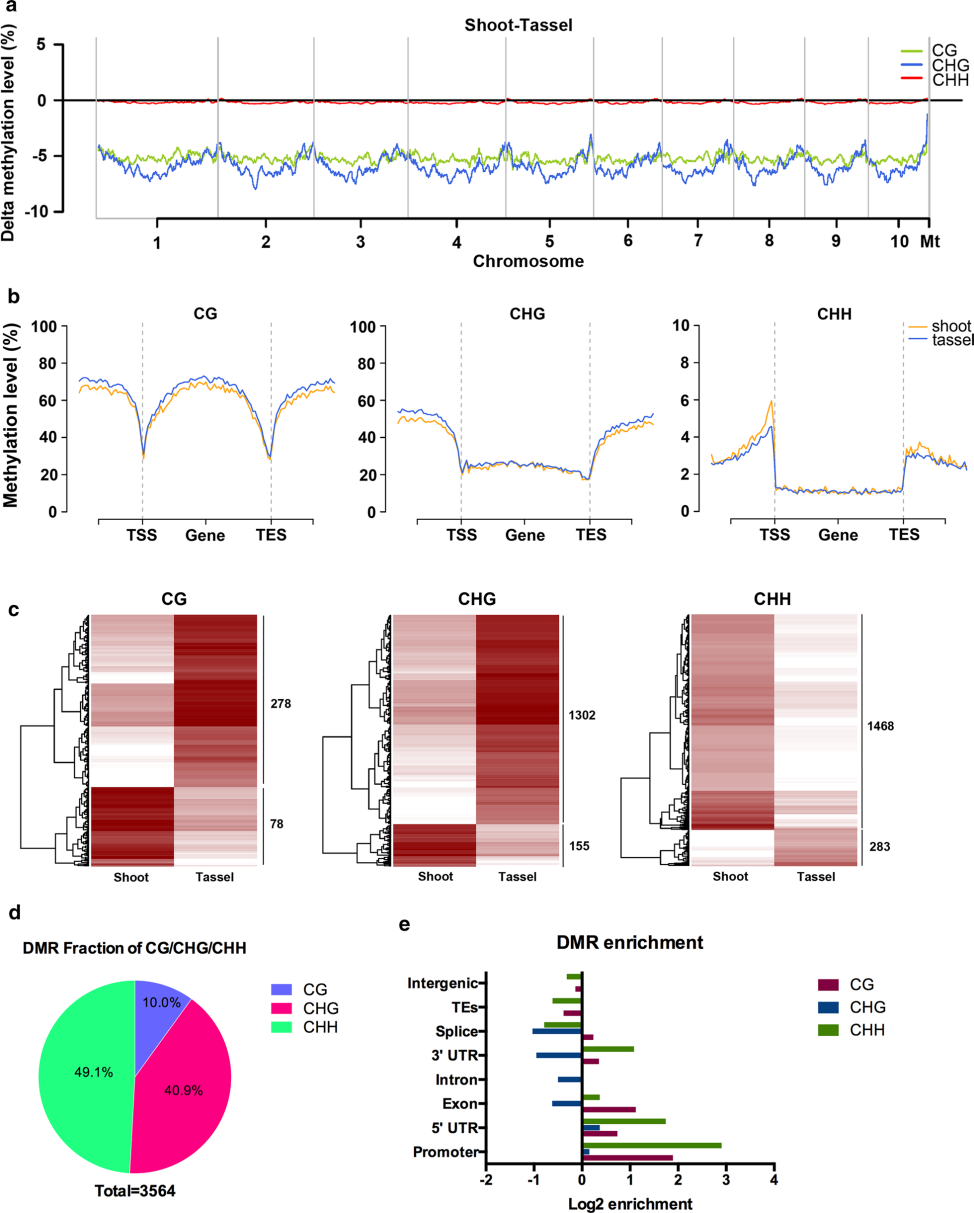
Genome-wide profiling of *Mse*I-RRBS. **a** Genome-wide plot of Δ methylation levels (shoot-tassel primordium). **b** Metagene plot of DNA methylation level for CG/CHG/CHH contexts. **c** Heatmap of DNA methylation level at DMRs between shoots and tassels. **d** Distribution of DMRs in CG/CHG/CHH contexts. **e** Enrichment analysis of DMRs in genomic regions

We aimed to reveal tissue-specific epigenetic regulation between vegetative (shoot) and reproductive tissues (tassel primordium). To avoid confusion and to abridge the manuscript, we have chosen to present only the results of *Mse*I-RRBS in the main text (for the *Cvi*QI results, please see Additional file 4). Overall, we found that tassels exhibited a higher average methylation level than shoots in all CG, CHG, and CHH contexts (Table 2; Additional file 3: Figure S4), which was observed across the genome (Fig. 3a). The distribution of methylation indicates that both CG and CHG methylation are bimodally distributed, and the higher average methylation in tassels is likely due to the increased number of highly methylated CG and CHG sites (Additional file 3: Figure S5). The higher methylation level in the tassel primordium suggests overall *de novo* methylation during vegetative growth and inflorescence organogenesis.

**Table 2:** Average methylation level of CG, CHG, and CHH contexts in four maize RRBS

	Shoot (%)	Tassel (%)	Number of sites
*Mse*I			
CG	77.69	82.84	5,398,944
CHG	61.39	68.08	5,590,339
CHH	1.53	1.80	24,595,783
*Cvi*QI			
CG	64.76	70.75	5,689,945
CHG	53.14	61.01	5,381,124
CHH	1.04	1.77	18,074,911

To investigate DNA methylation in a gene-centric manner, we analyzed the distribution of DNA methylation upstream of TSSs and gene bodies and downstream of TESs. Our analysis indicated that the gene bodies were enriched with CG methylation, but not CHG or CHH methylation (Fig. 3b). The hyper-CG methylation in the middle of gene bodies presumably prevents deleterious insertions of transposons [23]. Increased CHH methylation was observed upstream of TSSs, whereas sharply decreased methylation was found at TSSs (the so-called mCHH islands) (Fig. 3b). Interestingly, we found that, overall, tassels showed greater methylation than shoots at CG and CHG, but less methylation at CHH sites near TSSs and TESs (Fig. 3b). These differential methylation patterns observed in different sequence contexts in the shoot and tassel primordia suggested that different sequence contexts, and especially CHH sites, are subject to differential regulation, which may play an important role during maize development. Regarding TEs, tassels always presented greater methylation than shoots in all three contexts, and TEs presented higher methylation than genes (Additional file 3: Figure S6). For more detailed results regarding TE methylation, please see Additional file 5.

In total, we identified 3564 differentially methylated regions (DMRs) in CG, CHG, or CHH contexts between shoot- and tassel-*Mse*I (Fig. 3c). Interestingly, in contrast to CG and CHG contexts, where more hyper-methylated DMRs were noted in the tassels than in the shoots, more CHH sites exhibited hypo-methylation in the tassel primordium (Fig. 3c).

Among the 3564 DMRs identified from *Mse*I-RRBS (Additional file 6), ~90% were composed of non-CG DMRs (Fig. 3d). Then, we asked where these DMRs were located. We found that both CG DMRs and CHH DMRs exhibited strong enrichment in promoters after adjusting for RRBS fragments (Fig. 3e). In total, 939 genes were associated with DMRs at a promoter or gene body (Additional file 6), constituting differentially methylated genes (DMGs). Among these DMGs, 678 exhibited differential methylation in promoters, 271 in gene bodies and 10 in both regions. We subsequently performed functional annotation for gene ontology (GO) analysis using AgriGO [31] (Additional file 2: Table S4) and found that most of the DMGs were related to the stimulus, reproduction, and response to auxin categories, suggesting that components of these biological processes might be differentially regulated by DNA methylation in shoots and tassels (Additional file 3: Figure S7).

### Transcription and DNA methylation

We performed RNA-seq analysis of the shoot and tassel primordia and identified 3756 (9.7% of all genes) differentially expressed genes (DEGs). Specifically, 2138 (56.9%) of the genes were up-regulated in shoots, and 1618 (43.1%) were down-regulated (Additional file 7). GO analysis indicated that more stimulus response- and photosynthesis-related genes were up-regulated in shoots, reflecting the developmental stage of the coleoptile. As predicted, we found that the tassel primordium was characterized by more reproductive-related genes and DNA replication- and transcription-related genes (Additional file 2: Table S5). Interestingly, DNA methylation-related genes were also up-regulated in tassels, echoing the hyper-methylation found in the tassels.

**Fig. 4:**
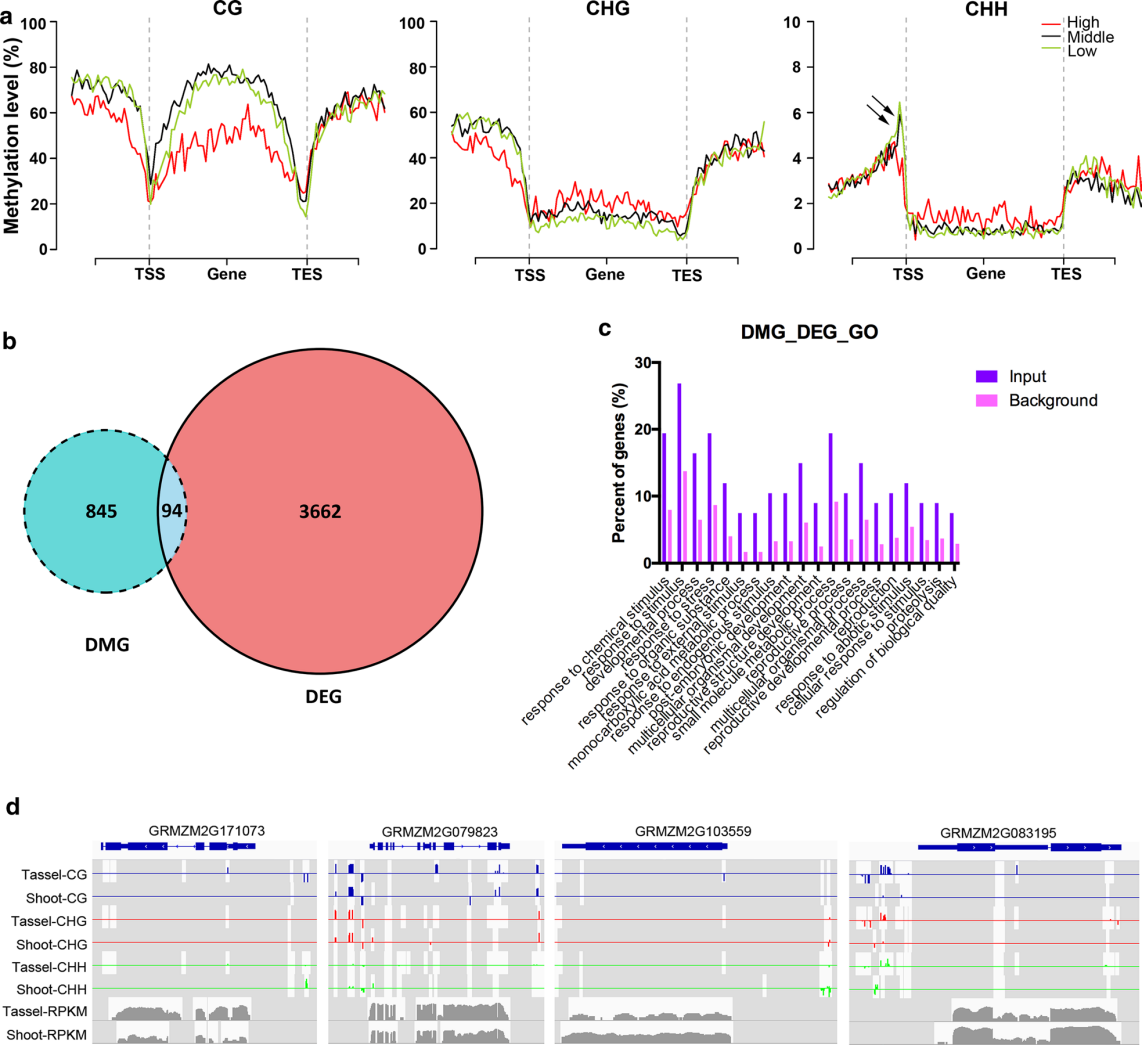
Integrative analysis of DNA methylation and gene expression. **a** Metagene plot of CG (*left*), CHG (*middle*) and CHH (*right*) methylation levels. The *arrows* indicate mCHH islands. *Red* high expression, *black* intermediate expression, *green* low expression. **b** Venn diagram of 94 genes that are differentially methylated and expressed between shoots and tassels, and **c** the functional categories of these genes. **d** IGV snapshots of four genes showing differential methylation and expression with the presence of tissue-specific mCHH islands

Consistent with studies in *Arabidopsis*, rice and maize [3, 6, 32–34], CG and CHG methylation in promoter regions exhibited an inverse correlation with gene expression, whereas genes showing intermediate expression were the most highly methylated at gene bodies (Fig. 4a). Although decreased methylation at both TSS and TES has been observed in plants and animals, we noticed that in maize the reduction for TES was even lower than it was for TSS. Notably, CHH methylation in promoter regions displayed a complex pattern (Fig. 4a, right panel). In the region adjacent to TSSs/TESs, highly expressed genes displayed lower methylation levels, whereas highly expressed genes exhibited higher methylation levels in regions away from TSSs/TESs, suggesting that the correlation between promoter CHH methylation and gene expression may be qualitative rather than quantitative.

To better understand whether differential gene expression may be associated with changes in methylation during maize development, we assessed how many DMGs were differentially expressed between shoots and tassels. Of the 939 DMGs obtained from *Mse*I-RRBS, 94 (Additional file 8) were differentially expressed (Fig. 4b). This result is consistent with a recent finding that DMRs generally exhibit weak associations with quantitative differences in gene expression [35]. Interestingly, GO analysis of these 94 genes suggested significant enrichment in stimulus responses, developmental processes, and reproduction (Table 3; Fig. 4c). Among these 94 genes, rough sheath2-interacting KH-domain gene (GRMZM2G079823), ZOS (GRMZM2G171073), GAPT1 (GRMZM2G083195) and PID (GRMZM2G103559) regulate floral development (Fig. 4d). When we further clustered these 94 genes based on the DNA contexts of their differentially methylated sites, we found that 67 genes (71%) exhibited differential methylation at CHH. This result again suggests that mCHH islands may play an important role in development.

**Table 3:** GO enrichment of overlapping genes of DMGs and DEGs

GO accession	GO term	*p* value
0042221	Response to chemical stimulus	0.0022
0050896	Response to stimulus	0.0034
0032502	Developmental process	0.0037
0006950	Response to stress	0.0046
0010033	Response to organic substance	0.0052
0009605	Response to external stimulus	0.0056
0032787	Monocarboxylic acid metabolic process	0.0058
0009719	Response to endogenous stimulus	0.0065
0009791	Post-embryonic development	0.0065
0007275	Multicellular organismal development	0.0069
0048608	Reproductive structure development	0.0069
0044281	Small molecule metabolic process	0.0074
0022414	Reproductive process	0.01
0032501	Multicellular organismal process	0.011
0003006	Reproductive developmental process	0.012
0000003	Reproduction	0.013
0009628	Response to abiotic stimulus	0.028
0051716	Cellular response to stimulus	0.028
0006508	Proteolysis	0.036
0065008	Regulation of biological quality	0.046

### mCHH islands as a link between DNA methylation and gene expression

**Fig. 5:**
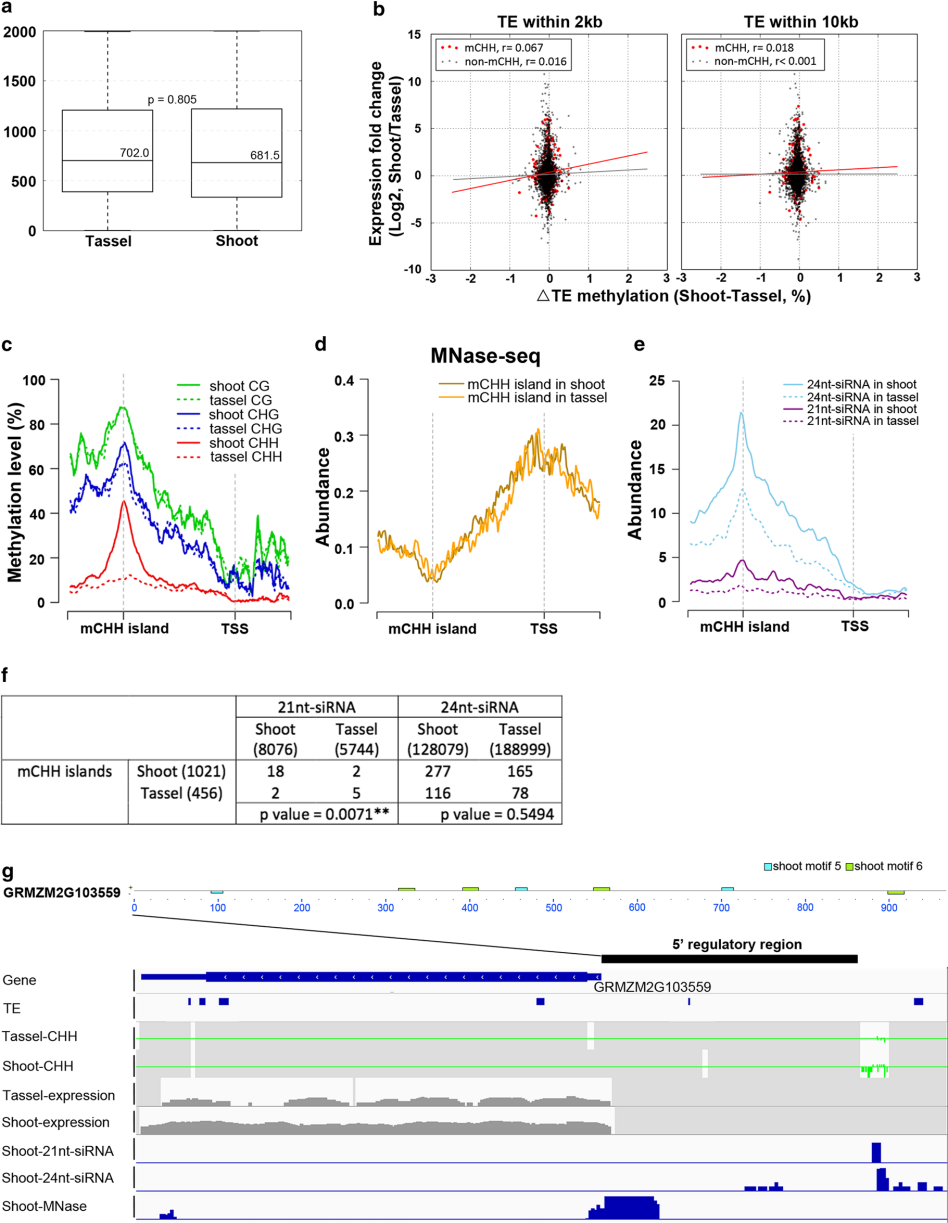
Analysis of tissue-specific mCHH islands. **a** Boxplots of the distances from shoot-specific and tassel-specific mCHH islands to TSS. **b** The correlation between gene expression and nearby TE methylation with or without the presence of mCHH islands. **c** Profiles of DNA methylation levels in shoot and tassel around mCHH islands that are hyper-methylated in shoots. **d** Abundance of MNase-seq reads around mCHH islands and TSS. **e** Abundance of 21nt- and 24nt-siRNAs around mCHH islands in shoots. **f** Correlation analysis of 21nt- and 24nt-siRNA peaks with the distribution of tissue-specific mCHH islands. **g** GRMZM2G103559, harboring an mCHH island in shoots, is more highly expressed in shoots. The mCHH island is located between two nucleosome-occupied regions and both 21nt- and 24nt-siRNAs complementary to mCHH islands are expressed in the shoots

mCHH islands have been implicated in acting as an enforced boundary between heterochromatin and euchromatin in maize [22]. To explore the changes in CHH methylation at promoters during development, we dissected the 2-kb region upstream of TSSs into 100-bp bins and calculated the ΔCHH methylation level between tassel-*Mse*I and shoot-*Mse*I. A histogram of ΔCHH levels in these bins revealed clear peaks at ~25, 32, 40, and 50%, suggesting enrichment of small patches of promoters with ΔmCHH ≥ 25% (Additional file 3: Figure S8). Using a 25% ΔCHH methylation level as a cutoff, we found that 1348 genes showed differentially elevated mCHH islands in the shoots; 807 genes showed differentially elevated mCHH islands in the tassels and 57 genes have mCHH islands in both tissues. The mCHH islands in the tassels (median 13 bp) were shorter than those in the shoots (median 31 bp) ( Additional file 3: Figure S9). The distance from the mCHH islands to TSSs was similar in the two tissues: 702 bp in tassels and 682 bp in shoots (Fig. 5a).

We found that genes with mCHH islands in shoots are more expressed in the shoots, and this is also observed in tassel (Additional file 2: Table S6), suggesting a positive correlation between mCHH islands and gene expression, as shown in previous study [21, 22]. For example, GRMZM2G123308 (Additional file 3: Figure S10), which encodes a MYB-type Golden2-like transcription factor, exhibited a mCHH island at 1200 bp upstream of the promoter in tassels and was up-regulated transcriptionally in tassels. This result suggests that mCHH islands are linked to gene expression in a tissue-specific manner and that CHH islands are associated with gene regulation during plant development.

It was suggested that mCHH islands play a role in protecting neighboring TEs for transcription [22]. We further asked whether TE methylation may be influenced by gene expression level when mCHH islands are present. The ratio of expression level in different tissues is analyzed with delta methylation of neighboring TEs. We observed a weak and positive correlation between TE methylation and gene expression. Interestingly, the correlation is stronger when mCHH islands are present (Fig. 5b), but is weaker when the distance to TEs increases (*p* value = 0.001 of TEs within 2 kb, *p* value = 0.016 of TEs within 10 kb). This result suggested that mCHH islands and the neighboring TEs may form a unique regulatory complex that influences gene expression epigenetically.

To further look into tissue-specific mCHH islands, we profiled CG, CHG, and CHH methylation around mCHH islands and TSS. mCHH islands exhibit differential methylation for shoots and tassels, whereas CG and CHG methylation accumulates similarly in both tissues (Fig. 5c and Additional file 3: Figure S11a). These findings suggest that mCHH islands might be open to methylation-related enzymes. Recently, open chromatin (MNase hypersensitive regions) was shown to localize around active genes and recombination hotspots and to be correlated with DNA hypo-methylation [36]. We therefore asked whether mCHH islands are also associated with open chromatin, and shoot MNase-seq data from cultivar B73 was downloaded and profiled. We found that the nucleosome occupancy was reduced at mCHH islands (Fig. 5d; Additional file 3: Figure S11) from both shoot and tassel tissue, indicating that mCHH islands are generally located in open chromatin.

Small interfering RNAs (siRNAs) of 21nt and 24nt are both involved in RdDM pathways [16], and 24nt-siRNAs have been shown to accumulate on mCHH islands [21]. We profiled siRNA-seq data from shoot (B73) and tassel (A619 background). Indeed, we found that both 24nt- and 21nt-siRNAs are enriched at mCHH islands. Notably, more siRNAs accumulate in these mCHH islands in shoots (Fig. 5e; Additional file 3: Figure S11b). Interestingly, around shoot-specific CHH islands, 24nt-siRNA accumulates similarly in both tissues, whereas 21nt-siRNA only accumulates in shoot, implying that 21nt-siRNA may be co-regulated with CHH islands. Furthermore, when we calculated the number of siRNA peaks located on shoot-specific and tassel-specific mCHH islands, respectively (Fig. 5f). We found that the distribution of 21nt-siRNAs was significantly correlated with tissue-specific mCHH islands, whereas that of 24nt-siRNAs was not. This finding suggests that 21nt-siRNA, but not 24nt-siRNA, plays important roles in directing CHH methylation on tissue-specific mCHH islands in maize.

It has been suggested that mCHH islands in the 5′ regions of genes act as a boundary between euchromatin and heterochromatin [22]. Here, we further showed that tissue-specific mCHH islands are often associated with up-regulated genes during plant development. Many studies have established the importance of transcription factors during floral formation [25, 37]. Therefore, we hypothesized that mCHH islands regulate gene expression in a developmental stage-specific manner by ensuring accessibility to transcription factors in promoter regions. We extracted sequences spanning from mCHH islands to TSSs to represent 5′ mCHH regulatory regions and examined whether they contained specific sequences as well as corresponding transcription factor binding sites (TFBSs) [38]. Additional file 9 presents the predicted motifs of four tassel and six shoot 5′ mCHH regulatory regions. We found that tassel 5′ mCHH regulatory motif 2 was similar to MA1056.1, the binding site of SPL11 in *Arabidopsis* that regulates the timing of the transition from the vegetative to the reproductive phase. Tassel 5′ mCHH regulatory motif 4 was similar to MA0578.1, which is bound by SPL8 to regulate anther development. In accordance with this finding, several maize SPL homologs (such as GRMZM2G160917, GRMZM2G460544, and GRMZM2G307588) indeed are up-regulated in tassels. On the other hand, shoot 5′ mCHH regulatory motif 3 was similar to MA0589.1, the binding motif of ZAP1 that regulates the flower transition. NAC TF NTL9 and AT hook factor AHL20, which regulate stress and defense, are prone to binding shoot 5′ mCHH regulatory motif 6, which is consistent with our differential expression analysis showing that numerous stimulus response genes were differentially regulated. For example, GRMZM2G103559 (PID) exhibited an elevated mCHH island in shoots and was more highly expressed in shoots (Fig. 5g). Our MAST (Motif and Alignment Search Tool [39]) prediction results indicated that the 5′ mCHH regulatory region of GRMZM2G103559 presented three and four matches to shoot 5′ regulatory motifs five and six, respectively. Also in GRMZM2G103559, we found a mCHH island located between two nucleosome-occupied regions, together with high 21nt- and 24nt-siRNA enrichment. These results suggest that mCHH islands are located at open chromatin transition sites, which may ensure accessibility of transcription factors to regulatory sequences downstream of mCHH islands.

## Discussion

Maize is the most highly produced cereal and one of most important crops worldwide. The maize genome is large, abundant in transposons, and highly methylated. The maize WGBS methylome was first generated in 2013 [21, 29]. However, there have been few studies of genome-wide methylation in maize, possibly due to the high sequencing cost given its large genome. Wang et al. [40] performed MeDIP-seq analysis in embryos and endosperm for tissue comparisons and found that most DMRs were located at the shores of CpG islands and they did not affect transcription of the corresponding genes. Li et al. [41] performed low-coverage WGBS together with high-coverage sequence-capture bisulfite sequencing in methylation-related mutants to establish DNA methylation pathways in maize and found that severe perturbations of the maize methylome may have stronger deleterious phenotypic effects than in *Arabidopsis*. These findings indicate that DNA methylation is very likely a strong epigenetic factor in maize and underscore the need for genome-wide, high-resolution, low-cost methods to study the maize methylome.

Other experimental approaches have been adapted to probe methylation differences in crops with large genome. Chwialkowska et al. [42] developed methylation-sensitive amplification polymorphism sequencing (MSAP-seq) to identify differentially methylated cytosines between roots and leaves in barley. MSAP-seq uses methylation-sensitive restriction enzyme *Hpa*II to recognize CCGG sites and digest genomes. After sequencing, differentially methylated cytosines are predicted by comparing normalized read counts. MSAP-seq is a cost-effective method to measure cytosine methylation, but it has a limitation that it majorly probes CG sites and CHG sites in minor due to the enzyme specificity. Furthermore, it is not single-base resolution and cannot distinguish the methylation level between CG, CHG, and CHH contexts. It estimates the relative methylation level with relative read counts rather than the digital measurement.

In the present study, we developed our own enzyme selection pipeline for maize RRBS (Fig. 1). Based on in silico digestion and enrichment analysis, we selected *Mse*I and *Cvi*QI to generate promoter- and gene body-enriched reduced representation genomes, respectively (Fig. 2b). We constructed RRBS libraries from the shoot and tassel primordia of the maize B73 inbred line. First, we confirmed that the results of our shoot-*Mse*I-RRBS procedure were similar to previously published WGBS data. Our RRBS libraries covered 82–115 M cytosines (Additional file 2: Table S3), which is a much higher number than for other targeted methylation arrays, such as Roche NimbleGen arrays that cover 270 k, 1.4 M, or 2.1 M custom probes [30]. Then, we demonstrated that *Mse*I-RRBS showed promoter enrichment (Fig. 2c). Taken together, these results demonstrate that our maize RRBS is both effective and feasible. Comparing to other approaches, maize RRBS costs less than WGBS while preserving the property of high resolution and wide coverage that MeDIP-seq, MSAP-seq, and NimbleGen array could not accomplish.

Overall, tassels exhibited greater methylation than shoots (Fig. 3a), suggesting accumulation of methylation over the course of maize development and that high methylation might be a protective mechanism for reproductive tissue. Previous studies have demonstrated that mCHH islands are relatively stable across different tissues [22]. In the present study, 3564 DMRs were found between shoots and tassels. The shoots had more hypo-methylated CG and CHG DMRs, but more hyper-methylated CHH DMRs (Fig. 3c), suggesting a possible tissue-specific regulatory mechanism that is potentially directed by CHH methylation. Metagene plots further indicated greater methylation of CHH in promoter regions in shoots than in tassels (Fig. 3b). In addition, ~90% of DMRs were located in a non-CG context (Fig. 3d). Interestingly, CHH DMRs exhibited significant enrichment in promoter regions, whereas CHG DMRs did not, indicating possible branching regulatory systems in non-CG methylation. In total, we identified 939 DMGs, most of which were related to developmental and reproductive processes based on GO analysis, suggesting that changes in DNA methylation are correlated with developmental stages.

To assess the relationship between DNA methylation and transcription, we also generated shoot and tassel RNA-seq data. In total, we identified 3756 DEGs. Specifically, 2138 of the DEGs were up-regulated in shoots, and 1618 were up-regulated in tassels. Only 94 genes overlapped between the DMGs and DEGs (Fig. 4b), suggesting a limited correlation between DNA methylation and transcription when the methylation of all CG, CHG, and CHH sites was considered. However, these 94 genes exhibited enrichment in the categories of reproduction and stimulus response cellular processes in our GO analysis, suggesting that the major players in vegetative and reproductive growth may be regulated by DNA methylation (Fig. 4c).

mCHH islands were first reported in a recently published maize WGBS study [22]. These highly methylated CHH regions are likely enriched at TE edges close to highly expressed genes. Based on comparative analysis of tassel and shoot RRBS, we have further characterized tissue-specific mCHH islands as being associated with up-regulated genes with statistical significance. Furthermore, we also observed that gene expression is more correlated with nearby TE methylation in the presence of mCHH islands (Fig. 5b). We incorporated MNase-seq data and found that mCHH islands are located at open chromatin transition zones (Fig. 5d). This finding suggested that chromosome accessibility may relate functionally to the high CHH methylation level at mCHH islands. Furthermore, we found that both 21nt- and 24nt-siRNAs are enriched in mCHH islands (Fig. 5e, f). This enrichment is more significant at the shoot stage than at the tassel stage, indicating that small RNA epigenetic regulation may be more active at the vegetative stage to induce the development of highly divergent cell types. Lastly, we found that only 21nt-siRNAs exhibit tissue-specific regulation on mCHH islands (Fig. 5f).

To verify the extent to which mCHH islands may regulate gene expression, we used 5′ mCHH regulatory regions as an input to predict sequence motifs. Our results indicated that these 5′ regulatory motifs show sequence similarity to known motifs that regulate the vegetative-to-reproductive phase transition, the flowering process and stimulus responses (Additional file 9). Thus, in addition to acting as a boundary between heterochromatin and euchromatin, mCHH islands may also indicate specific genes that need to be modulated, strengthening the indirect link between DNA methylation and gene expression.

In parallel with *Mse*I-RRBS, we performed gene body-enriched RRBS using *Cvi*QI. Based on our data, enrichment for gene bodies in *Cvi*QI-RRBS was observed. We found that only ~1.9% of cytosine sites were shared between *Mse*I-RRBS and *Cvi*QI-RRBS, suggesting divergence and complementation of the two reduced representation genomes (Additional file 2: Table S3). No single-cell methylome has yet been reported for maize, which could reveal heterogeneity in results obtained from pooling cells. Nevertheless, compared with normal WGBS, in low-input single-cell WGBS, some information will necessarily be lost during library construction. Single-cell RRBS therefore is an alternative approach for preserving genome information, given that it only involves sequencing of targeted regions, and therefore, it is a better method for revealing cell heterogeneity.

## Conclusions

Our maize RRBS approach is the first application of ROI-directed RRBS in a plant or crop species. Using maize RRBS to reduce sequencing costs makes sample comparisons more feasible. Here, we compared tassel primordium and shoots to reveal the role of DNA methylation during the vegetative-to-reproductive transition. We successfully identified genes related to reproduction and defense that are differentially methylated and expressed. Additionally, we found that expression of genes with tissue-specific mCHH islands tends to be positively correlated with methylation levels. Based on integrative analysis using MNase-seq and siRNA-seq data, we reveal that mCHH islands accumulate 21nt-siRNAs in a tissue-specific manner and that mCHH islands mark the transition zone to open chromatin in each tissue type for the exposure of potential TFs.

## Materials and methods

### Plant material

Maize inbred line B73 was used in this study. For shoot samples, seeds were germinated on wet paper towels in an incubator at 25 °C. After 5 days, shoots at the coleoptilar stage were excised and stored at −80 °C. For tassel primordium, tassel primordia of approx. 5 mm length were collected from B73 plants at V5–V6 stages and stored at −80 °C.

### Nucleus isolation and nuclear DNA extraction

Nuclear DNA was prepared from nuclei as described in Peterson et al. [43]. In brief, frozen tissues around 1 g were ground with mortar and pestle in liquid nitrogen and homogenized in the extraction buffer with 0.5% Triton X-100. After filtering and washing, crude extracts were pelleted at 1200 g for 20 min and suspended in the nuclear buffer. Finally, nuclei were isolated by centrifuging through 30% Percoll at 650 g for 60 min and suspended in the nuclear buffer. To extract nuclear DNA, SDS was added to make a final concentration of 2% and heated at 60 °C for 10 min, and then, DNA was purified and precipitated after RNase A treatment.

### Maize RRBS library construction and sequencing

One microgram genomic DNA was digested with *Mse*I overnight at 37 °C and cleaned with AMPure XP beads. The DNA ends were repaired and A’ tailed with Klenow exo (Thermo Fisher Scientific), followed by ligation with pre-methylated Illumina adaptors. The ligation products were size-selected with Ampure XP beads and purified for subsequent bisulfite conversion (Qiagen EpiTect Fast). Bisulfite-converted DNA was amplified with Pfu Turbo Cx (Agilent Technologies, Santa Clara, CA). The final DNA library concentration was quantified using a BioAnalyzer (Agilent Technologies, Santa Clara, CA) and qPCR, then diluted to 10 nM and loaded onto a flow cell for cluster generation. The libraries were sequenced on an Illumina HiSeq 2000 platform using paired-end 100 cycle mode.

### Measuring the methylation level per cytosine and bulk methylation levels

The bisulfite-converted reads were aligned to the maize reference genome AGPv3 using the bisulfite aligner BS Seeker 2 [27]. To generate genome-wide DNA methylation profiles, we calculated the methylation level for each cytosine covered in the genome. Given that bisulfite treatment converts unmethylated cytosines (Cs) to thymines (Ts), we estimated the methylation level at each cytosine as #C/(#C + #T), where #C is the number of methylated reads, and #T is the number of unmethylated reads [44]. The methylation level per cytosine serves as an estimate of the percentage of cells that are methylated at this cytosine. We only included cytosines that were covered by at least four reads. The bulk methylation level per methylome is the average methylation level of all cytosines.

### Identifying differentially methylated regions (DMR)

Regions of the genome showing significantly different methylation levels were identified and defined as DMRs. Genes adjacent to these DMRs were considered differentially methylated genes. To quantify the difference between the groups at a given site, a Student’s *t* test was performed at each CG site. The larger the generated *t* scores, the greater the difference in methylation levels for that pairwise comparison. To obtain an accurate measurement of these differences after the sites were combined into fragments, the *t* scores of all sites within that fragment were averaged to produce a *z* score. To qualify as a DMR, the fragment had to: (1) exhibit a difference of ≥10% in the mean methylation level between the two groups being compared; (2) exhibit at least three cytosines for which methylation levels were observed in all relevant samples; and (3) present a *z* score below a threshold relevant to that comparison. The selection of the *z* score threshold was based on the false discovery rate, which was estimated by comparing the real data to simulated methylomes as the control for false discovery rate (FDR) computation.

### False discovery rate (FDR) estimation

Simulated methylomes showing the same read coverage per site as real samples were constructed to assess the false discovery rate (FDR) of the DMRs. For each CG site in each simulated sample, reads were simulated based on the average methylation level (Pm) from all real samples at that CG site. This simulation of reads was repeated for all samples throughout the genome. The number of methylated reads (Cs) at a site of coverage, n, represented a random sample from the binomial distribution, B (n, Pm). Given that the reads were simulated from a binomial distribution showing the same average methylation levels as the real samples, the differences in methylation patterns across genes, repeats, and promoters were preserved. The simulated data exhibited the same coverage as the real samples so statistical power was not affected. The simulated methylomes should present no difference in methylation levels between the two groups being compared (i.e., no DMRs), given that they were all selected using the same methylation frequency. Any DMRs (or DMR-associated genes) identified from these simulated samples were therefore considered false positives. For each comparison, the entire process was repeated to detect DMRs in simulated samples. We first performed *t* tests on individual sites and then summarized the *t* scores per fragment with a *z* score. For each *z* score threshold, we computed the number of DMRs identified in the simulated data compared with that found in the real data. We used the ratio between these two values to compute the FDR. We chose a *z* score threshold that resulted in a false discovery rate of less than 10% in all comparisons.

### RNA-seq and data processing

RNA-seq was performed following standard Illumina protocols. Total RNAs were treated with DNaseI (Roche Applied Science), cleaned with phenol–chloroform, and precipitated with ethanol. Libraries were generated with TruSeq RNA Library Prep Kit and sequenced on HiSeq 2000 following the manufacturer’s instructions (Illumina, La Jolla, CA). The resulting libraries were then subjected to PCR amplification, prior to sequencing on Illumina HiSeq 2000 sequencers at the National Center for Genome Medicine at Academia Sinica. To quantify gene expression levels, we mapped the reads onto the maize genome AGPv3 using the alignment tool TopHat2 [45]. Reads per kilobase per million mapped reads (RPKM) values were computed using cuffdiff [46]. For each gene, a statistical comparison of RPKM values between tassel and shoot samples was made via the *Z* test of Kal et al. [47]. Statistical significance was determined as p < 0.05, and there was at least a twofold change in the expression of tassels relative to shoots.

### MNase-seq data processing

Shoot MNase-seq data was downloaded from NCBI short read archive (SRP064243) [36]. After trimming of adaptor sequences using Cutadapt [48], paired-end reads were mapped to the maize B73 AGPv3 reference genome, using Bowtie2 [49] with options “no-mixed,” “no-discordant,” “no-unal,” and “dovetail” for each replicate digest and for the genomic DNA. Metaplot matrix was generated with deepTools [50] and plot with R.

### Small RNA sequencing data processing

B73 Shoot siRNA data was downloaded from Gene Expression Omnibus (GEO) with accession number GSE39232 . Tassel siRNA data was downloaded with accession number GSE52879 (wild type in A619 background). Adaptors were trimmed with Cutadapt. Reads with length 21nt and 24nt were selected and mapped to maize B73 AGPv3 reference genome with Bowtie2, respectively. Metaplot matrix was generated with deepTools and plot with R.

## Additional files

**Additional file 1.** 85 restriction enzymes tested in this study.

**Additional file 2: Table S1.** Determination of size selection boundaries after MseI in silico digestion. **Table S2**. Expected cost of maize RRBS. **Table S3**. Numbers of covered cytosines in maize RRBS libraries (% of total cytosines of the genome). **Table S4**. Top 10 enriched GO accessions of DMGs (MseI-RRBS). **Table S5**. GO enrichment of DEGs. Table S6. Correlation of gene expression and mCHH island.

**Additional file 3: Figure S1.** Photos of maize tissues in this study. (**a**) Shoot, scale bar is 1cm. (**b**) Tassel primordium, scale bar is 1mm. **Figure S2**. Base composition of maize RRBS reads. **Figure S3**. Box plot of common sites methylation level in RRBS and WGBS. **Figure S4**. Average methylation level of maize RRBS. **Figure S5**. Fraction of CG/CHG/CHH methylation level in Tassel-MseI and Shoot-MseI. **Figure S6**. Metagene plots of CG, CHG CHH methylation on TE in Shoot- and Tassel-MseI RRBS.** Figure S7**. GO analysis of DMGs between shoot- and tassel-MseI.** Figure S8**. Histogram of CHH methylation level of 100bp bins 2kb upstream of TSS between tassel-MseI and shoot-MseI. **Figure S9**. Size of mCHH islands in tassel and shoot. **Figure S10**. An example of a gene GRMZM2G123308 with mCHH island in tassel is up-regulated in tassel. **Figure S11**. Metaplots of 5’ regulatory regions between tassel mCHH islands and TSSs with CHH methylation and siRNA data. (**a**) Profiles of DNA methylation levels in shoot and tassel around tassel mCHH islands that are hypermethylated in tassel. (**b**) Abundance of 21nt- and s4nt-siRNA around mCHH islands that are hypermethylated in tassel.

**Additional file 4.** Results of *Cvi*QI-RRBS.

**Additional file 5.** Results of TE methylation profiling.

**Additional file 6.** DMR information.

**Additional file 7.** RNA-seq information.

**Additional file 8.** List of overlapping DMGs and DEGs.

**Additional file 9.** Motifs and predicted TF binding of 5′ mCHH regulatory regions.

## Abbreviations

DEG: differentially expressed gene
DMG: differentially methylated gene
DMR: differentially methylated region
FDR: false discovery rate
GO: gene ontology
RPKM: reads per kilobase per million mapped reads
RdDM: RNA-dependent DNA methylation
ROI: region of interest
RRBS: reduced representation bisulfite sequencing
TE: transposable element
TES: transcription end site
TF: transcription factor
TFBS: transcription factor binding site
TSS: transcription start site
WGBS: whole-genome bisulfite sequencing
