# Supplementary material for: Optimized reduced representation bisulfite sequencing reveals tissue-specific mCHH islands in maize
Source: Epigenetics Chromatin. 2017 Aug 30;10:42. doi: 10.1186/s13072-017-0148-y (PMC5577757; doi:10.1186/s13072-017-0148-y)
Supplement: Supplementary file 1 — Additional file 1. 85 restriction enzymes tested in this study. [file 13072_2017_148_MOESM1_ESM.docx]

**Additional file 1.** 85 restriction enzymes tested in this study

| Enzyme | Cutting site |
| --- | --- |
| AatII | GATGTC |
| Acc65I | GGTACC |
| AciI | CCGC |
| AclI | AACGTT |
| AfeI | AGCGCT |
| AflII | CTTAAG |
| AgeI | ACCGGT |
| AluI | AGCT |
| ApaI | GGGCCC |
| ApaLI | GTGCAC |
| AseI | ATTAAT |
| AvrII | CCTAGG |
| BamHI | GGATCC |
| BclI | TGATCA |
| BfaI | CTAG |
| BfuCI | GATC |
| BglII | AGATCT |
| BmtI | GCTAGC |
| BsiWI | CGTACG |
| BspDI | ATCGAT |
| BspEI | TCCGGA |
| BspHI | TCATGA |
| BsrGI | TGTACA |
| BssHII | GCGCGC |
| BstBI | TTCGAA |
| BstUI | CGCG |
| BstZ17I | GTATAC |
| ClaI | ATCGAT |
| CviAII | CATG |
| CviQI | GTAC |
| DpnI | GATC |
| DraI | TTTAAA |
| EagI | CGGCCG |
| EcoRI | GAATTC |
| EcoRV | GATATC |
| FatI | CATG |
| FspI | TGCGCA |
| HaeIII/PhoI | GGCC |
| HhaI | GCGC |
| HindIII | AAGCTT |
| HinP1I | GCGC |
| HpaI | GTTAAC |
| HpyCH4IV | ACGT |
| HpyCH4V | TGCA |
| KasI | GGCGCC |
| KpnI | GGTACC |
| MboI | GATC |
| MfeI | CAATTG |
| MluI | ACGCGT |
| MnII | CCTC |
| MscI | TGGCCA |
| MseI | TTAA |
| MspI/HpaI | CCGG |
| NaeI | GCCGGC |
| NarI | GGCGCC |
| NcoI | CCATGG |
| NdeI | CATATG |
| NgoMIV | GCCGGC |
| NheI | GCTAGC |
| NlaIII | CATG |
| NruI | TCGCGA |
| NsiI | ATGCAT |
| PciI | ACATGT |
| PmlI | CACGTG |
| PsiI | TTATAA |
| PspOMI | GGGCCC |
| PstI | CTGCAG |
| PvuI | CGATCG |
| PvuII | CAGCTG |
| RsaI | GTAC |
| SacI | GAGCTC |
| SacII | CCGCGG |
| SalI | GTCGAC |
| ScaI | AGTACT |
| SfoI | GGCGCC |
| SmaI | CCCGGG |
| SnaBI | TACGTA |
| SpeI | ACTAGT |
| SphI | GCATGC |
| SspI | AATATT |
| StuI | AGGCCT |
| TaqaI | TCGA |
| XbaI | TCTAGA |
| XhoI | CTCGAG |
| XmaI | CCCGGG |

**Reference**

New England BioLabs. 2016. https://www.neb.com/
