# Supplementary material for: Optimized reduced representation bisulfite sequencing reveals tissue-specific mCHH islands in maize
Source: Epigenetics Chromatin. 2017 Aug 30;10:42. doi: 10.1186/s13072-017-0148-y (PMC5577757; doi:10.1186/s13072-017-0148-y)
Supplement: Supplementary file 2 — Additional file 2: Table S1. Determination of size selection boundaries after MseI in silico digestion. Table S2. Expected cost of maize RRBS. Table S3. Numbers of covered cytosines in maize RRBS libraries (% of total cytosines of the genome). Table S4. Top 10 enriched GO accessions of DMGs (MseI-RRBS). Table S5. GO enrichment of DEGs. Table S6. Correlation of gene expression and mCHH island. [file 13072_2017_148_MOESM2_ESM.docx]

**Additional file 2**

Containing Table S1-S6

**Table S1** Determination of size selection boundaries after *Mse*I *in silico* digestion

| Boundary | Fragment size | Covered genome (%) | Covered promoter (%) | Promoter enrichment (Log2) |
| --- | --- | --- | --- | --- |
| Lower | 40-50 | 0.9 | 1.4 | 0.64 |
|  | 50-60 | 0.9 | 1.8 | 1.00 |
|  | 60-70 | 1.1 | 2.0 | 0.86 |
|  | 70-80 | 1.0 | 2.3 | 1.20 |
|  | 80-90 | 1.1 | 2.5 | 1.18 |
|  | 90-100 | 1.0 | 2.6 | 1.38 |
| Upper | 250-260 | 0.9 | 1.3 | 0.53 |
|  | 260-270 | 0.9 | 1.2 | 0.41 |
|  | 270-280 | 0.9 | 1.0 | 0.15 |
|  | 280-290 | 0.9 | 0.9 | 0.00 |
|  | 290-300 | 0.8 | 0.9 | 0.18 |
|  | 300-310 | 0.8 | 0.6 | -0.42 |

**Table S2** Expected cost of maize RRBS

|  | Genome size | Number of PE reads to cover 10X |
| --- | --- | --- |
| WGBS | 2,060,653,601 | 103,032,680 |
| *Mse*I-RRBS | 565,511,128 | 28,275,556 |
| *Cvi*QI-RRBS | 269,964,479 | 13,498,223 |

**Table S3** Numbers of covered cytosines in maize RRBS libraries (% of total cytosines of the genome)

| Dataset | CG | CHG | CHH | Total | Overlapped sites |
| --- | --- | --- | --- | --- | --- |
| Tassel-*Mse*I | 16,306,072 (9.08%) | 16,351,838 (10.36%) | 75,611,491 (12.15%) | 108,269,410 (11.28%) | 15,019,277 (1.56%) |
| Tassel-*Cvi*QI | 16,258,671 (9.05%) | 15,010,333 (9.51%) | 51,138,778 (8.21%) | 82,407,782 (8.58%) |  |
| Shoot-*Mse*I | 13,942,762 (7.76%) | 14,053,187 (8.90%) | 64,968,297 (10.44%) | 92,964,246 (9.68%) | 18,070,389 (1.88%) |
| Shoot-*Cvi*QI | 21,651,331 (12.06%) | 20,048,628 (12.70%) | 73,652,887 (11.83%) | 115,352,846 (12.02%) |  |

**Table S4** Top 10 enriched GO accessions of DMGs (*Mse*I-RRBS)

| GO accession | GO term | *p* value |
| --- | --- | --- |
| 0042221 | Response to chemical stimulus | <0.001 |
| 0009753 | Response to jasmonic acid stimulus | <0.001 |
| 0009719 | Response to endogenous stimulus | <0.001 |
| 0050896 | Response to stimulus | <0.001 |
| 0010154 | Fruit development | 0.001 |
| 0000003 | Reproduction | 0.002 |
| 0022414 | Reproductive process | 0.002 |
| 0006950 | Response to stress | 0.002 |
| 0009733 | Response to auxin stimulus | 0.002 |

**Table S5** GO enrichment of DEGs

| Group | GO accession | GO term | *p* value |
| --- | --- | --- | --- |
| Shoot up-regulated | 0050896 | Response to stimulus | 1.60E-31 |
|  | 0009628 | Response to abiotic stimulus | 1.00E-29 |
|  | 0042221 | Response to chemical stimulus | 2.60E-26 |
|  | 0006950 | Response to stress | 2.00E-23 |
|  | 0015979 | Photosynthesis | 5.10E-05 |
| Tassel up-regulated | 0006281 | DNA repair | 3.40E-11 |
|  | 0007049 | Cell cycle | 9.20E-10 |
|  | 0006350 | Transcription | 1.60E-09 |
|  | 0006260 | DNA replication | 1.90E-09 |
|  | 0006306 | DNA methylation | 4.10E-07 |
|  | 0000003 | Reproduction | 5.40E-06 |

**Table S6** Correlation of gene expression and mCHH island

|  |  | Gene expression | |
| --- | --- | --- | --- |
|  |  | Shoot > Tassel | Tassel > Shoot |
| mCHH island | Shoot | 159 | 174 |
|  | Tassel | 131 | 109 |

*p-*value = 0.0012
