# Supplementary material for: Optimized reduced representation bisulfite sequencing reveals tissue-specific mCHH islands in maize
Source: Epigenetics Chromatin. 2017 Aug 30;10:42. doi: 10.1186/s13072-017-0148-y (PMC5577757; doi:10.1186/s13072-017-0148-y)
Supplement: Supplementary file 3 — Additional file 3: Figure S1. Photos of maize tissues in this study. (a) Shoot, scale bar is 1cm. (b) Tassel primordium, scale bar is 1mm. Figure S2. Base composition of maize RRBS reads. Figure S3. Box plot of common sites methylation level in RRBS and WGBS. Figure S4. Average methylation level of maize RRBS. Figure S5. Fraction of CG/CHG/CHH methylation level in Tassel-MseI and Shoot-MseI. Figure S6. Metagene plots of CG, CHG CHH methylation on TE in Shoot- and Tassel-MseI RRBS. Figure S7. GO analysis of DMGs between shoot- and tassel-MseI. Figure S8. Histogram of CHH methylation level of 100bp bins 2kb upstream of TSS between tassel-MseI and shoot-MseI. Figure S9. Size of mCHH islands in tassel and shoot. Figure S10. An example of a gene GRMZM2G123308 with mCHH island in tassel is up-regulated in tassel. Figure S11. Metaplots of 5’ regulatory regions between tassel mCHH islands and TSSs with CHH methylation and siRNA data. (a) Profiles of DNA methylation levels in shoot and tassel around tassel mCHH islands that are hypermethylated in tassel. (b) Abundance of 21nt- and s4nt-siRNA around mCHH islands that are hypermethylated in tassel. [file 13072_2017_148_MOESM3_ESM.docx]

**Additional file 3**

Containing Figure S1-S11

**Figure S1**


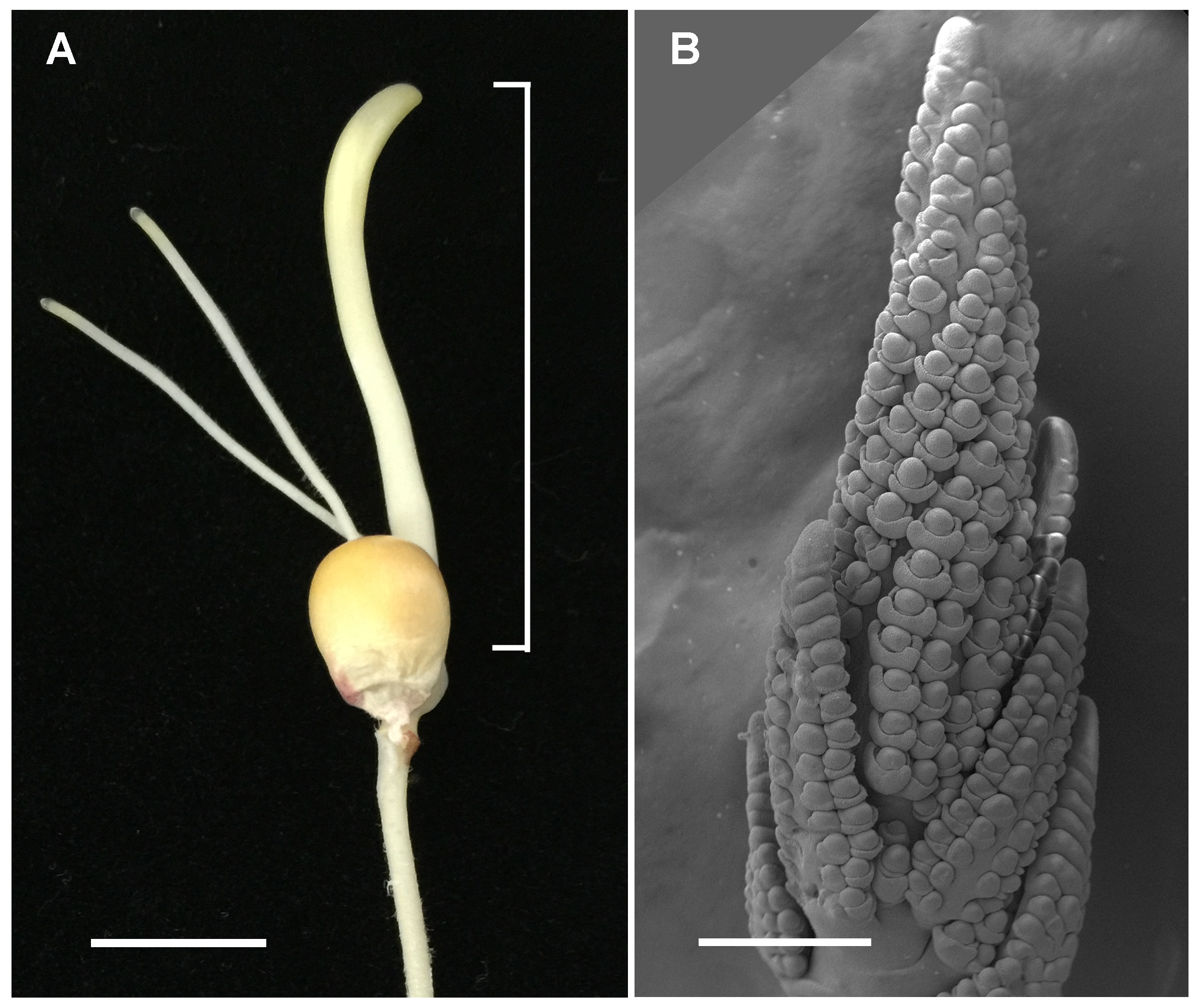


**Figure S1.** Photos of maize tissues in this study. (**a**) Shoot, scale bar is 1cm. (**b**) Tassel primordium, scale bar is 1mm.

**Figure S2**


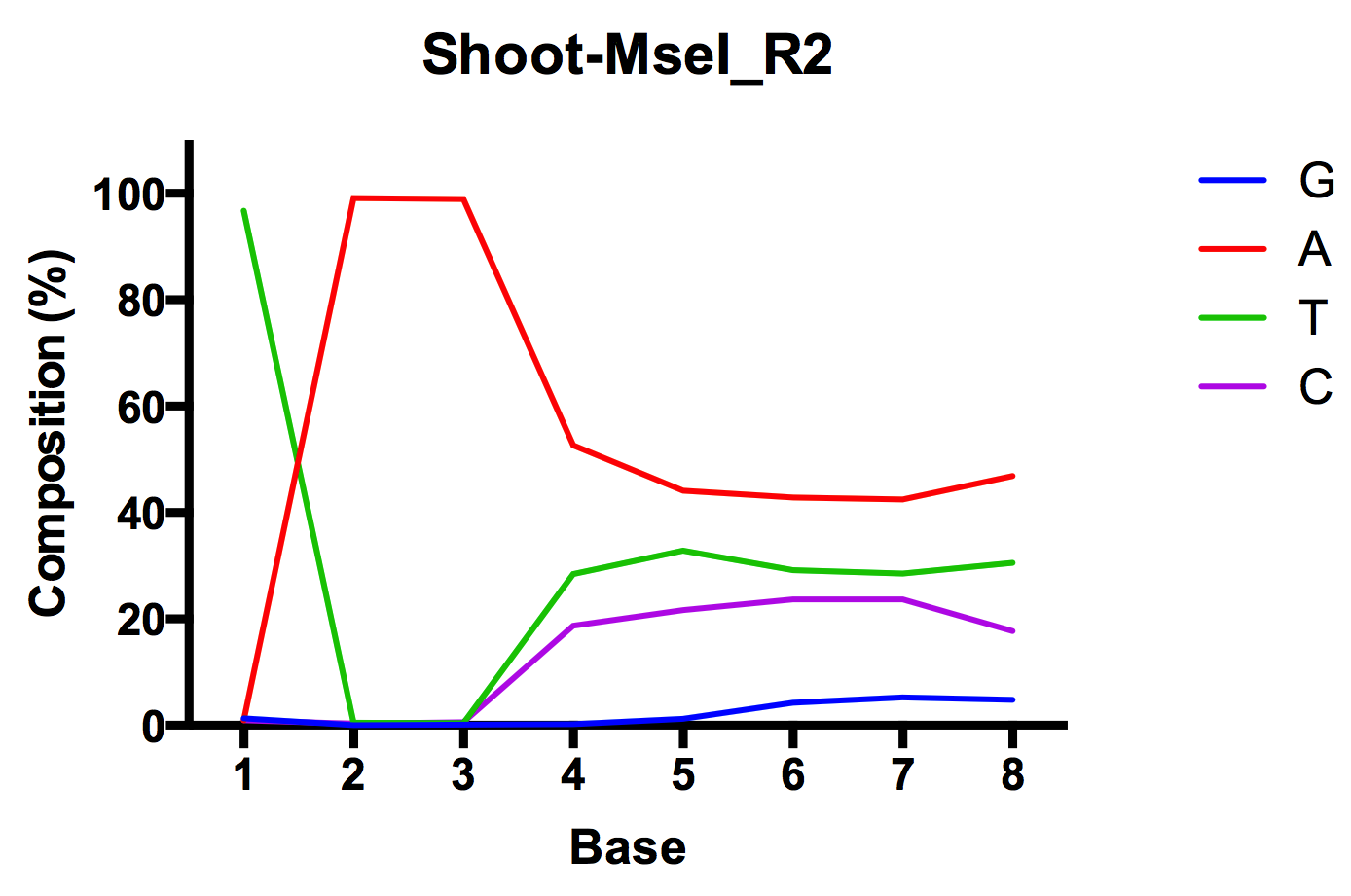

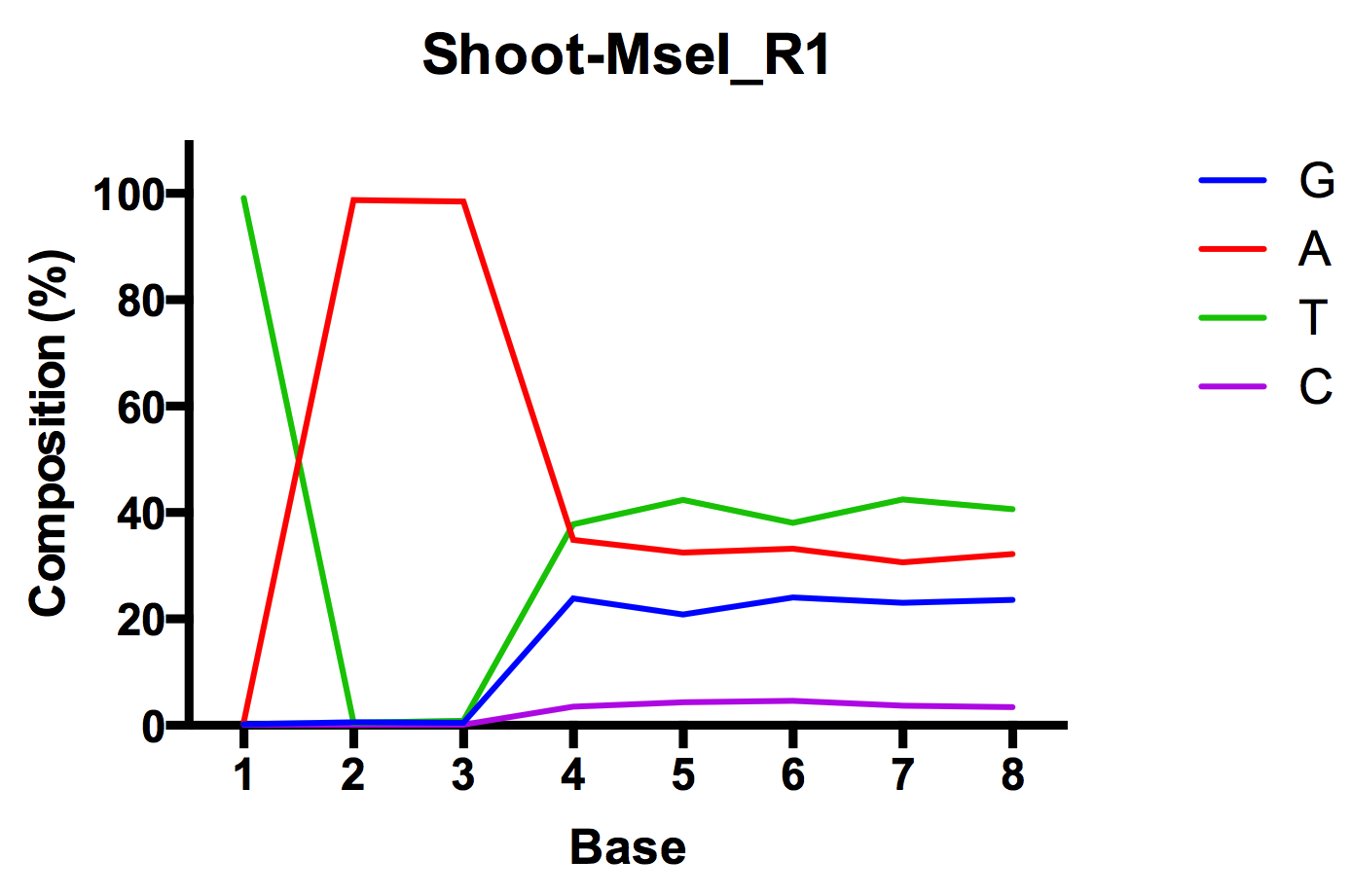


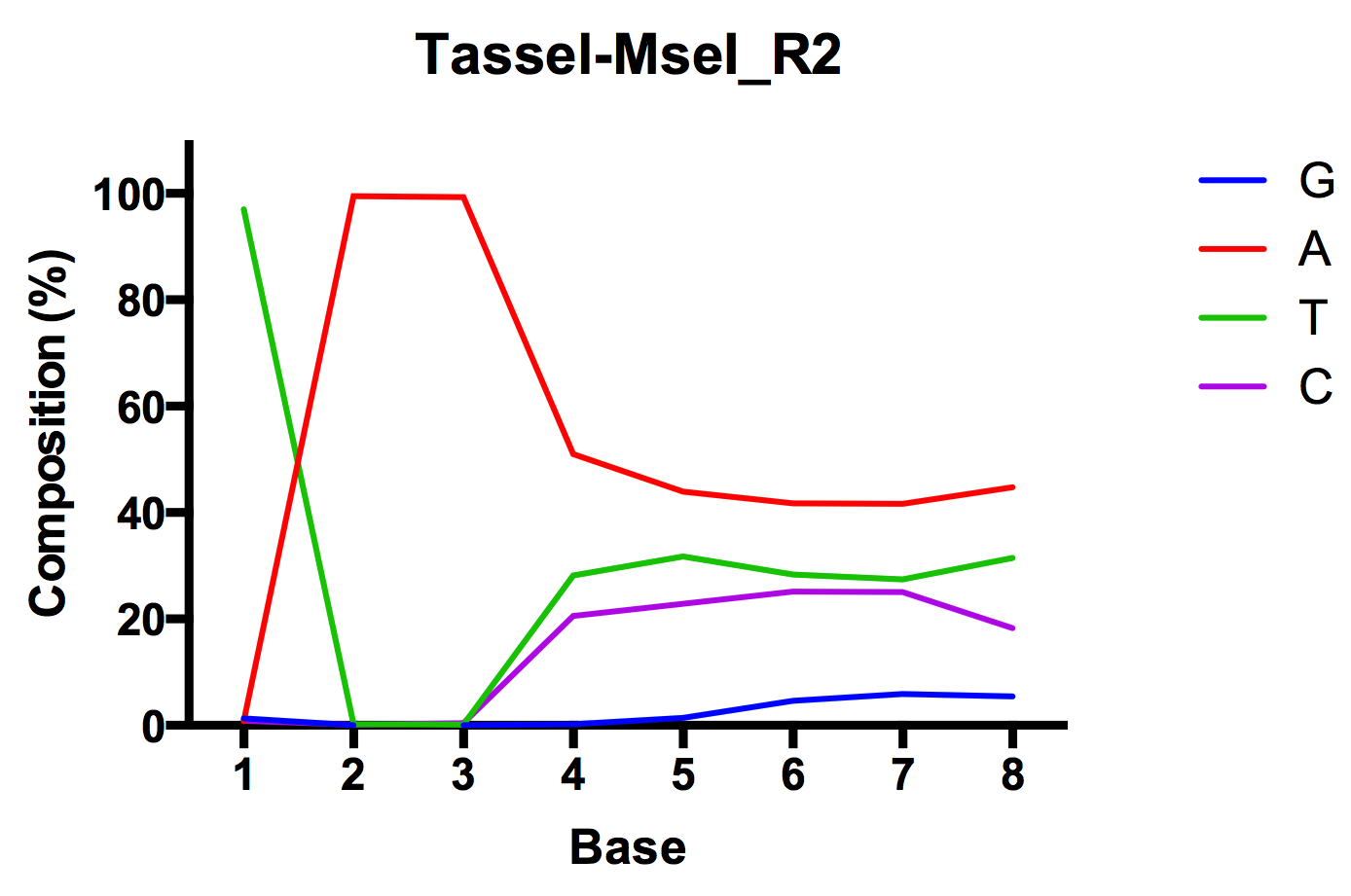

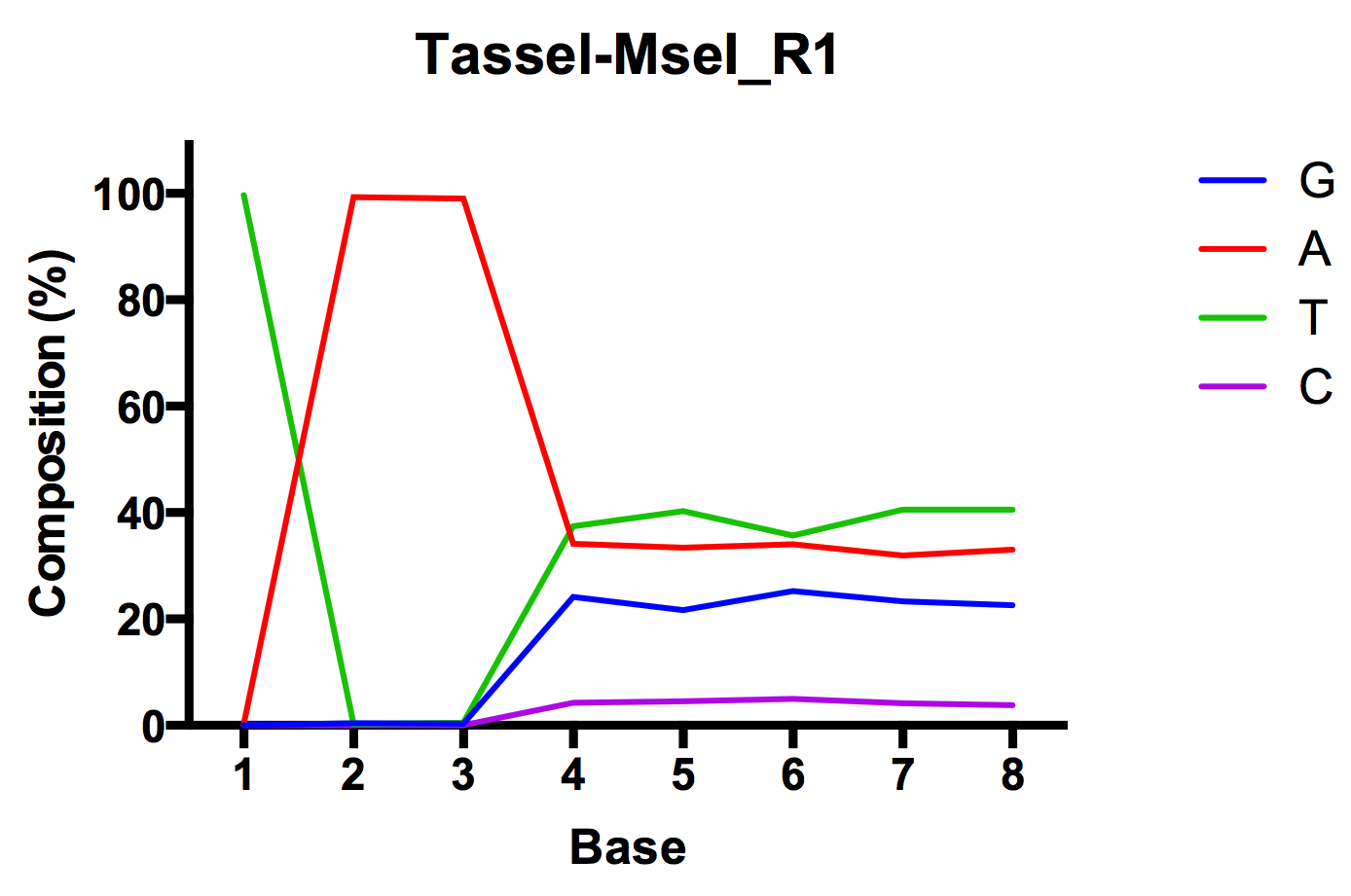


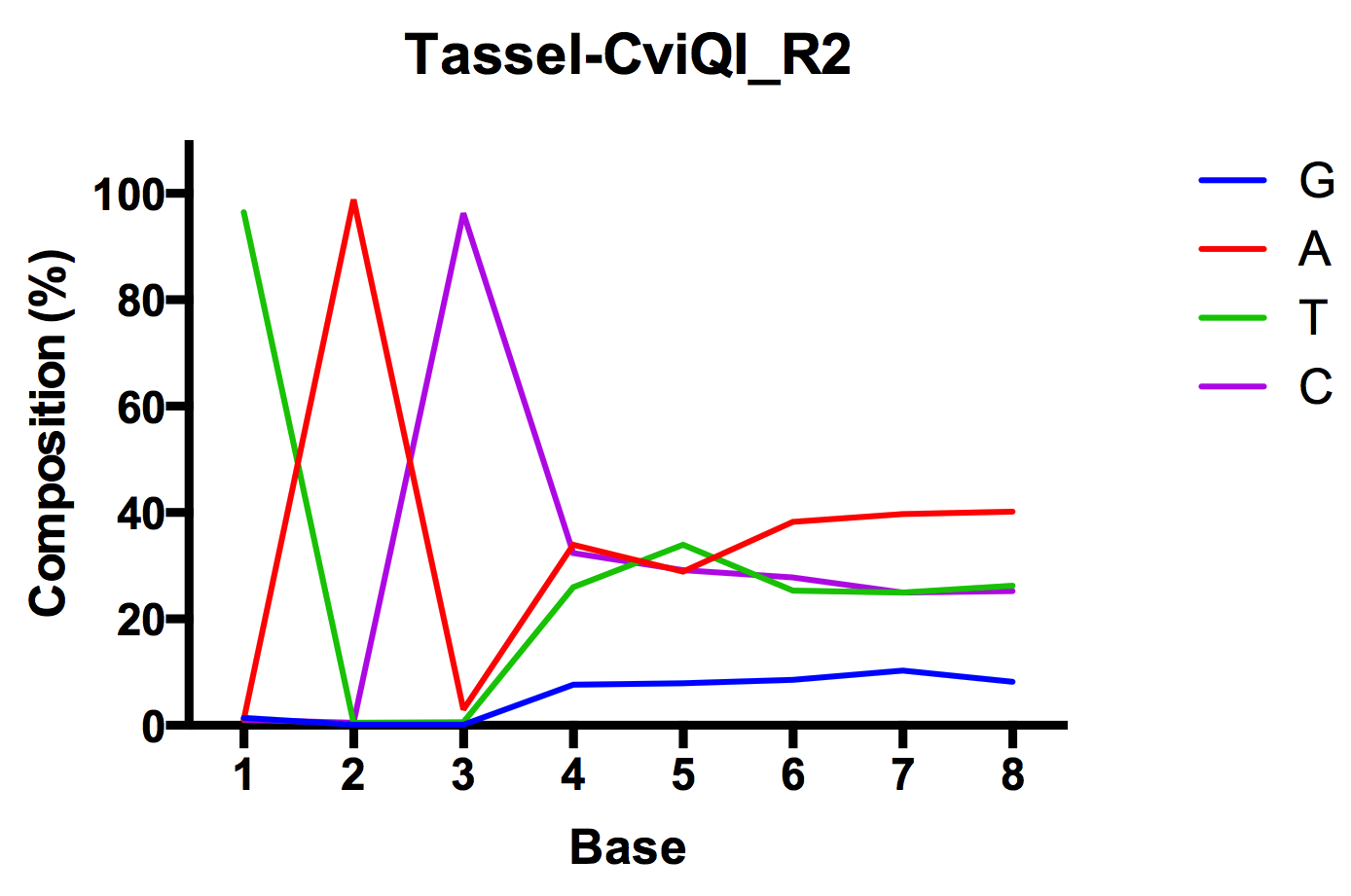

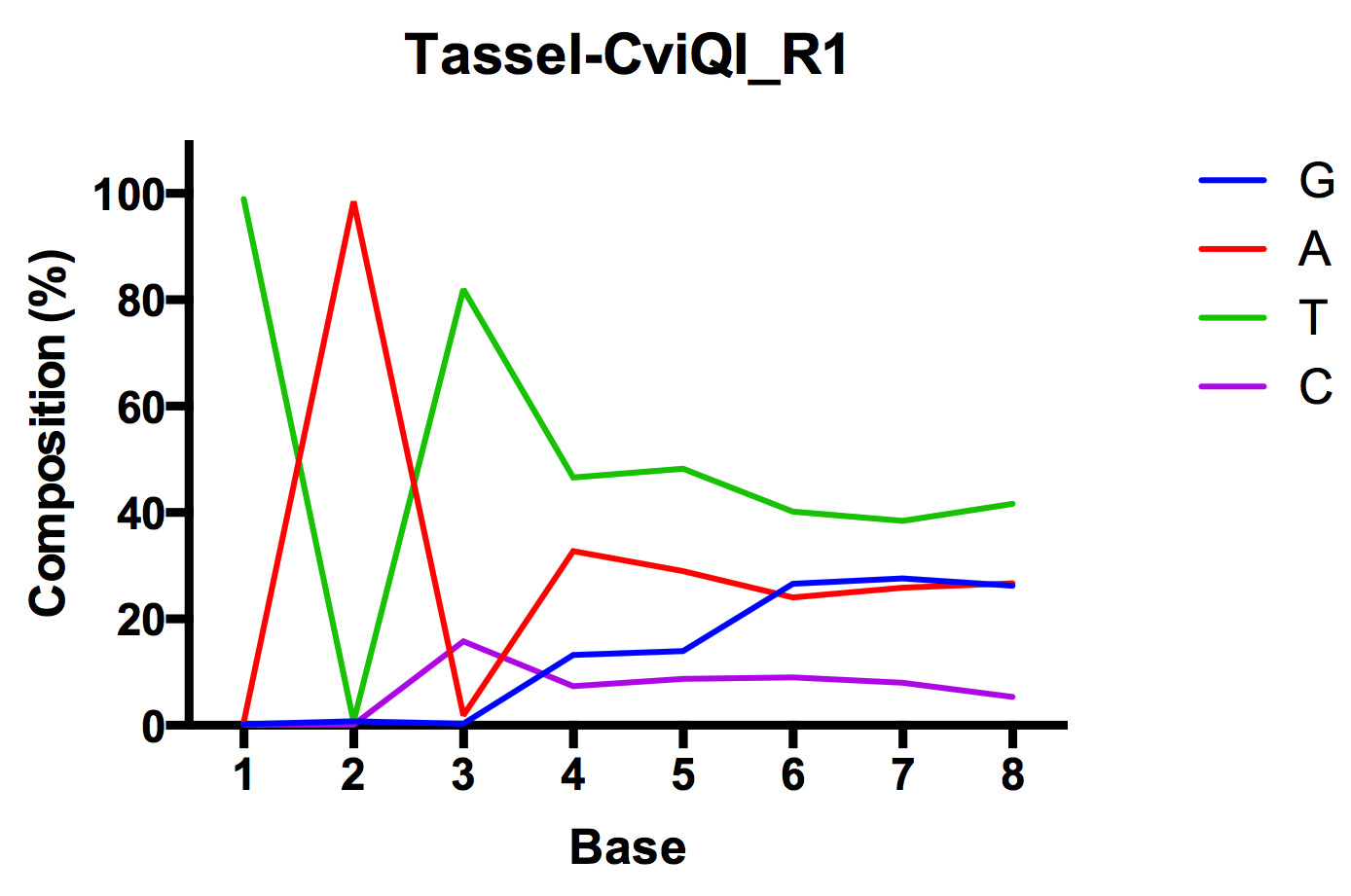


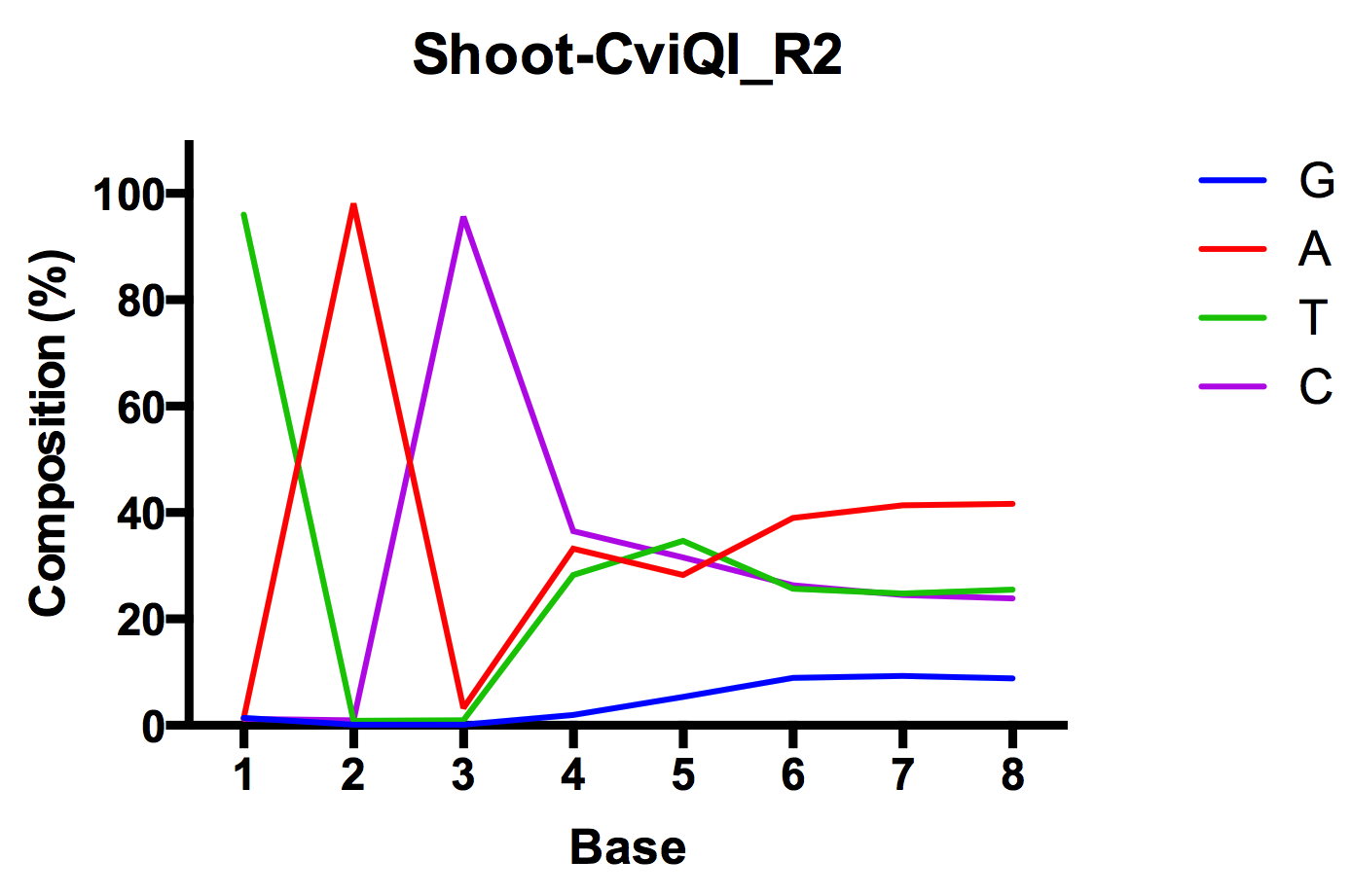

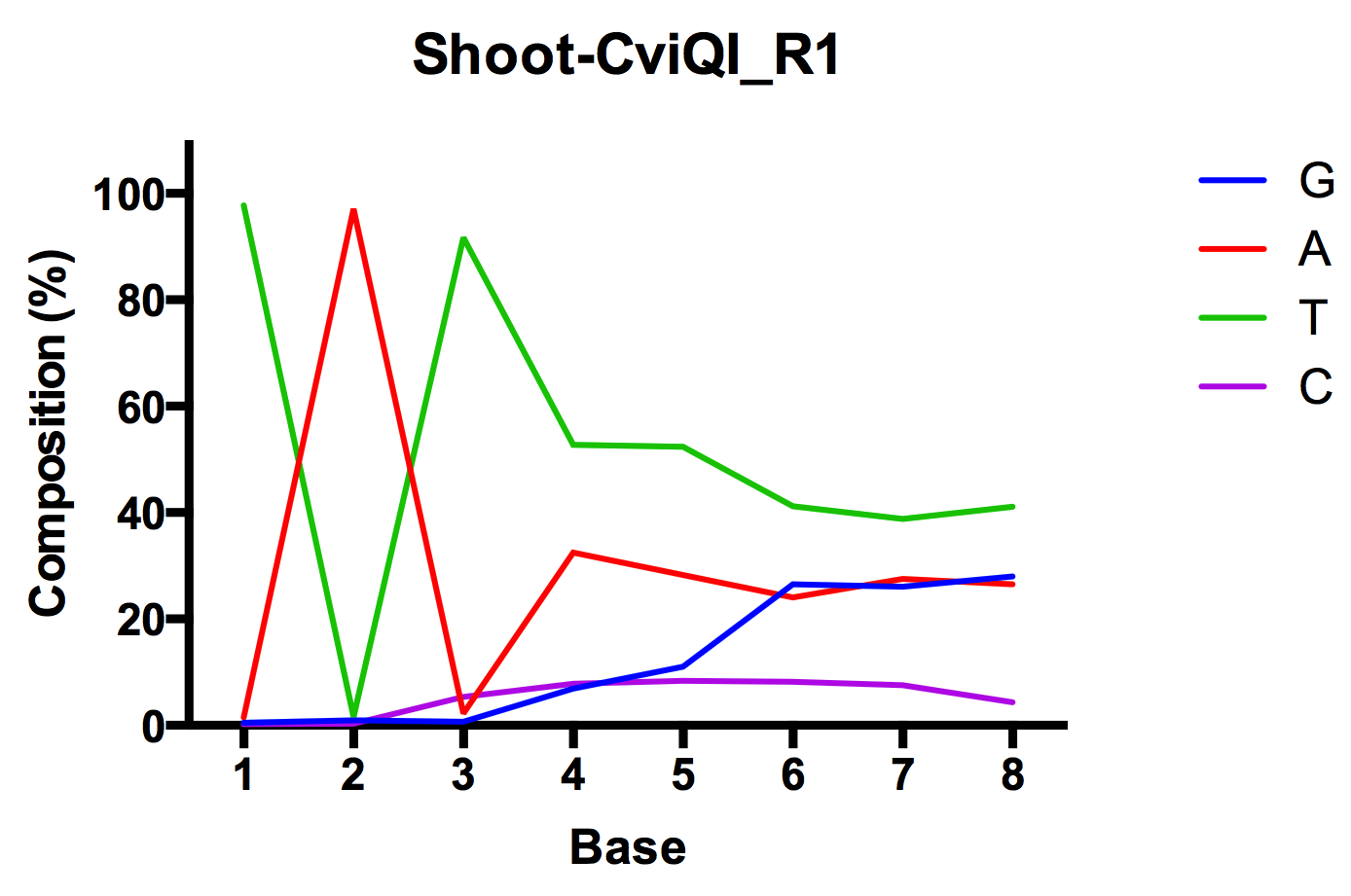


**Figure S2.** Base composition of maize RRBS reads.

**Figure S3**


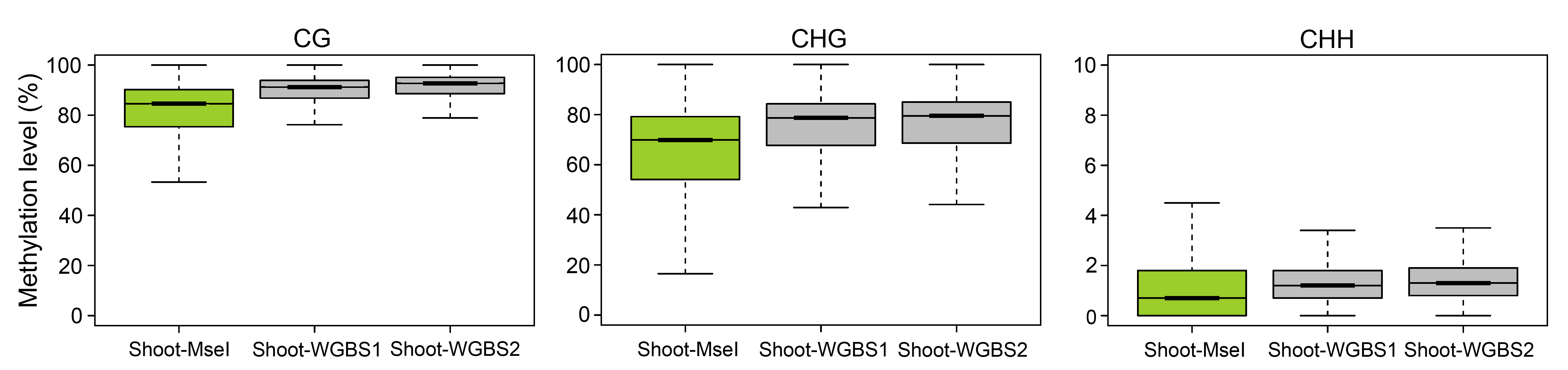


**Figure S3.** Box plot of common sites methylation level in RRBS and WGBS.

**Figure S4**

**Figure S4.** Average methylation level of maize RRBS.

**Figure S5**


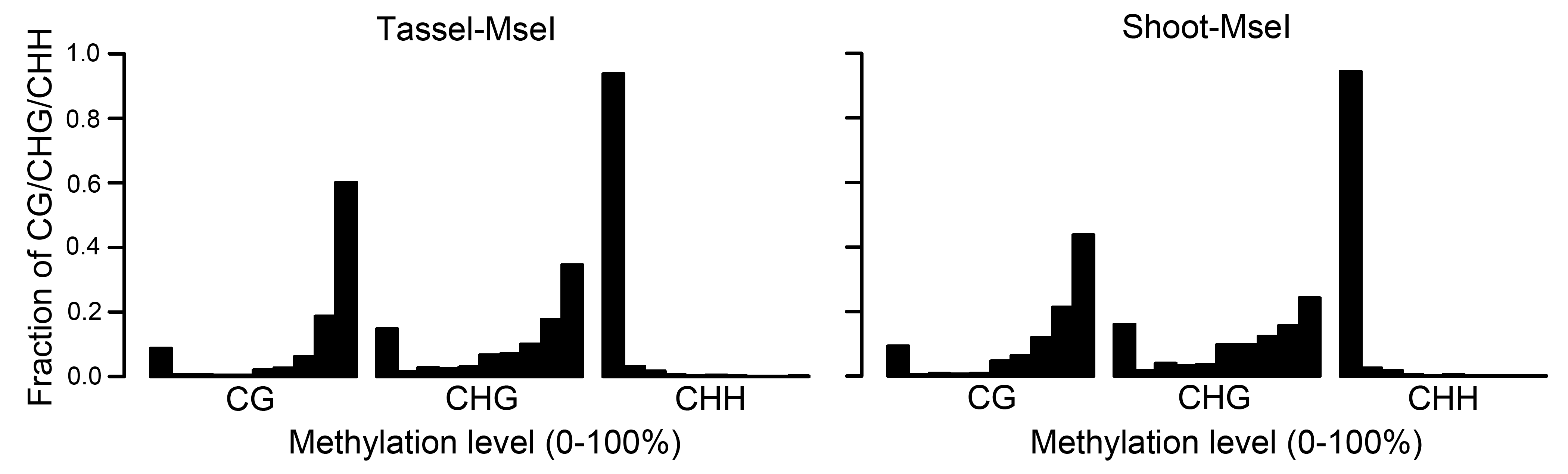


**Figure S5.** Fraction of CG/CHG/CHH methylation level in Tassel-*Mse*I and Shoot-*Mse*I.

Figure S6


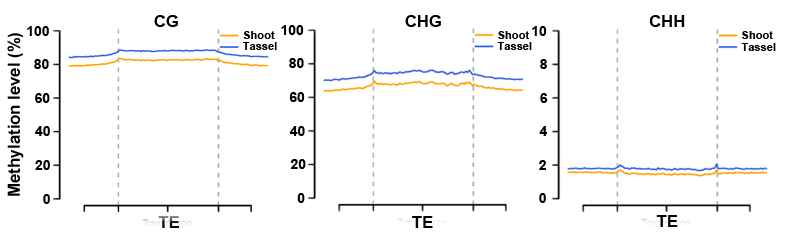


**Figure S6.** Metagene plots of CG, CHG CHH methylation on TE in Shoot- and Tassel-*Mse*I RRBS.

**Figure S7**


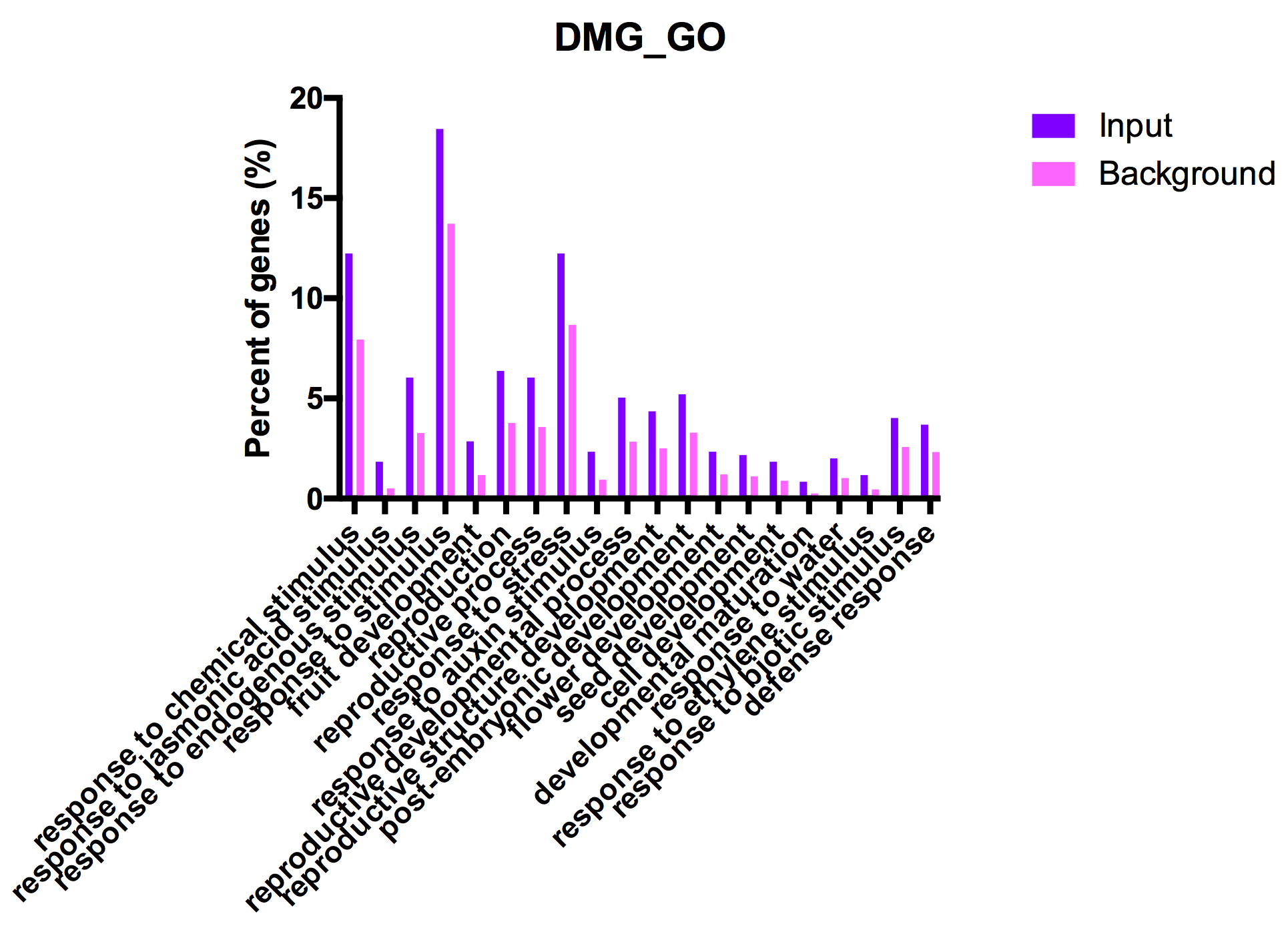


**Figure S7.** GO analysis of DMGs between shoot- and tassel-*Mse*I.

**Figure S8**


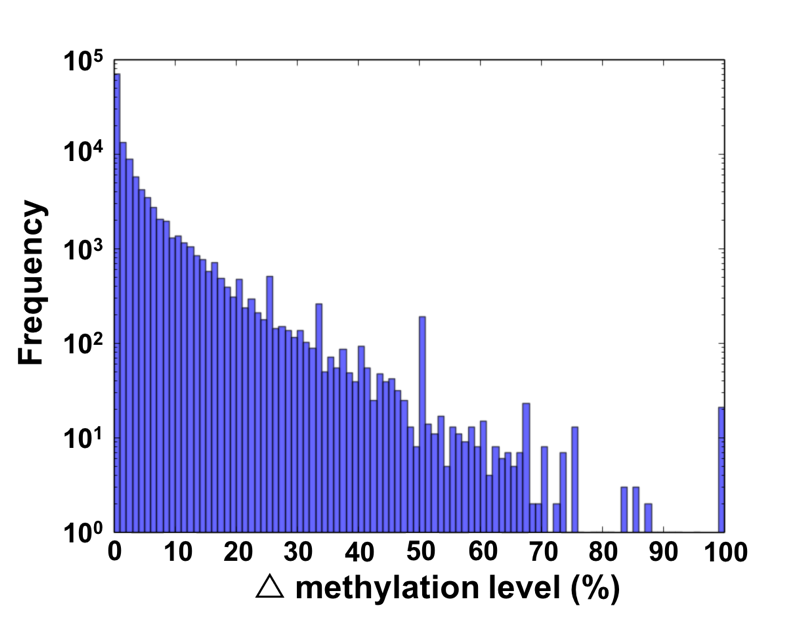


**Figure S8.** Histogram of Δ CHH methylation level of 100bp bins 2kb upstream of TSS between tassel-*Mse*I and shoot-*Mse*I.

**Figure S9**


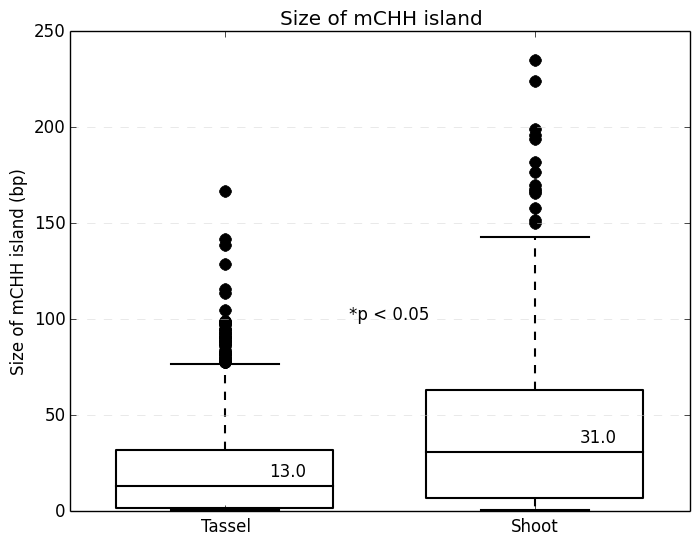


**Figure S9.** Size of mCHH islands in tassel and shoot.

**Figure S10**


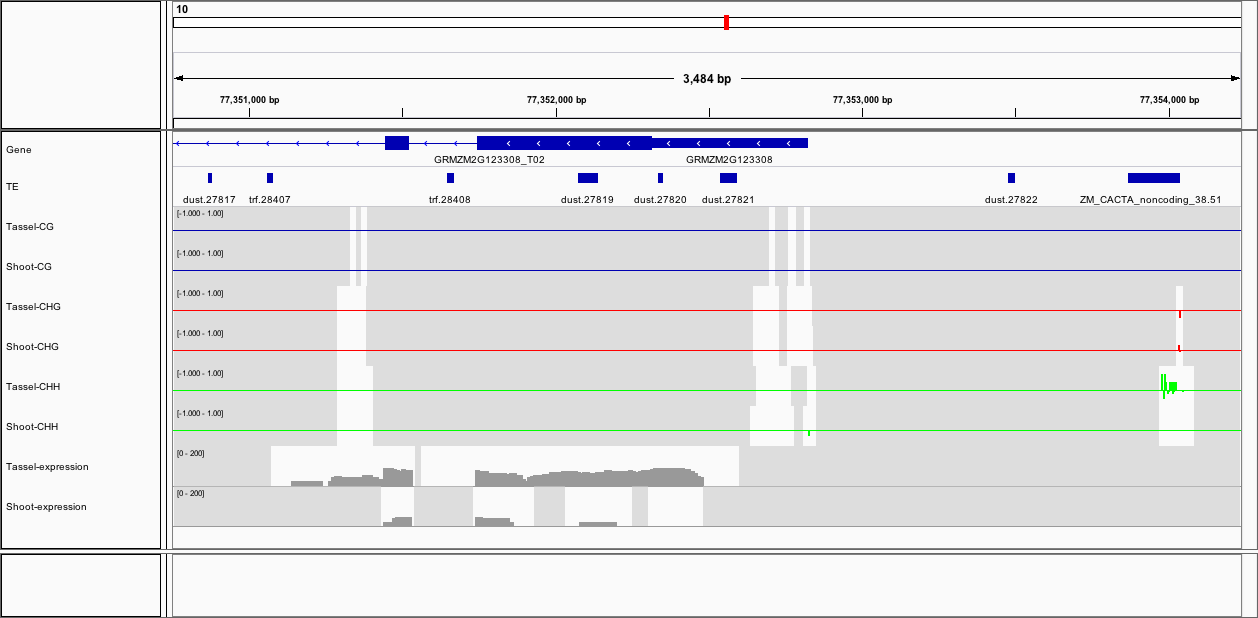


**Figure S10.** An example of a gene GRMZM2G123308 with mCHH island in tassel is up-regulated in tassel.

**Figure S11**


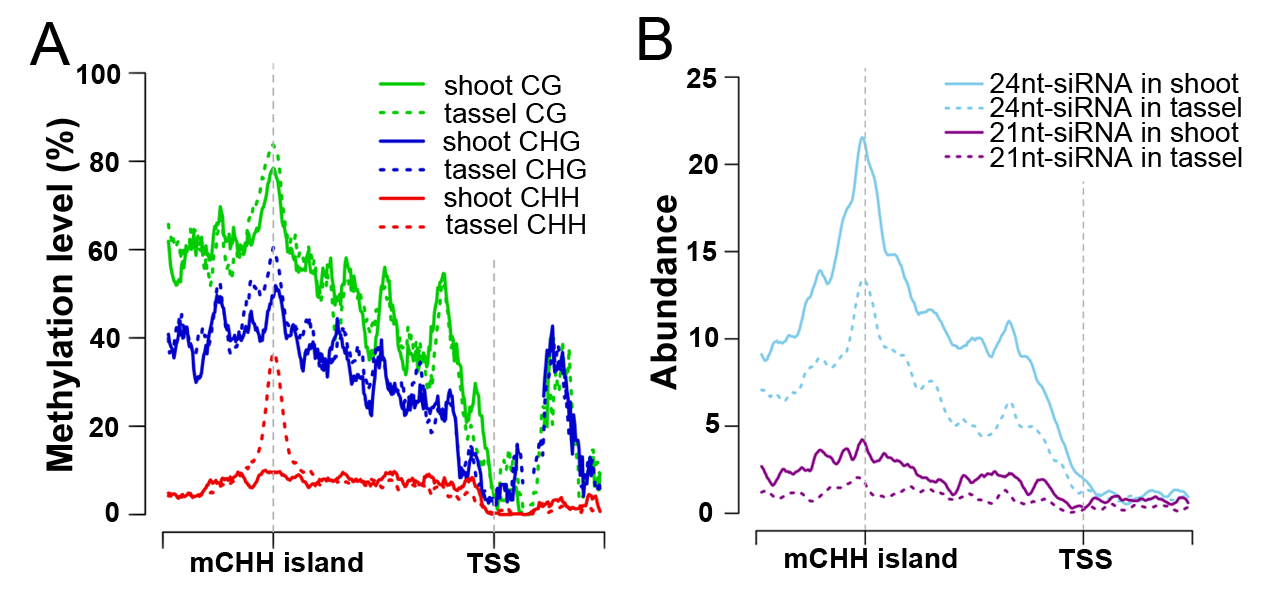


**Figure S11.** Metaplots of 5’ regulatory regions between tassel mCHH islands and TSSs with CHH methylation and siRNA data. (a) Profiles of DNA methylation levels in shoot and tassel around tassel mCHH islands that are hypermethylated in tassel. (b) Abundance of 21nt- and s4nt-siRNA around mCHH islands that are hypermethylated in tassel.
