# Supplementary material for: Optimized reduced representation bisulfite sequencing reveals tissue-specific mCHH islands in maize
Source: Epigenetics Chromatin. 2017 Aug 30;10:42. doi: 10.1186/s13072-017-0148-y (PMC5577757; doi:10.1186/s13072-017-0148-y)
Supplement: Supplementary file 4 — Additional file 4. Results of CviQI-RRBS. [file 13072_2017_148_MOESM4_ESM.docx]

**Addition file 4**

***Cvi*QI-RRBS**


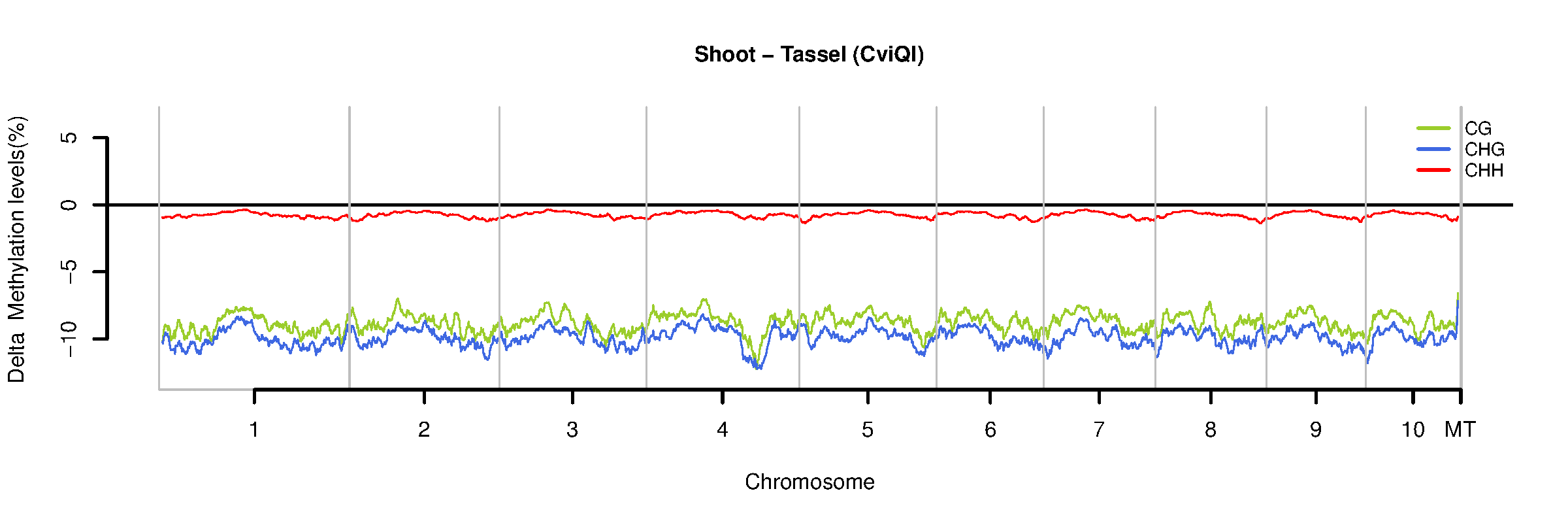


**Figure legend**

Δ methylation level of tassel and shoot *Cvi*QI-RRBS


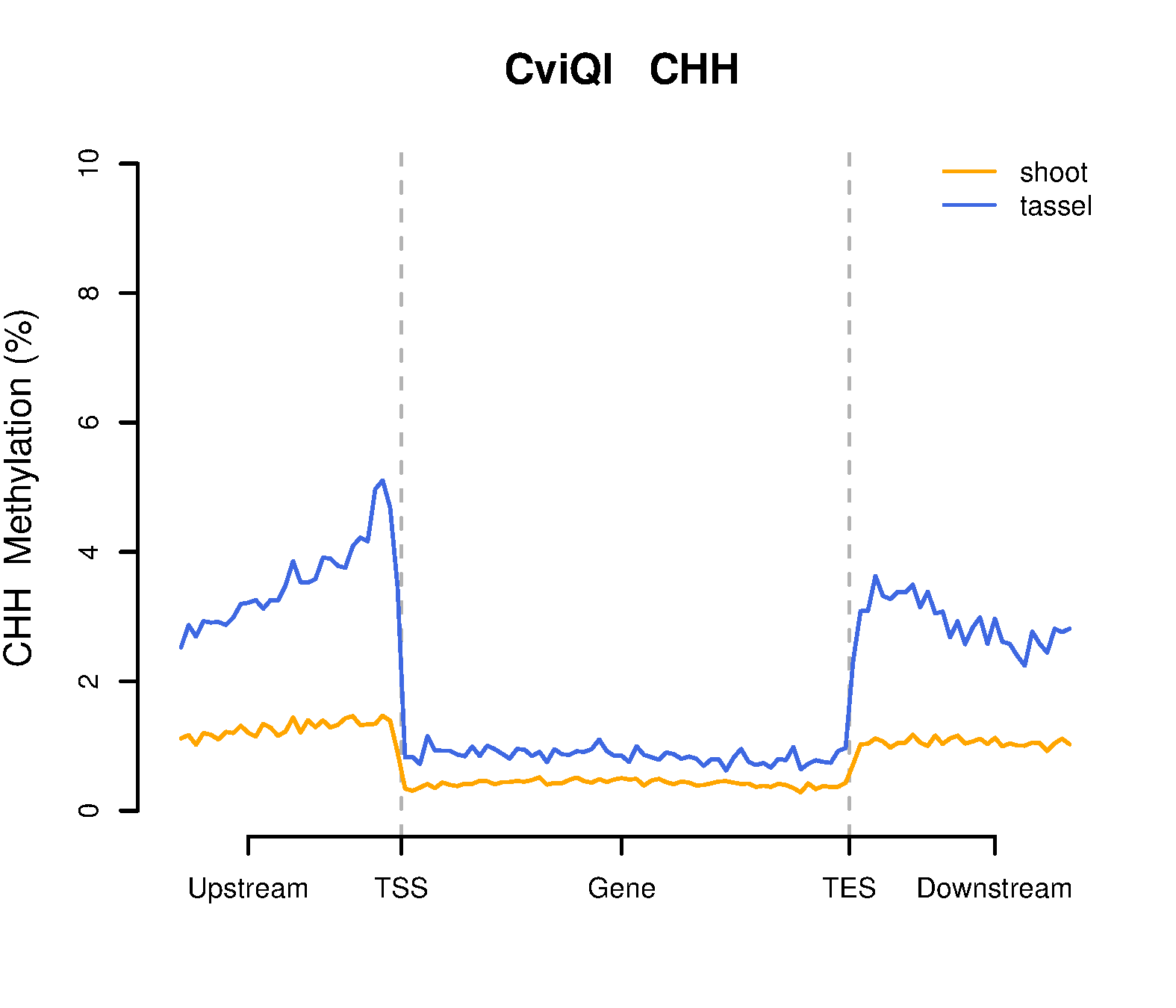

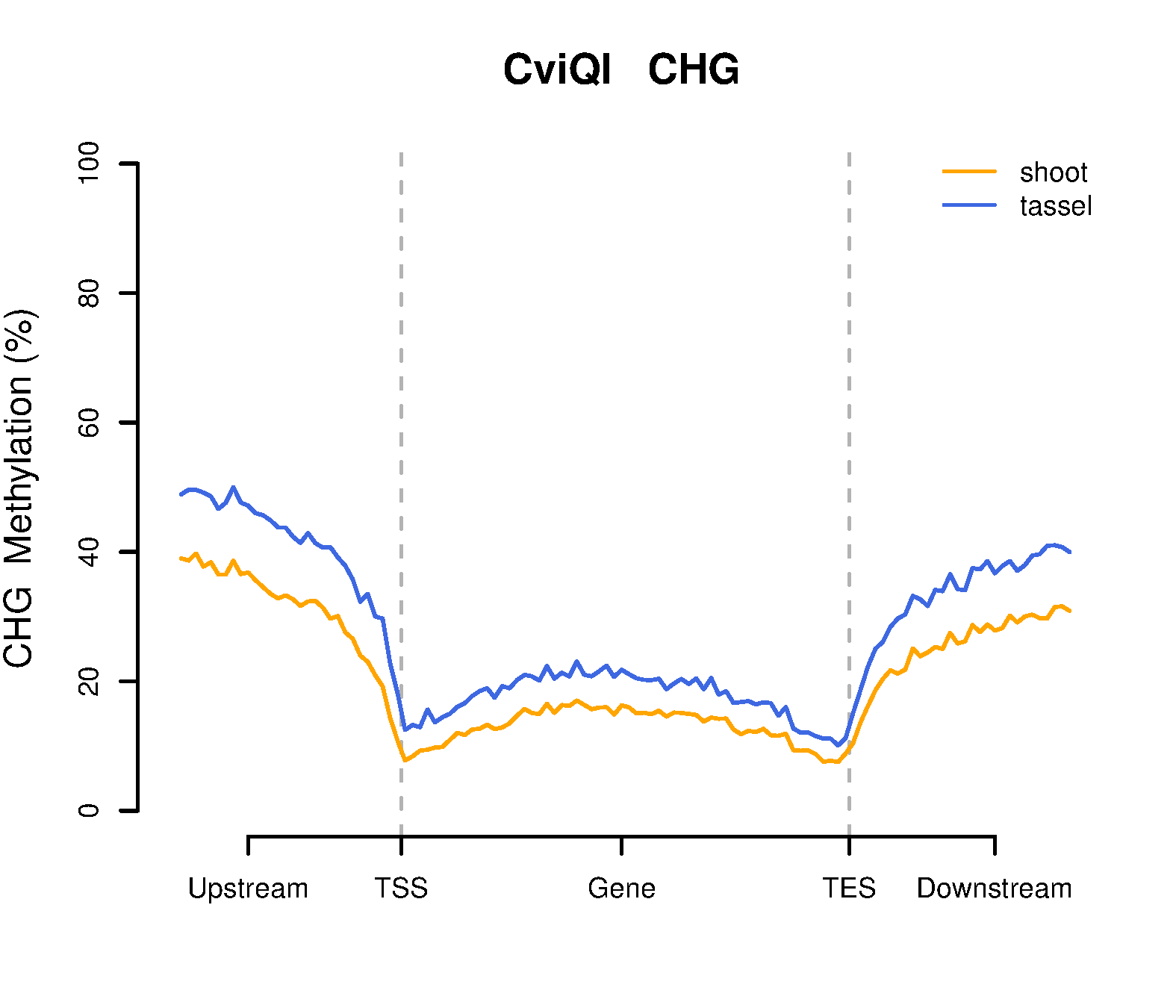

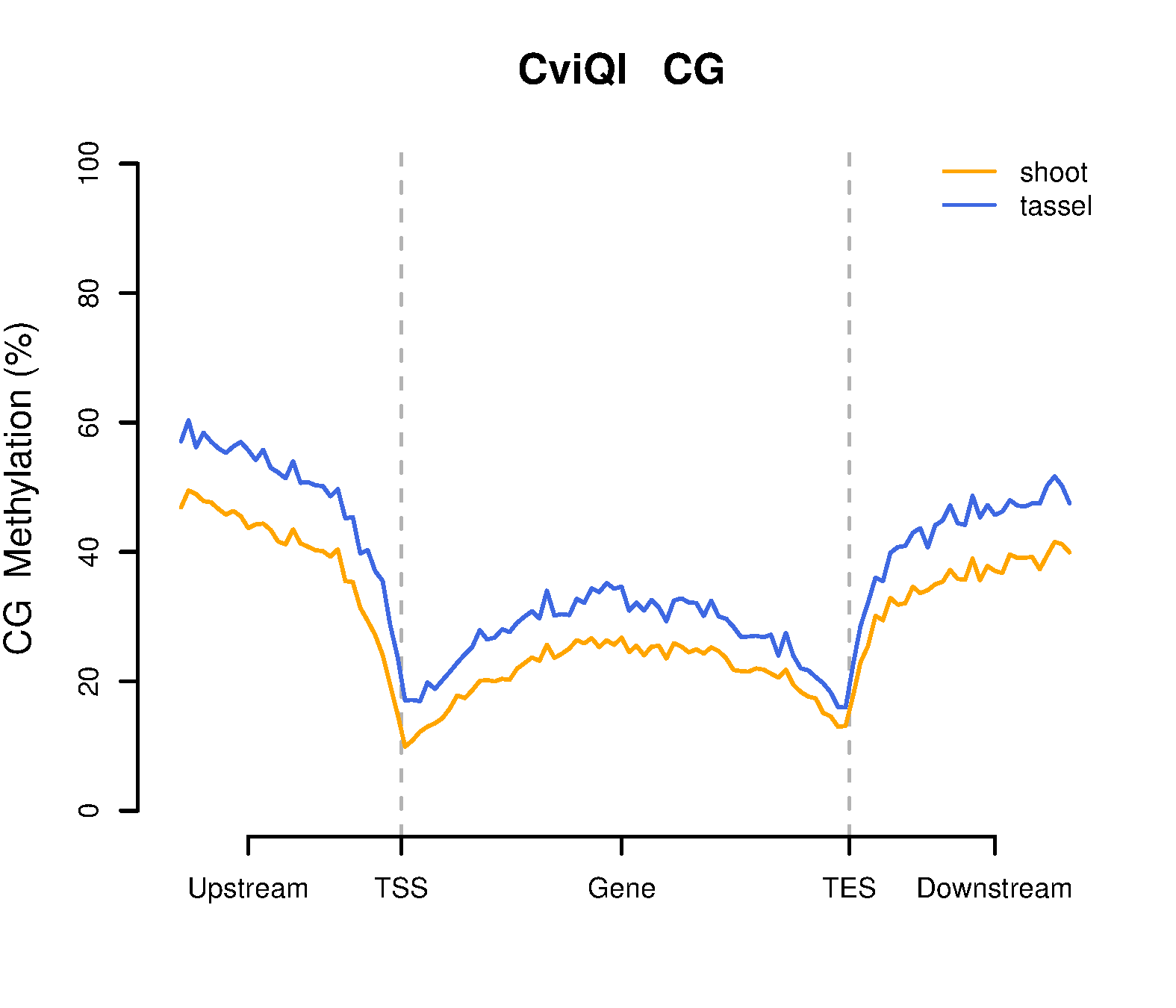


**Figure legend**

Metagene plots of CG, CHG CHH methylation on gene in Shoot- and Tassel-*Cvi*QI RRBS.


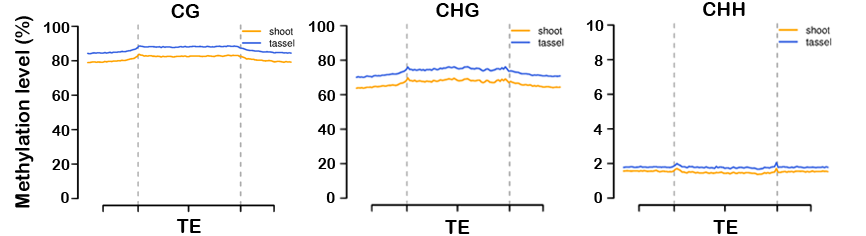


**Figure legend**

Metagene plots of CG, CHG CHH methylation on TE in Shoot- and Tassel-*Cvi*QI RRBS.

We found 7,505 DMRs between shoot- and tassel-*Cvi*QI, and ~82% DMRs are in non-CG contexts.


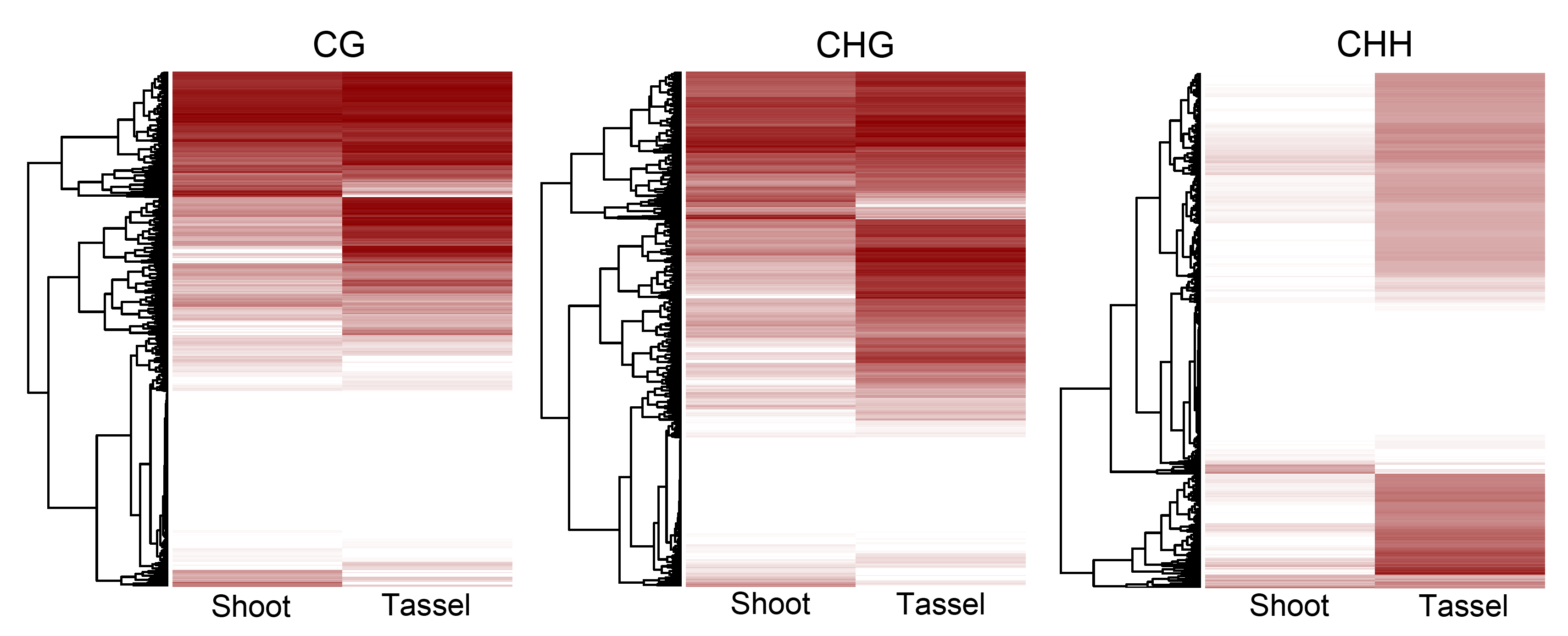


**Figure legend**

Heatmaps of *Cvi*QI-RRBS.


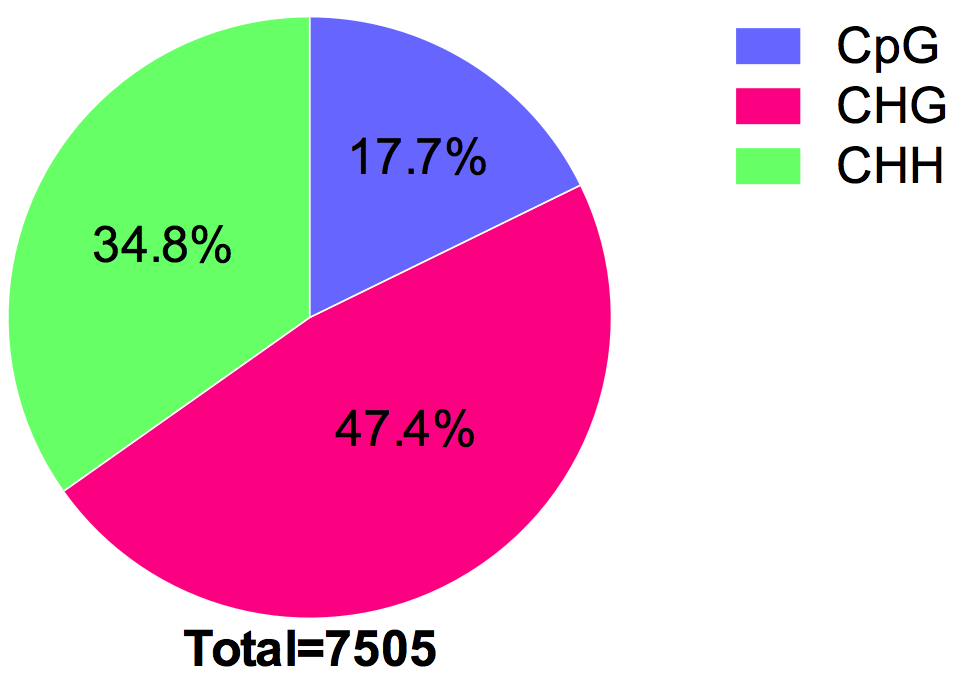


**Figure legend**

Fraction of DMR in *Cvi*QI-RRBS.

We found that CHH DMRs show a strong enrichment in promoters and UTR regions even though the *Cvi*QI-RRBS concentrates gene body region. This result indicates again that the methylation alterations primarily occur in promoter regions.


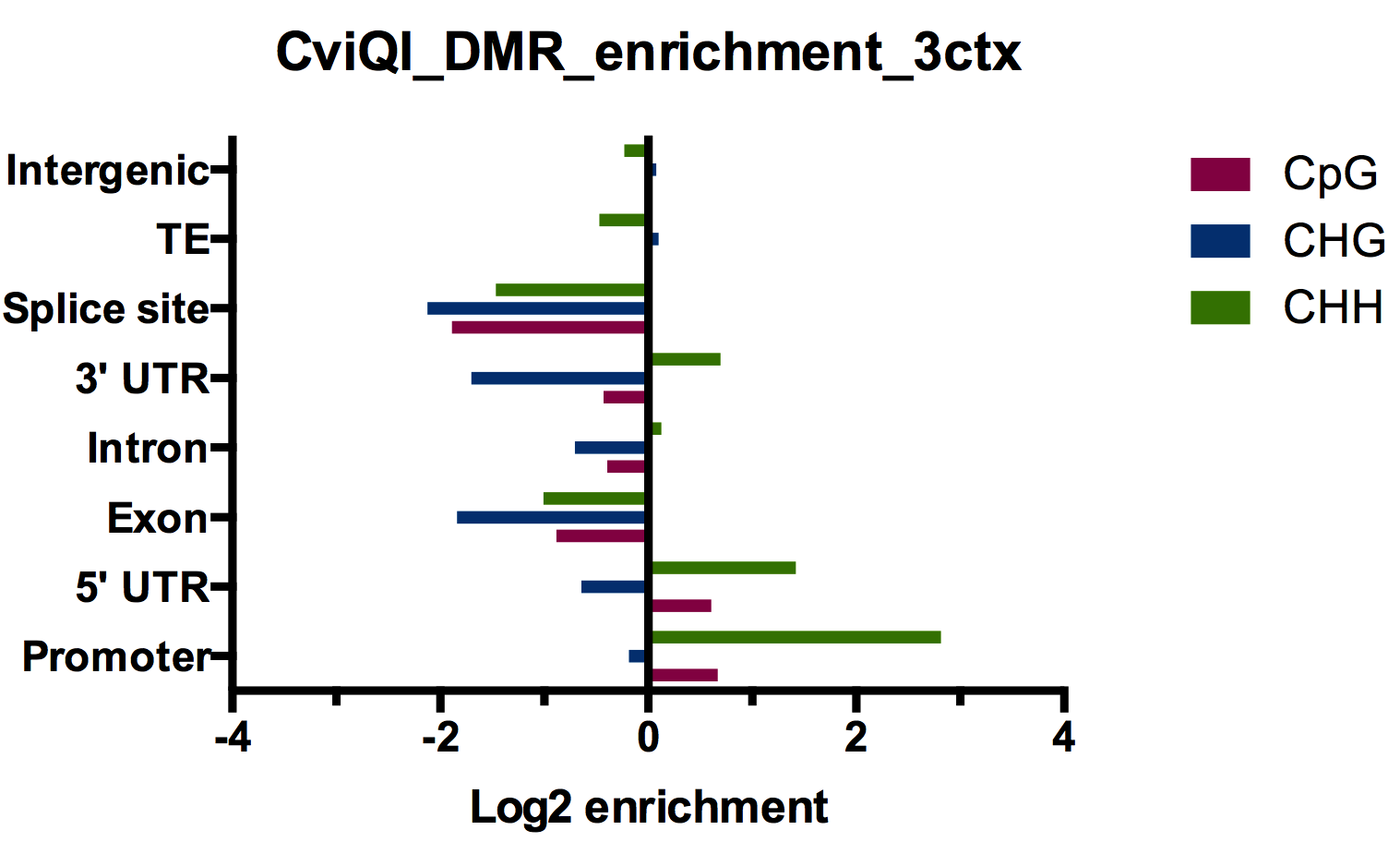


**Figure legend**

DMR enrichment analysis in *Cvi*QI-RRBS.

The DMRs in *Cvi*QI-RRBS locate in the promoter or genebody of total 1,405 DMGs (Additional file 2). Of which, 933 DMGs show differential methylation in promoter and 489 in genebody. To understand possible functions of these DMGs identified in comparison of shoot and tassel, we performed functional annotation for gene ontology (GO) analysis using AgriGO for the DMGs of *Mse*I-RRBS and *Cvi*QI-RRBS. We found most DMGs are related to development and reproduction, suggesting that components in these biological processes are differentially regulated by DNA methylation in shoot and tassel.

GO enrichment of DMGs from *Mse*I-RRBS and *Cvi*QI

| GO accession | GO term | *p* value |
| --- | --- | --- |
| 0048856 | Anatomical structure development | 9.5e-06 |
| 0048608 | Reproductive structure development | 2.3e-05 |
| 0003006 | Reproductive developmental process | 1.6e-05 |
| 0010154 | Fruit development | 2.1e-05 |
| 0032501 | Multicellular organismal process | 2.4e-05 |
| 0009791 | Post-embryonic development | 4.7e-06 |
| 0007275 | Multicellular organismal development | 2.9e-05 |
| 0022414 | Reproductive process | 4e-05 |
| 0032502 | Developmental process | 4.8e-05 |
| 0000003 | Reproduction | 5.4e-05 |
