# Supplementary material for: Optimized reduced representation bisulfite sequencing reveals tissue-specific mCHH islands in maize
Source: Epigenetics Chromatin. 2017 Aug 30;10:42. doi: 10.1186/s13072-017-0148-y (PMC5577757; doi:10.1186/s13072-017-0148-y)
Supplement: Supplementary file 5 — Additional file 5. Results of TE methylation profiling. [file 13072_2017_148_MOESM5_ESM.docx]

**Additional file 5**

**TE methylation in shoot and tassel**

In maize, more than 80% of the genome consists of transposable elements, including 75% of class I retrotranspons (RTE) and ~8.6% class II DNA transposons (DTE) [1]. We plotted the methylation level of major TE families including RTEs grouped into LINEs, LTRs and SINEs, and also DTEs. The result showed that tassel is more methylated than shoot in these four TE families.


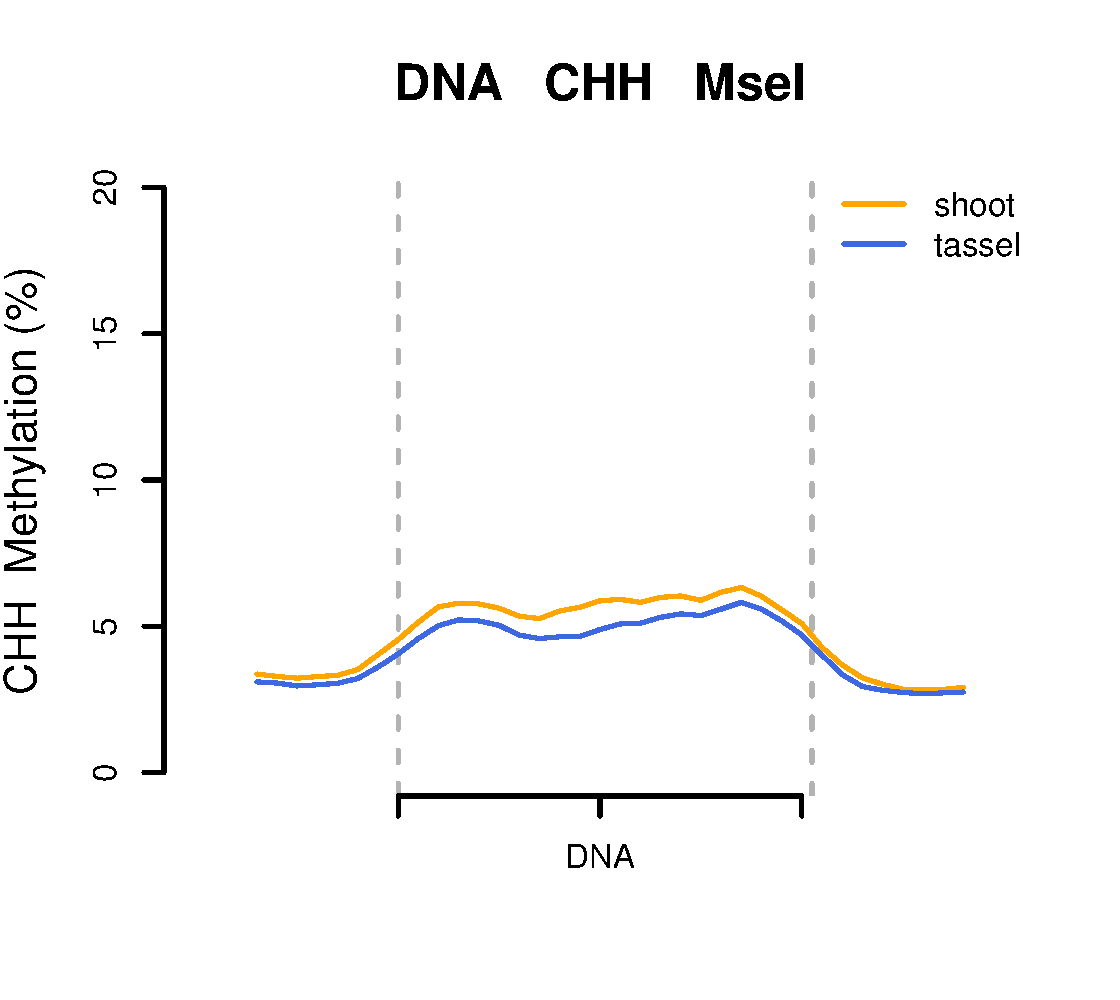

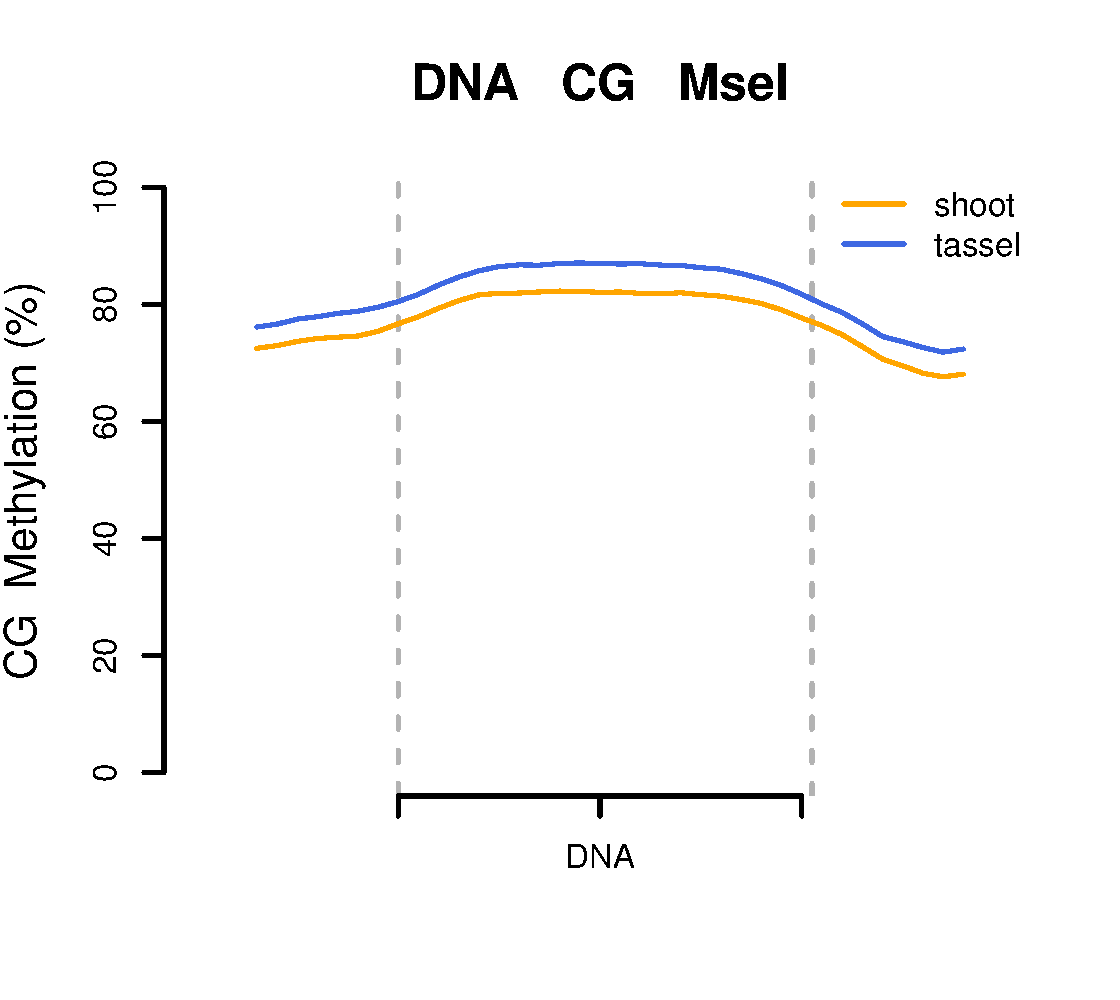

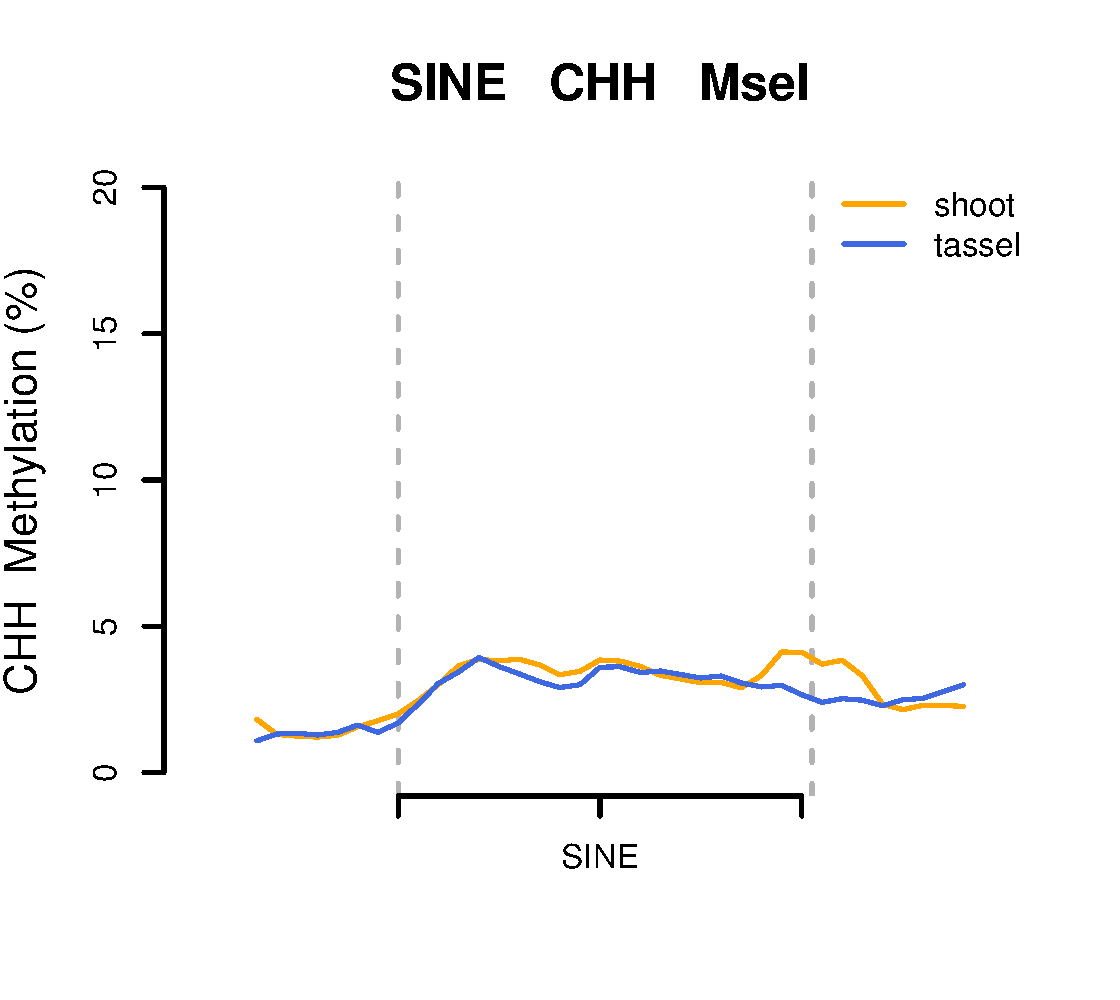

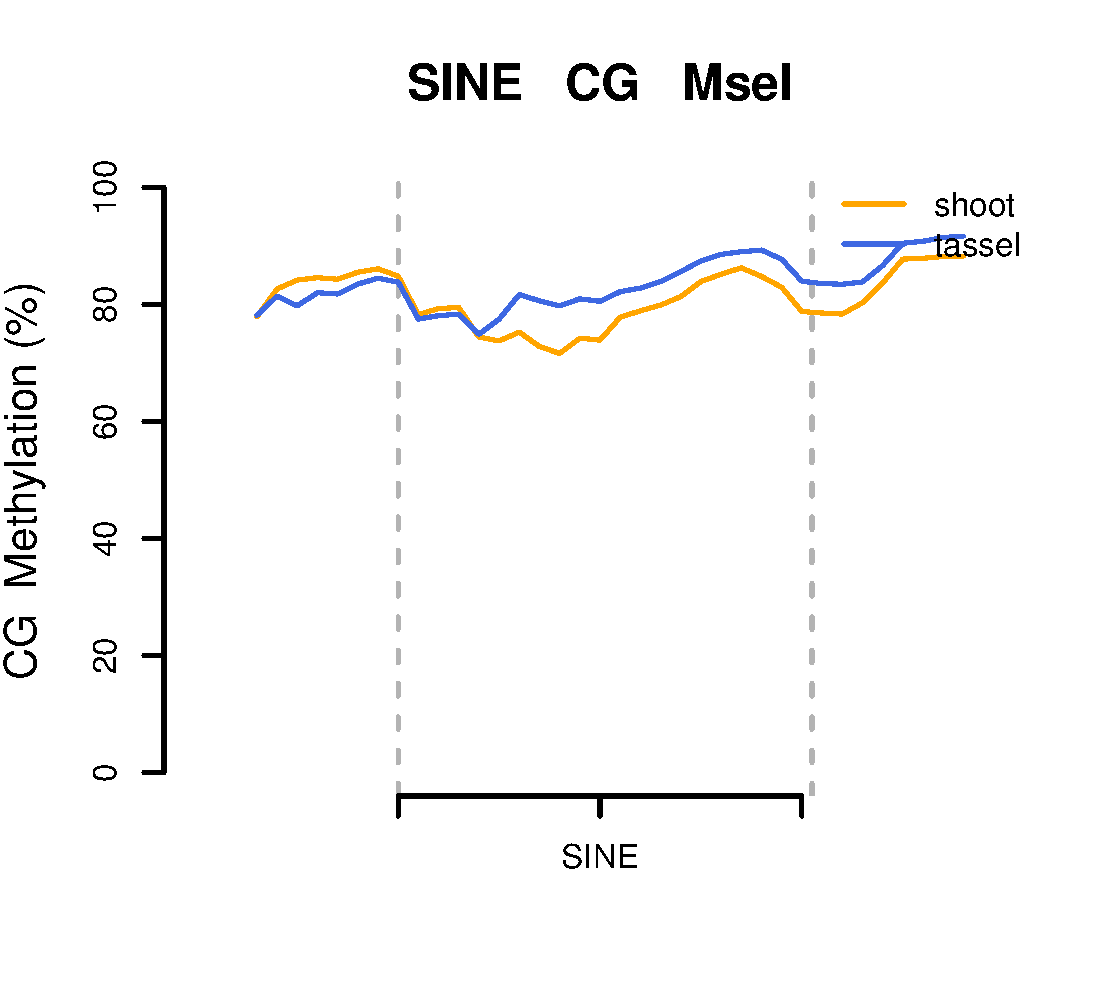

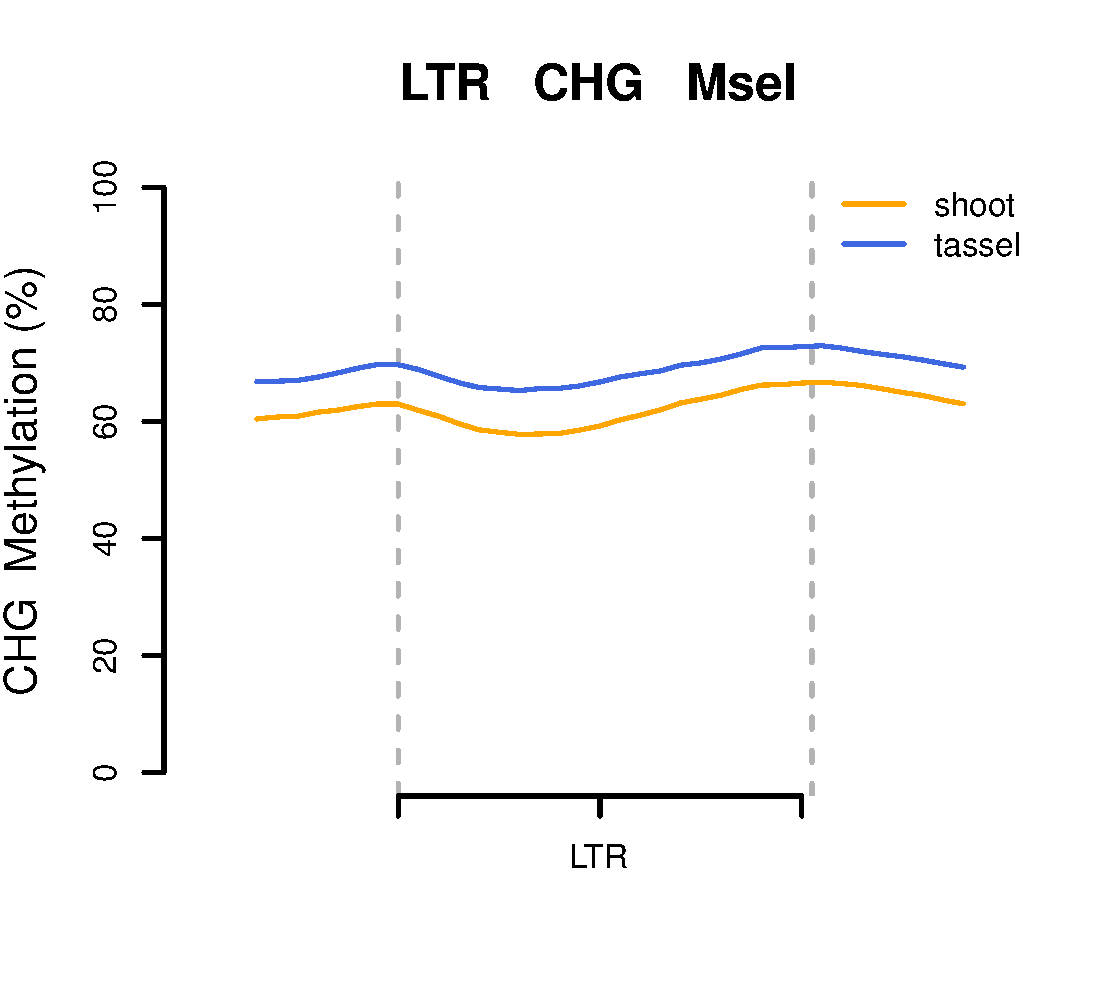

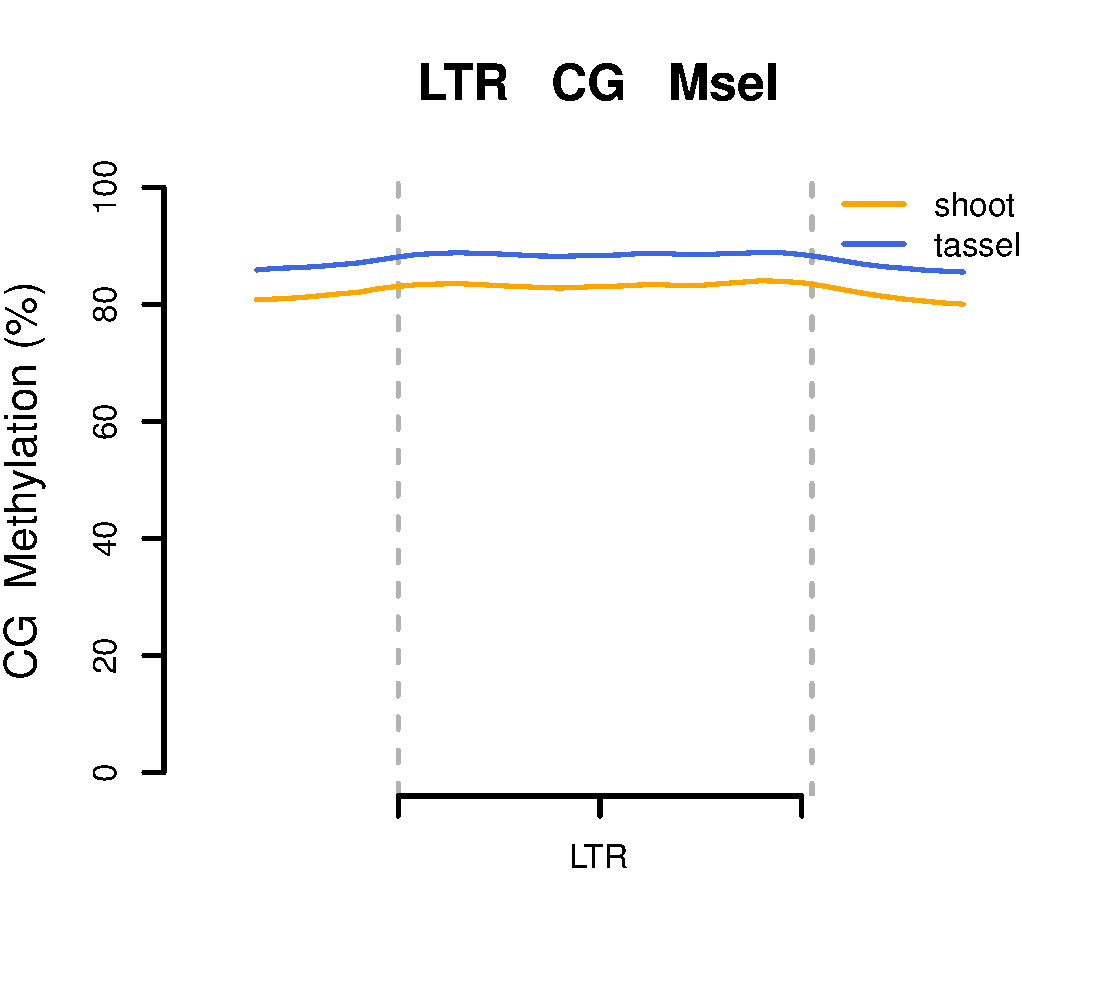

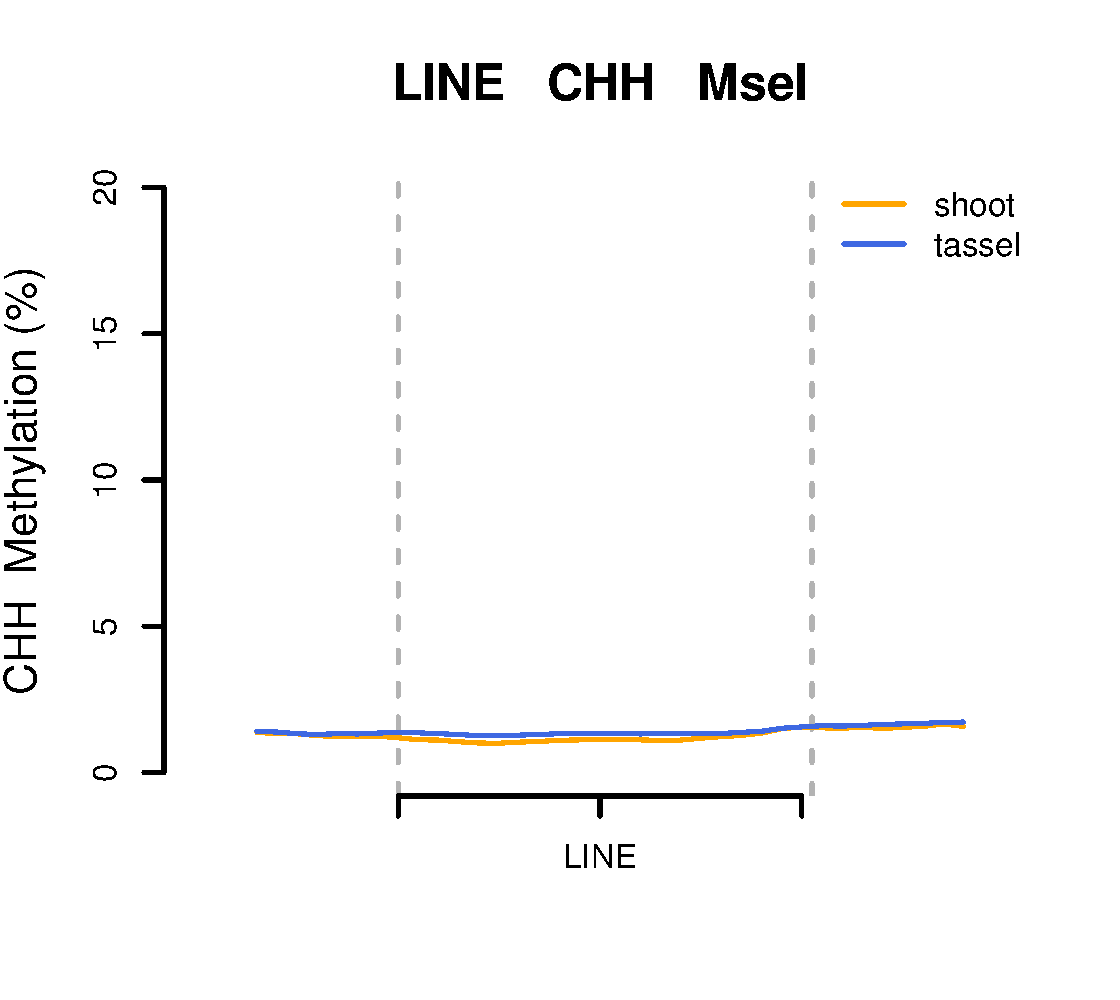

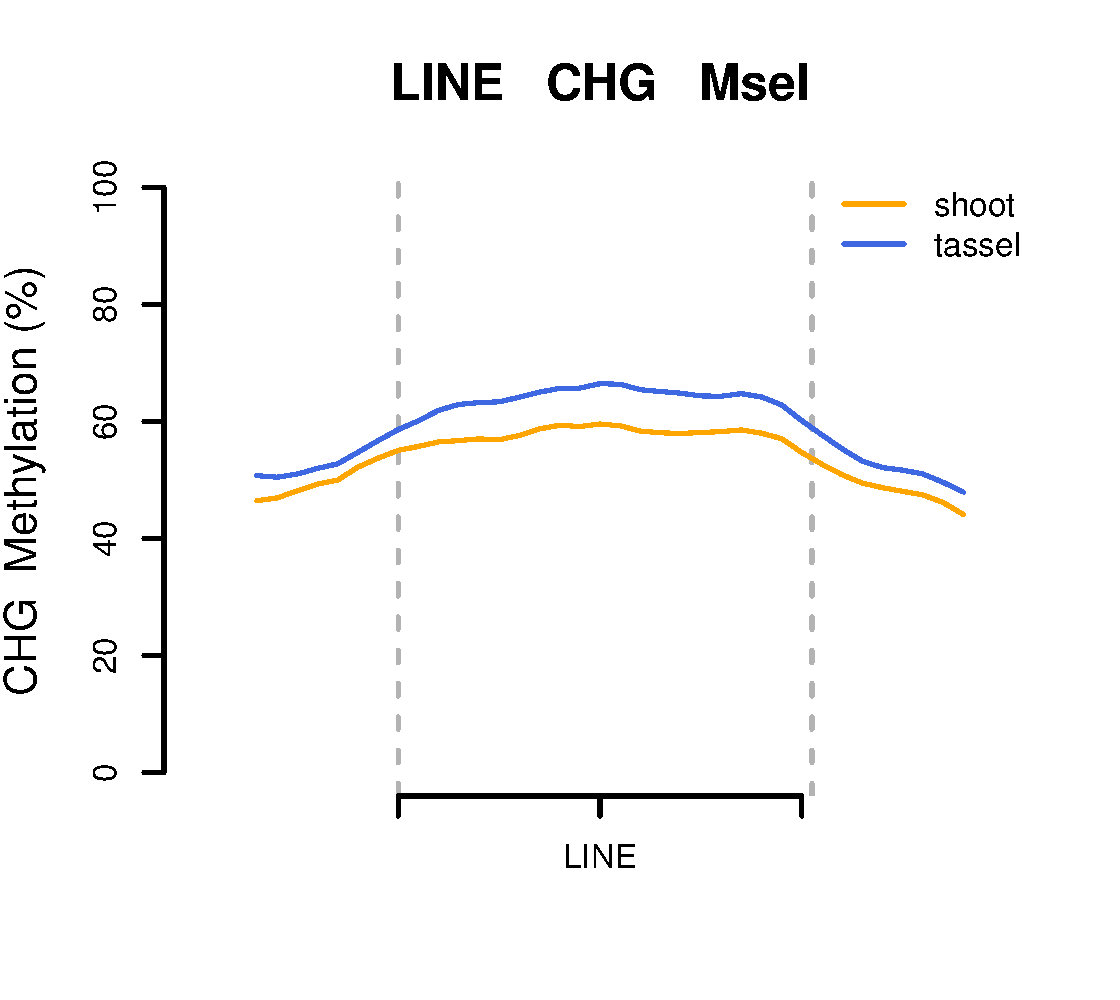

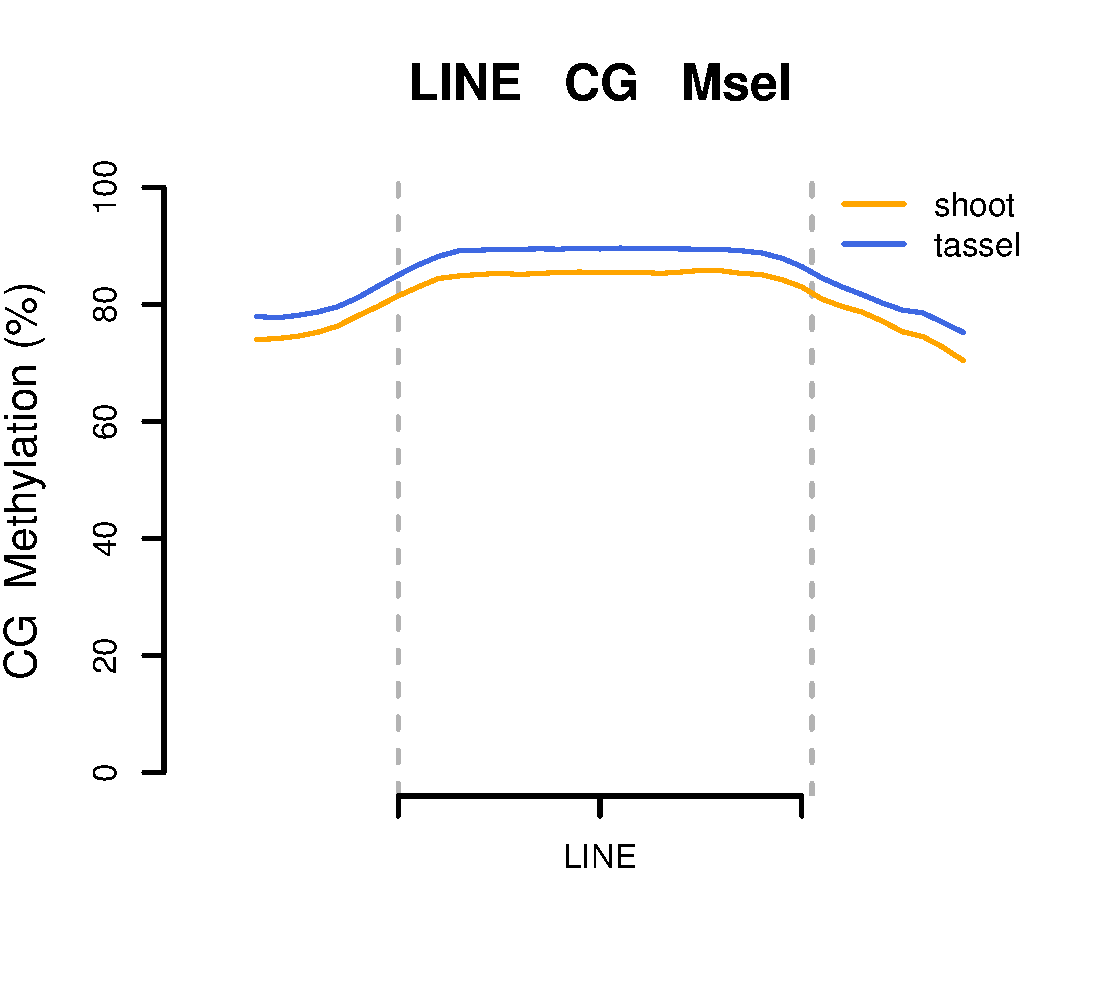


A

B


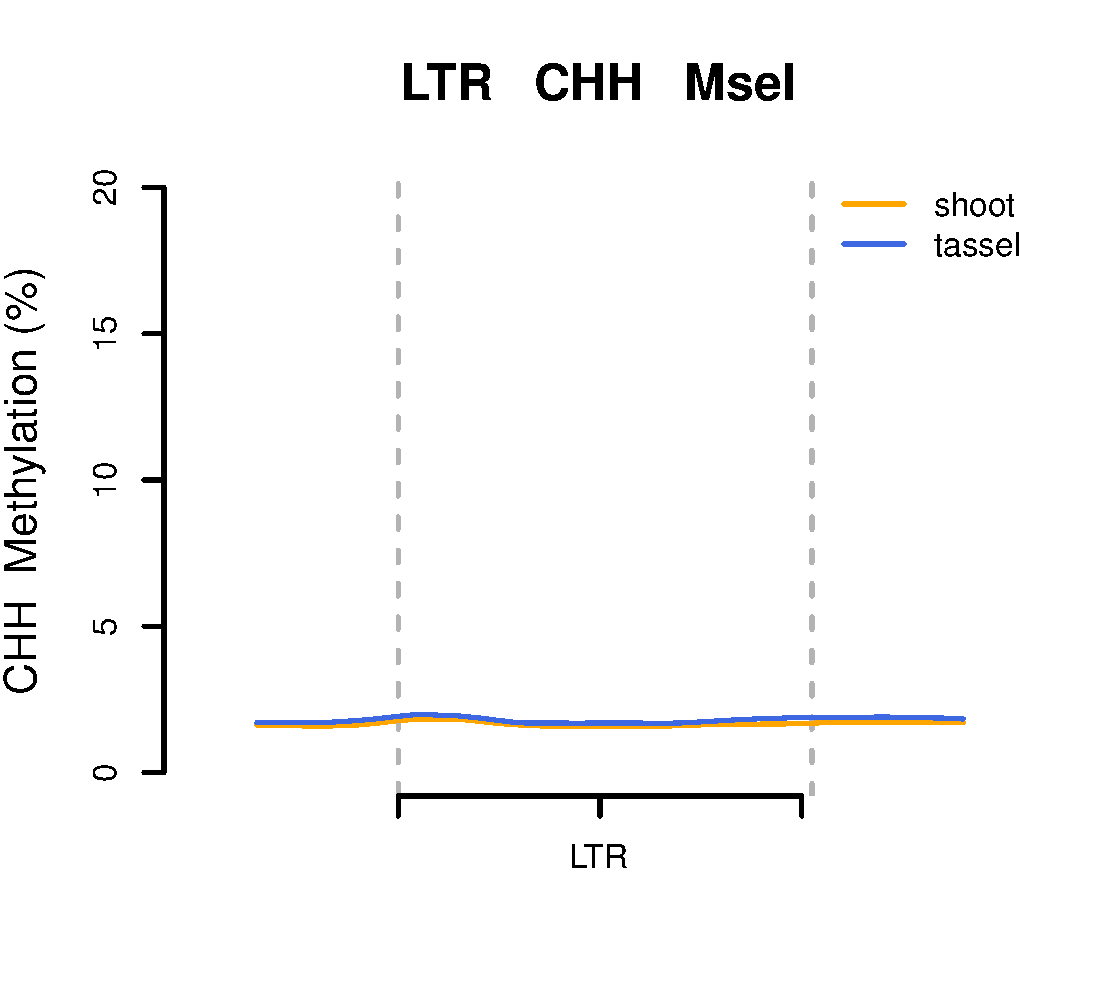


C


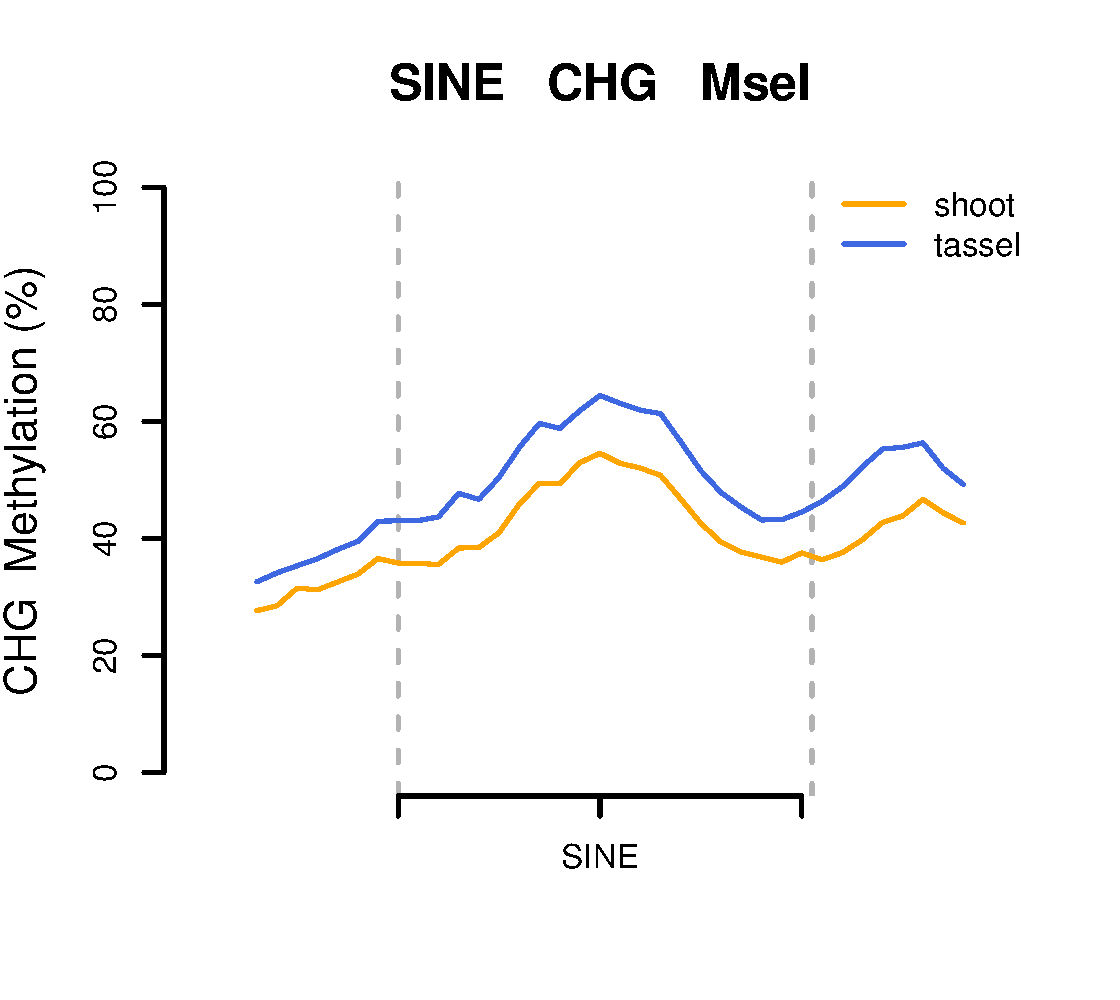


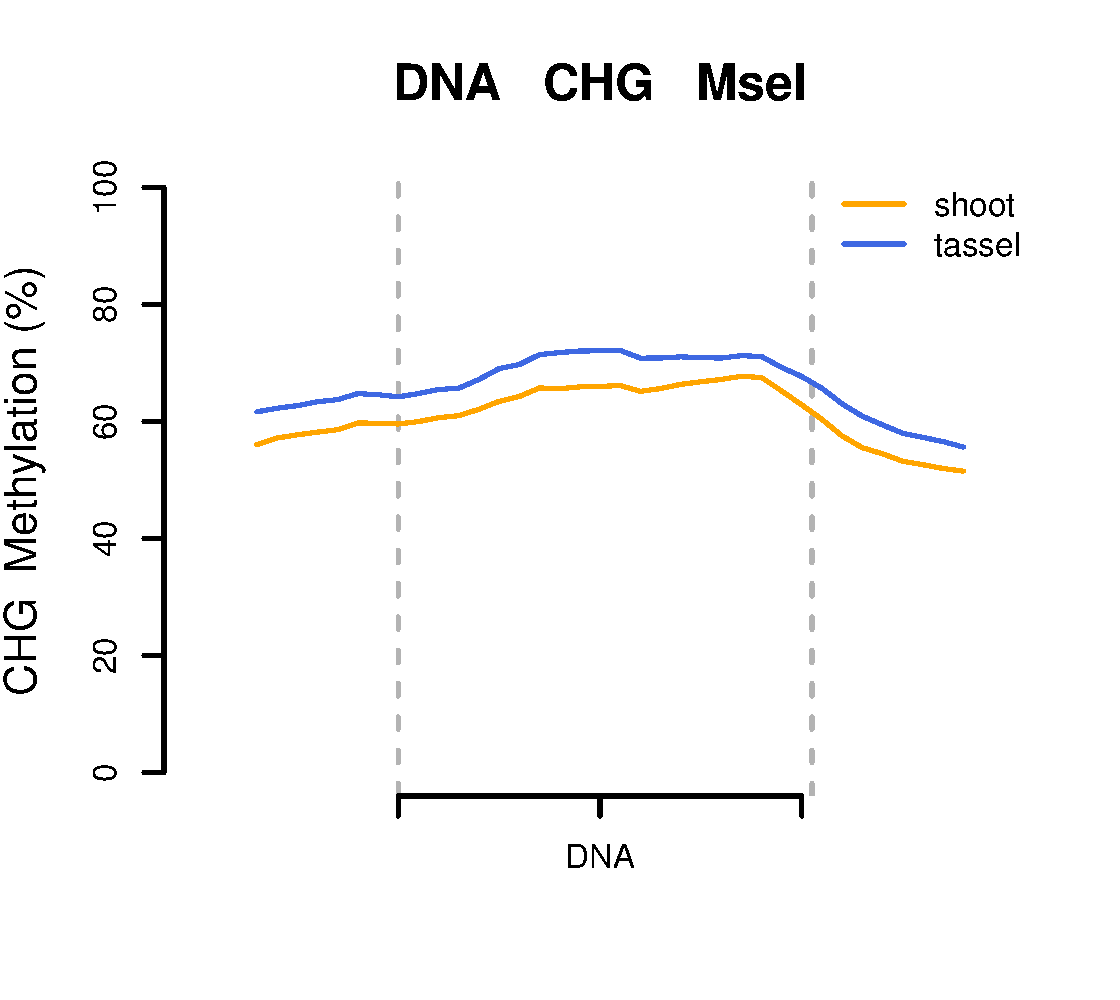


D

**Figure Legend**

**Metagene plot of methylation level in transposons.** (**a**) DNA transposon. (**b**) LTR. (**c**) LINE. (**d**) SINE.

In LINE family, CG and CHG methylation level increase in the body of transposons, whereas CHH methylation level shows no significant differences between the body and flanking sequence. The CHG methylation increment trend is more obvious in tassel than in shoot, indicating that in reproductive stage, LINEs are even methylated in CHG context than in vegetative stage.

SINEs, however, different from LINEs and LTRs, show rugged patterns of methylation in all three contexts. In *Mse*I-RRBS, CG methylation has a decreasing pattern in the beginning and the end of SINE body, and shoot shows higher methylation upstream of SINEs. CHG has an increasing pattern in the beginning of SINE, reaches the peak in the middle of SINE body, and decreases to the lowest level in the end of SINE. CHH methylation level elevates in the body of SINE and this pattern is even obvious in DTEs.

Taken together, we found that between shoot and tassel, in RTEs, LINEs are mainly regulated by and CHG methylation; LTRs have large amounts, and we can only identify *panen*, *sawuju*, *udav*, *utar*, *vedi* and *yraj* as possible regulators when further profiling in each family; SINEs are possibly regulated by all three contexts methylation; TEs are mainly regulated by CHH methylation as previously published results.


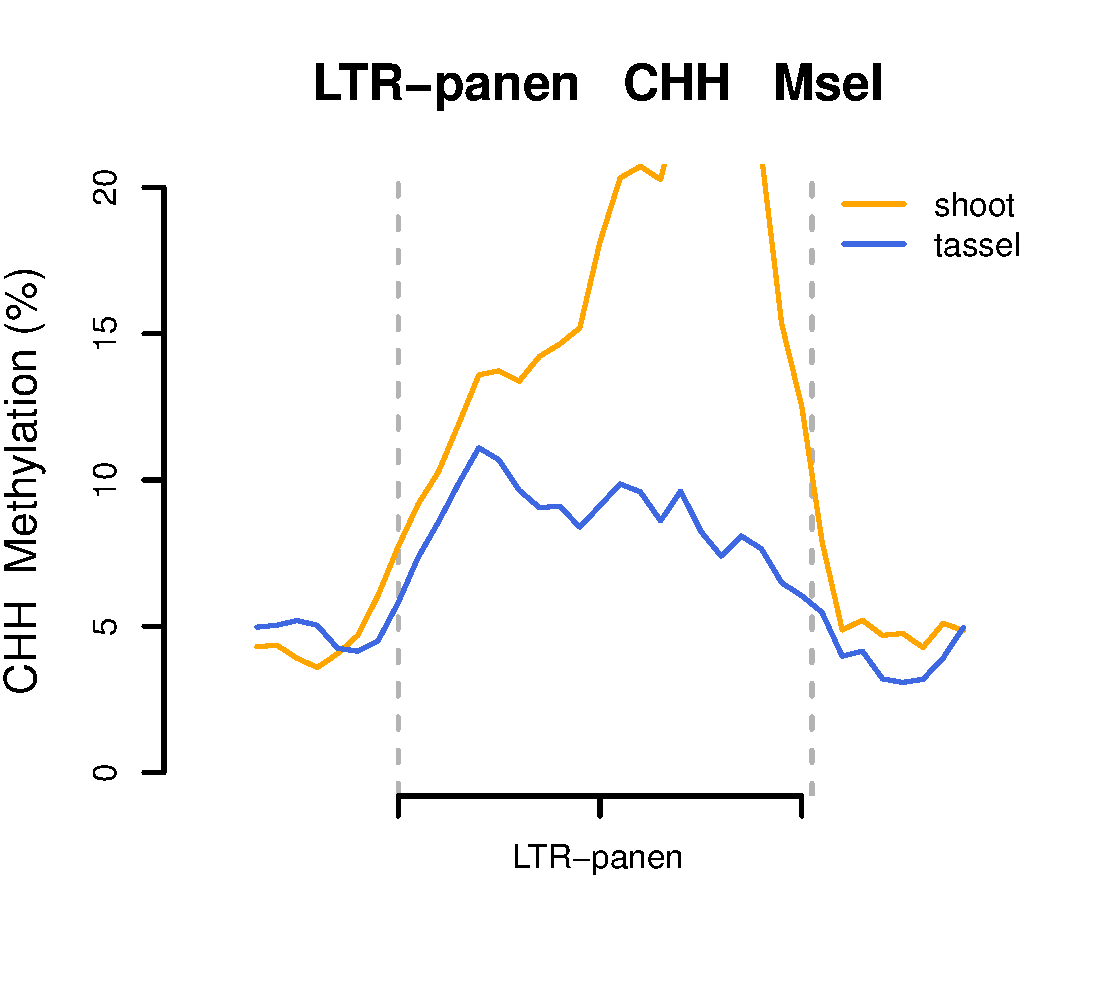

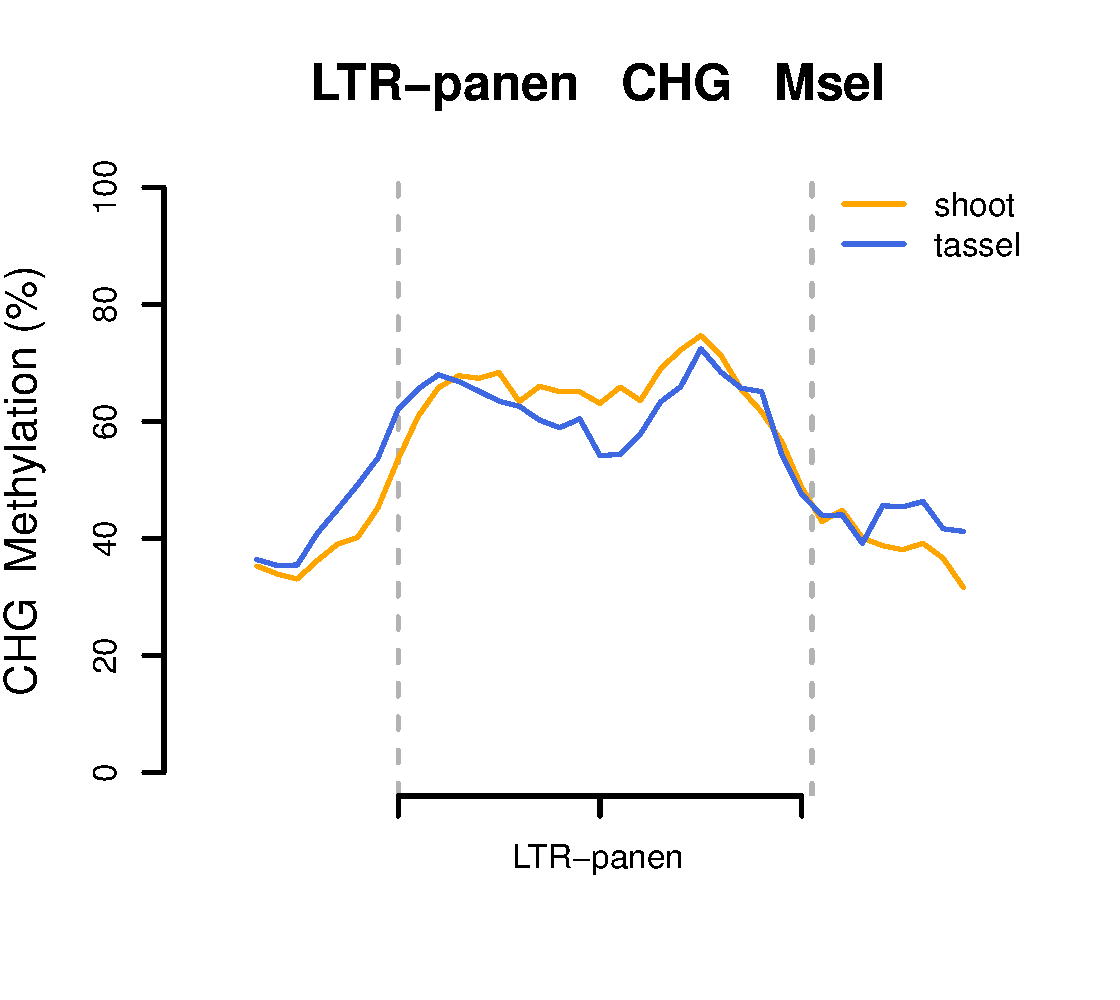

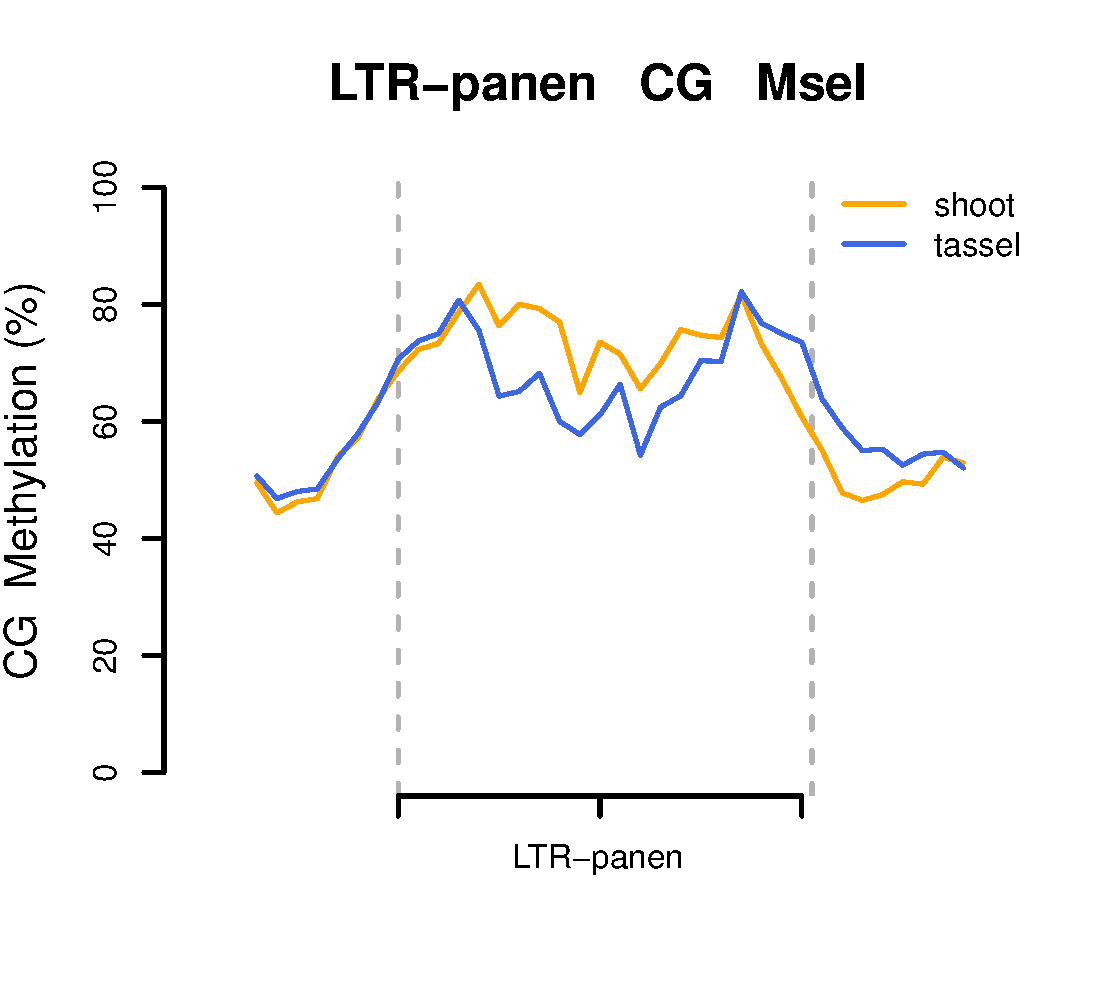


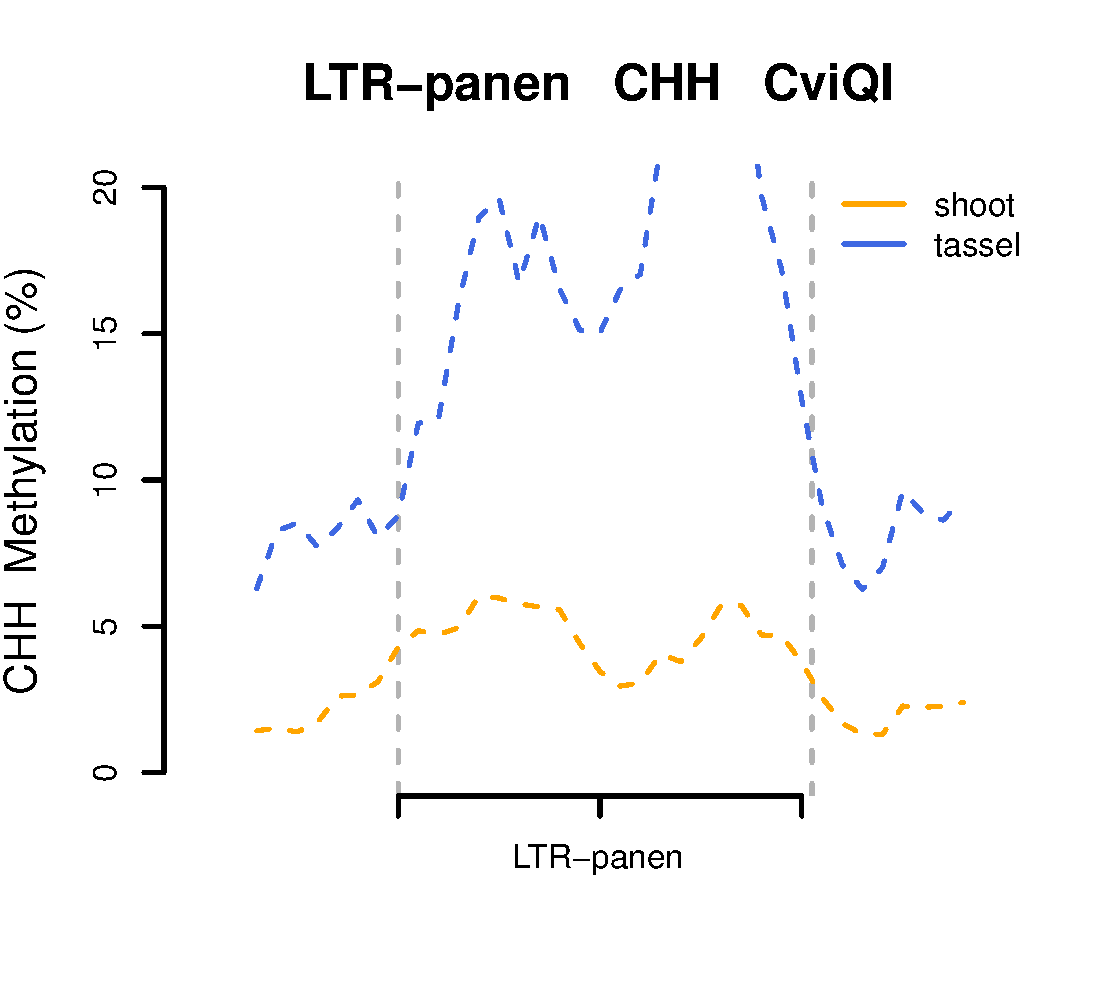

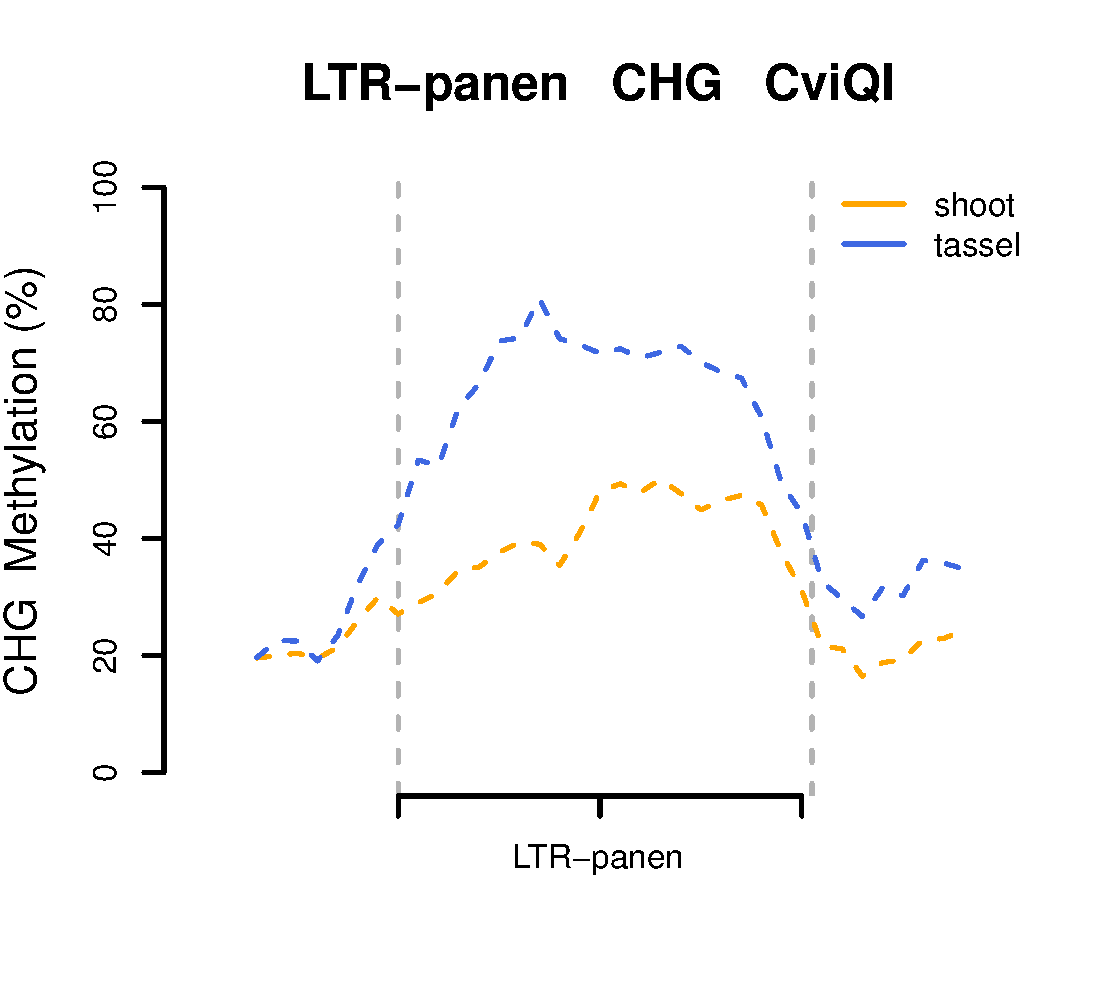

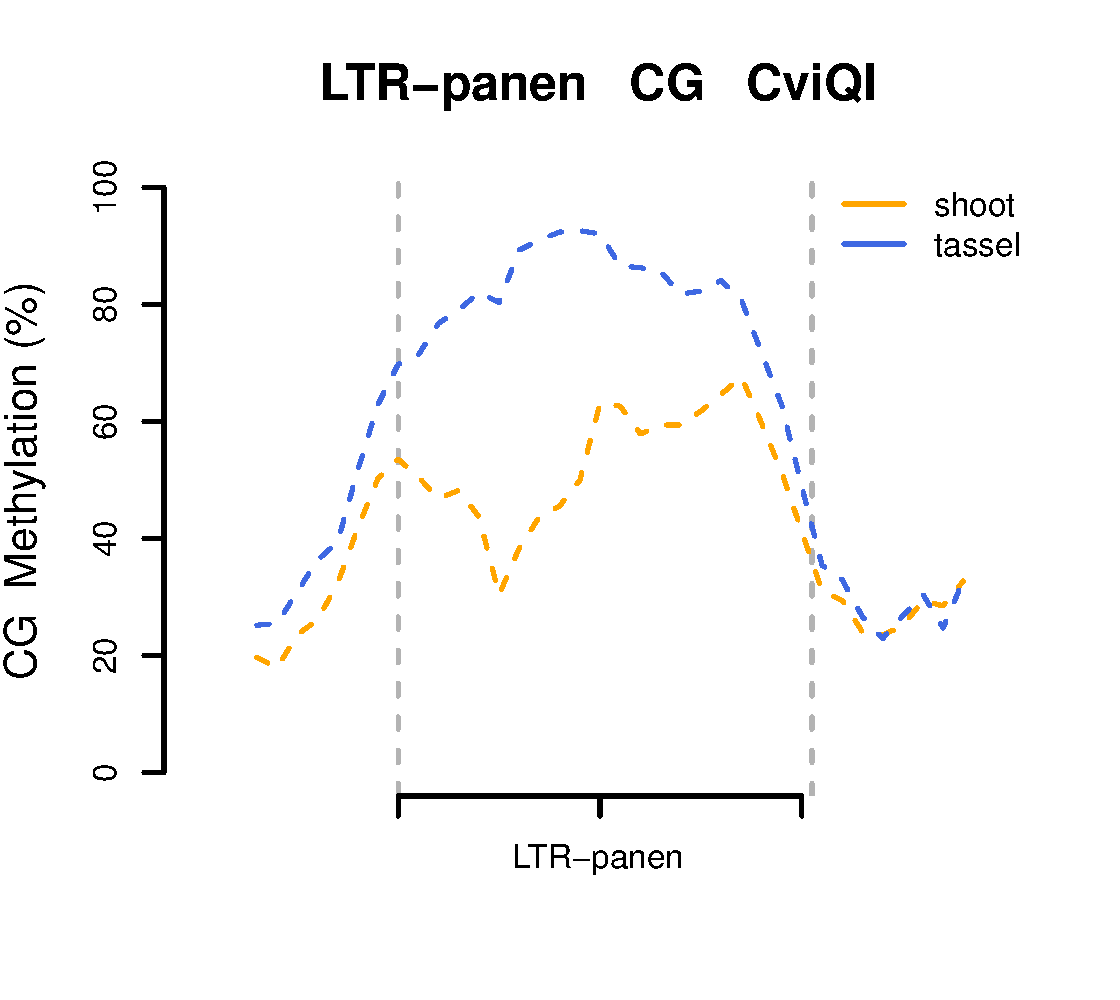


**Figure legend**

**LTR-panen shows different trend between shoot and tassel in *MseI*- and *CviQI*-RRBS.**

1. Schnable PS, Ware D, Fulton RS, Stein JC, Wei F, Pasternak S, Liang C, Zhang J, Fulton L, Graves TA, et al: **The B73 maize genome: complexity, diversity, and dynamics.** *Science* 2009, **326:**1112-1115.
